# A taxonomic revision of the subfamily Tillinae Leach *sensu lato* (Coleoptera, Cleridae) in the New World

**DOI:** 10.3897/zookeys.179.21253

**Published:** 2017-12-07

**Authors:** Alan Burke, Gregory Zolnerowich

**Affiliations:** 1 Department of Zoology and Entomology, University of Pretoria, Hatfield, South Africa; 2 Department of Entomology, Kansas State University, Manhattan, Kansas, USA

**Keywords:** taxonomy, Cleridae, Tillinae, New World, distribution, description

## Abstract

The subfamily Tillinae Leach is represented by 12 genera in the New World. In this study, eight of these genera are revised. A diagnosis and redescription of the species of *Araeodontia* Barr, *Barrotillus* Rifkind, *Bogcia* Barr, *Cylidrus* Latreille, *Cymatoderella* Barr, *Lecontella* Wolcott & Chapin, *Monophylla* Spinola, and *Onychotillus* Chapin are presented. *Bogcia oaxacae* Barr is designated as a junior synonym of *Bogcia disjuncta* Barr. One species, *Cymatodera striatopunctata* Chevrolat, is transferred to *Lecontella*. The following species are redescribed: *Araeodontia isabellae* (Wolcott), *A. marginalis* Barr, *A. peninsularis* (Schaeffer), *Barrotillus kropotkini* Rifkind, *Bogcia disjuncta* Barr, *Cylidrus abdominalis* Klug, *Cymatoderella collaris* (Spinola), *C. morula* Rifkind, *C. patagoniae* (Knull), *Lecontella brunnea* (Spinola), *L. gnara* Wolcott, *L. striatopunctata* (Chevrolat), *Monophylla californica* (Fall), *M. pallipes* Schaeffer, *M. terminata* (Say), *Onychotillus vittatus* Chapin, and *O. cubana* De Zayas. Transcriptions of the original descriptions of *Araeodontia picipennis* Barr, *Bostrichoclerus bicornis* Van Dyke and *Monophylla cinctipennis* (Chevrolat) are given. *Cymatodera* Gray, with approximately 130 described species, is excluded from this study due to the number of species involved. The genera *Neocallotillus* Burke and *Callotillus* Wolcott are also excluded here since these groups have been recently revised elsewhere. Collection data are provided for all species revised. Updated distribution maps are presented. Keys to New World genera and species are given and taxonomic characters of relevant importance are provided and discussed.

## Introduction

Cleridae is a family of predatory beetles with a cosmopolitan distribution (Corporaal 1950; Gerstmeier and Weiss 2009, Gerstmeier and Eberle 2011, Opitz 2010). Opitz (2010) has indicated that clerids can be distinguished from other beetle families within the superfamily Cleroidea based on the existence of a postgular plate or postgular process. This plate or process is present in all clerid species, but absent in remaining cleroid families (Fig. 6A–B). The current classification of Cleridae divides the group in 13 subfamilies (Kolibáč 1997, Opitz 2010, Gunter et al. 2013), with Tillinae the second largest after Clerinae, with approximately 700 described species in 70 genera (Corporaal 1950, Barr 1975, Gerstmeier 2014, Opitz 2010, Burke and Zolnerowich 2016). In the New World, Tillinae is distributed from southern Canada to central South America, including the West Indies (Fig. 21A–L), and is represented by 12 genera: *Araeodontia* Barr, *Barrotillus* Rifkind, *Bogcia* Barr, *Bostrichoclerus* Van Dyke, *Callotillus* Wolcott, *Cylidrus* Latreille, *Cymatodera* Gray, *Cymatoderella* Barr, *Lecontella* Wolcott & Chapin, *Monophylla* Spinola, *Neocallotillus* Burke, and *Onychotillus* Chapin (Corporaal 1950, Barr 1975, Opitz 2010, Burke et al. 2015, Burke and Zolnerowich 2016).

Historically, the description and classification of species within Tillinae have been established on a limited number of external morphological characters, primarily using antennal gestalt, elytral configuration, and overall integument color, with many descriptive works poorly detailed. A number of species within the New World Tillinae are difficult to identify due to intraspecific morphological variation, a situation particularly common for the speciose *Cymatodera*, where, to date, almost 130 taxa have been described (Burke et al. 2015). A figure that, by itself, represents almost 20% of all described Tillinae species. In addition, most of the descriptions and keys to species are based on one or a few specimens, and intraspecific variation has not been examined in detail. A revision of the genus represents a major challenge because many species are poorly represented in public and private collections, numerous species are rare in nature, and comparisons with types are particularly difficult due to the rarity and unavailability of this material.

Four revisionary works (Barr 1952a, Burke and Zolnerowich 2016, Gerstmeier and Weiss 2009, Solervicens 1996) pertaining to Tillinae have been conducted, and only those of Barr (1952a) and Burke and Zolnerowich (2016) addressed tillinid species inhabiting the Americas. In the New World, a relatively small number of taxonomic studies of the Tillinae have been conducted, with authors such as Barr (1947, 1950a, 1950b, 1952a, 1952b, 1962, 1972, 1975, 1978), Chapin (1927, 1945, 1949), Knull (1934, 1940, 1946, 1951), Rifkind (1993a, 1993b, 1995, 1996, 2015), Schaeffer (1904, 1905, 1908, 1917), and Wolcott (1909, 1910, 1911, 1921, 1923, 1927, 1947) the principal contributors to the current knowledge of the subfamily in the Americas.

Due to the complex taxonomic status and great number of species comprising *Cymatodera*, that genus will be revised separately in future works. The genera *Callotillus* and *Neocallotillus* were recently revised by Burke and Zolnerowich (2016) and are also excluded from this study. The work presented here is intended to be a contribution toward a better understanding of the species of Tillinae inhabiting the New World.

## Material and methods

Twenty-two species representing nine of the 12 tillinid genera inhabiting the Americas are treated here. *Cylidrus abdominalis* Klug, a species that is very likely an introduction from the Old World (Gorham 1876) and is established in Brazil (Corporaal 1950), is redescribed. Material from the monotypic species *Bostrichoclerus bicornis* Van Dyke, *Araeodontia picipennis* Barr, and *Monophylla cinctipennis* (Chevrolat) was not available for study, but the original descriptions are transcribed here. New country records are indicated with an asterisk following the corresponding country.

If more than one male per species was available, and upon permission from the corresponding repository collections or private owners, male genitalia were extracted and dissected from selected specimens. Genitalia extraction and dissection procedures are similar to those outlined by Ekis (1977). Most morphological terminology follows the work of Ekis (1977), Rifkind (1993b) and Opitz (2010). Material was examined with a Leica MZ7.5 stereomicroscope. Images were taken and measured using a Leica DFC 500 digital camera, and stacked using the software Zerene Stacker V. 1.04. Scanning electron photographs were taken using a Hitachi 3500N variable pressure scanning electron microscope.

The following codens refer to public or private collections from which material was obtained and examined:

**AMNH** American Museum of Natural History, Washington D.C.

**UAIC** University of Arizona Insect Collection, Tucson, Arizona

**BMNH** British Museum of Natural History Collection, London, UK

**CASC** California Academy of Sciences Insect Collection, Sacramento, CA

**UAMC** Colección de Insectos de la Universidad Autónoma de Morelos, Mexico

**CNIN** Colección Nacional de Insectos UNAM, Distrito Federal, México

**CSUC** Colorado State University Insect Collection, Fort Collins, Colorado

**FMNH** Field Museum of Natural History

**FSCA** Florida State Collection of Arthropods, Gainesville, FL

**INBIO** Instituto Nacional de Biodiversidad, Heredia, Costa Rica

**IRSNB** Institut Royal des Sciences Naturelles de Belgique, Brussels, Belgium

**JNRC** Jacques Rifkind Collection, Valley Village, CA

**JEWC** James E. Wappes Collection, San Antonio, TX

**KSUC** Kansas State University Museum of Entomological Collection, Manhattan, KS

**MNHN** Muséum National d’Histoire Naturelle, Paris, France

**MRAC** Musée Royal de l’Afrique Centrale, Tervuren, Belgium

**LACM** Natural History Museum of Los Angeles, California

**OSUC** Ohio State University Collection, Columbus, Ohio

**RHTC** Robert H. Turnbow Collection, Enterprise, AL

**TAMU** Texas A&M Insect Collection, College Station, TX

**UFBI** Università di Firenze Collezione, Florence, Italy

**UAIC** University of Arizona Insect Collection, Tucson, AZ

**EMEC** University of California, Essig Museum of Entomology, Berkeley, CA

**UGCA** University of Georgia Insect Collection, Athens, GA

**SEMC** University of Kansas, Snow Entomological Museum, Lawrence, KS

**USUC** Utah State University Collection, Logan, UT

**WOPC** Weston Opitz Collection, Salina, KS

**WFBM** William F. Barr Museum, University of Idaho, Moscow, ID

## Taxonomy

### Tillinae

Taxon classificationAnimaliaColeopteraCleridae

Leach, 1815

#### Type genus.

*Tillus* Olivier, 1790.

#### Synonyms.

*Tilloides* Spinola, 1841 (pars) Rev. Zool. IV, p. 71; *Cleroides* Spinola, 1844 (pars) Clérites I, p. 48; Cleridae Desmarest, 1860 (pars) in Chenu; Encycl. d’Hist. Nat. Col. II, p 231; Tillini Lohde, 1900, Stett. Ent. Zeitg., LXI, P. 6; Tillinae Schenkling, 1906. Deutsche Ent. Zeitschr., p. 242.

#### Differential diagnosis.

**Figure 1.**
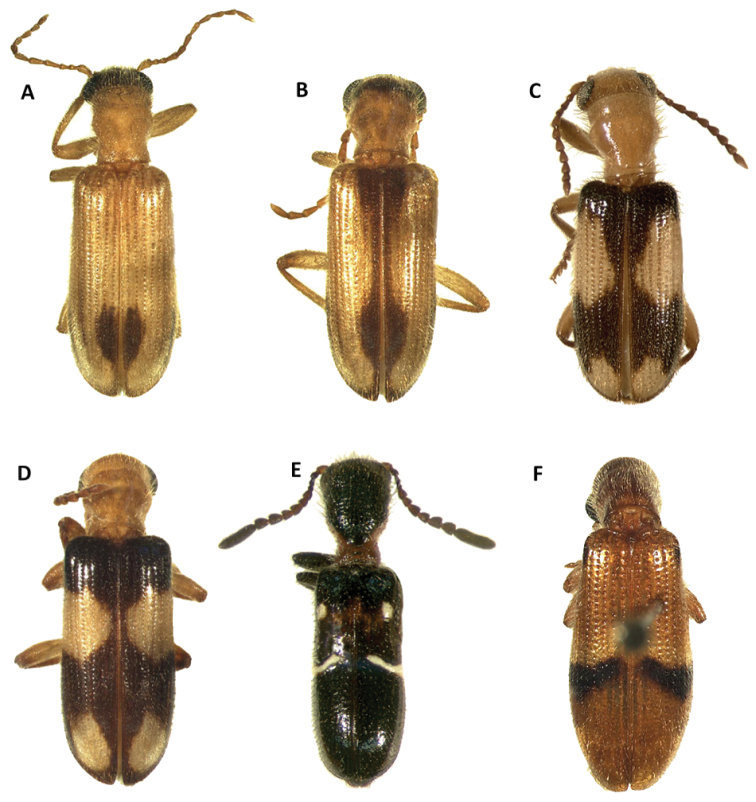
Habitus of: **A** *Araeodontia isabellae* **B** *Araeodontia marginalis* **C** *Araeodontia peninsularis* **D** *Araeodontia picta* **E** *Barrotillus kropotkini* **F** *Bogcia disjuncta*.

**Figure 2.**
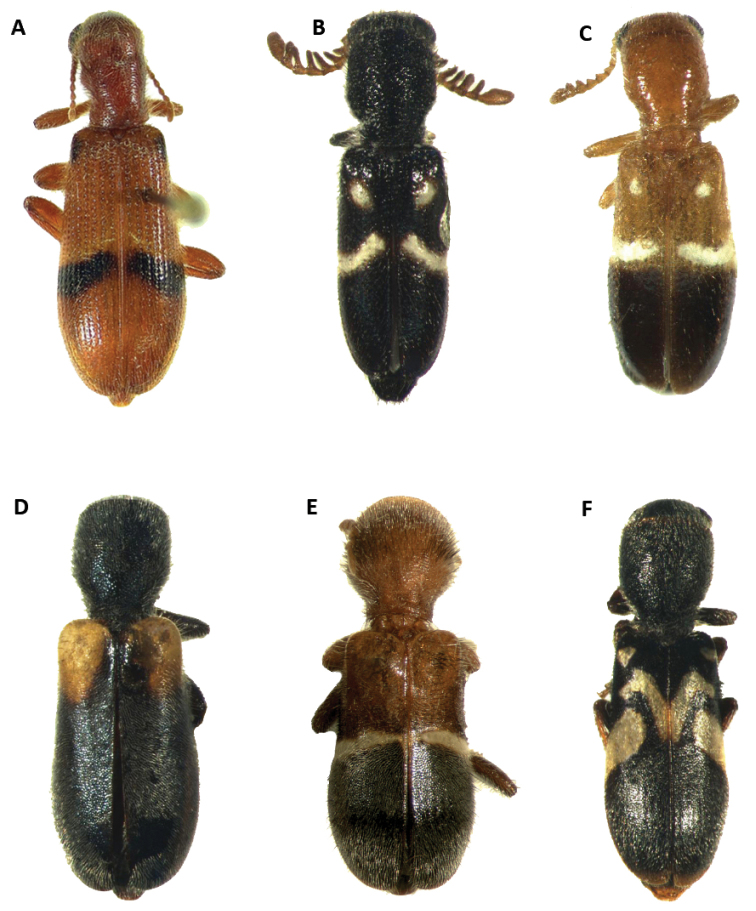
Habitus of: **A** *Bogcia oaxacae* syn. n. **B** *Neocallotillus elegans (bla*ck morph) **C** *Neocallotillus elegans* (bi-colored morph) **D** *Callotillus bahamensis* **E** *Callotillus eburneocinctus* **F** *Neocallotillus intricatus*.

**Figure 3.**
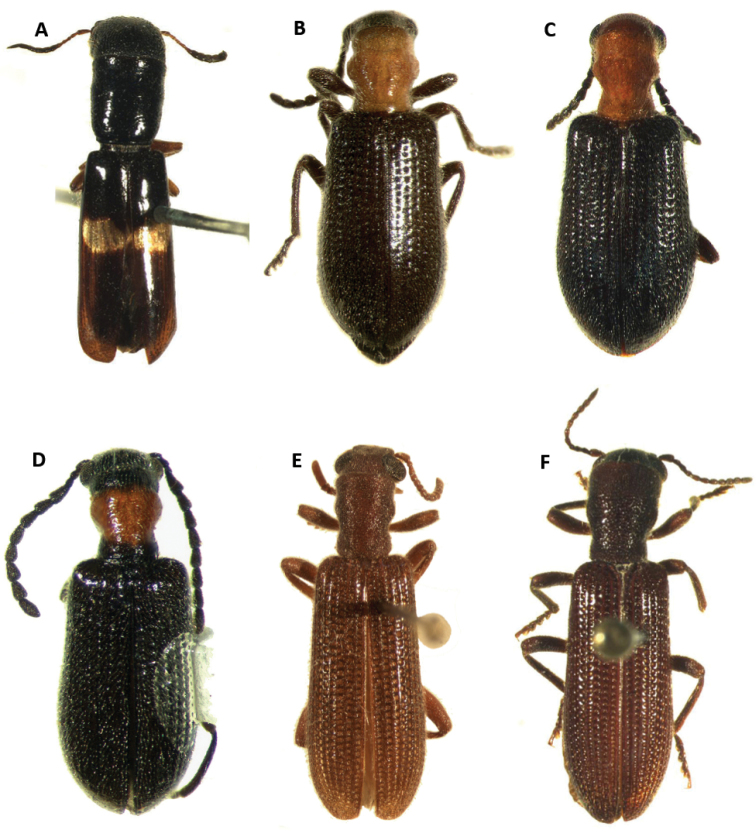
Habitus of: **A** *Cylidrus abdominalis* **B** *Cymatoderella collaris* **C** *Cymatoderella morula* **D** *Cymatoderella patagoniae* **E** *Lecontella brunnea* **F** *Lecontella gnara*.

**Figure 4.**
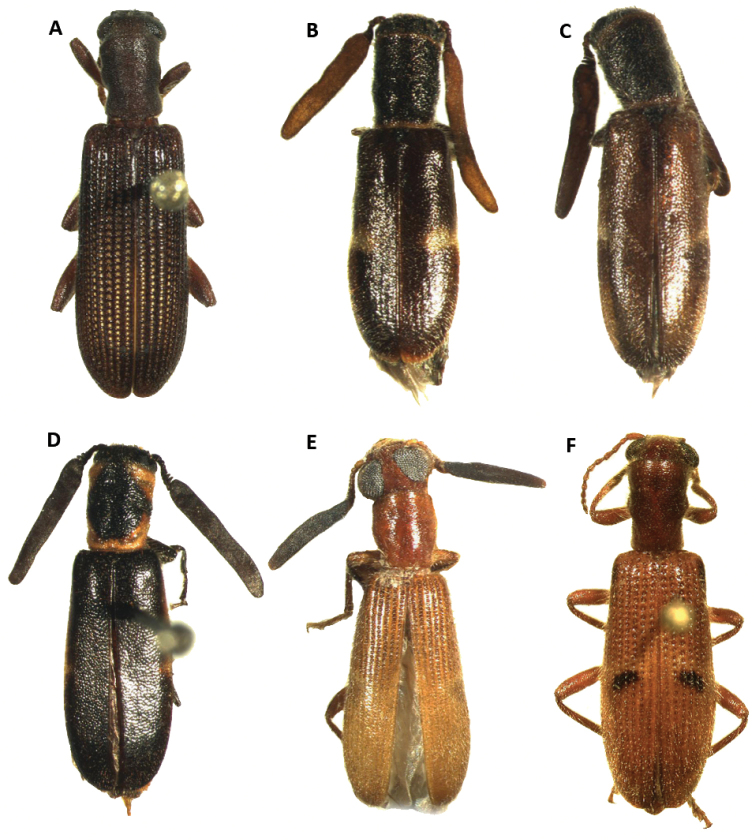
Habitus of: **A** *Lecontella striatopunctata* comb. n. **B** *Monophylla californica* **C** *Monophylla pallipes* **D** *Monophylla terminata* **E** *Teloclerus compresicornis* **F** *Cymatodera bogcioides*.

**Figure 5.**
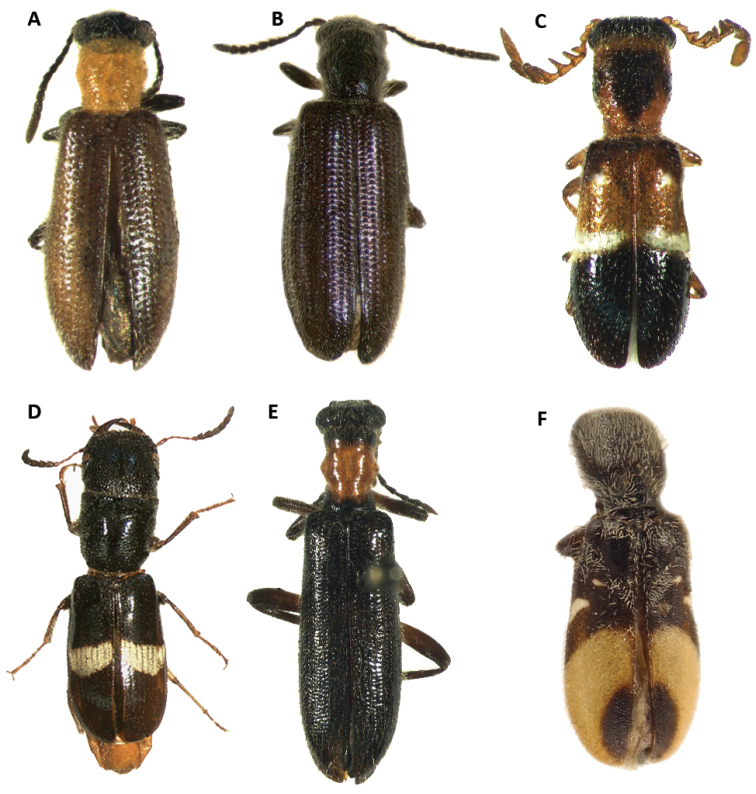
Habitus of: **A** *Onychotillus cubana* **B** *Onychotillus vittatus* **C** *Neocallotillus elegans* **D** *Cylidrus fasciatus* **E** *Cymatodera bicolor* **F** *Neocallotillus crusoe* (image courtesy of The American Museum of Natural History, New York).

**Figure 6.**
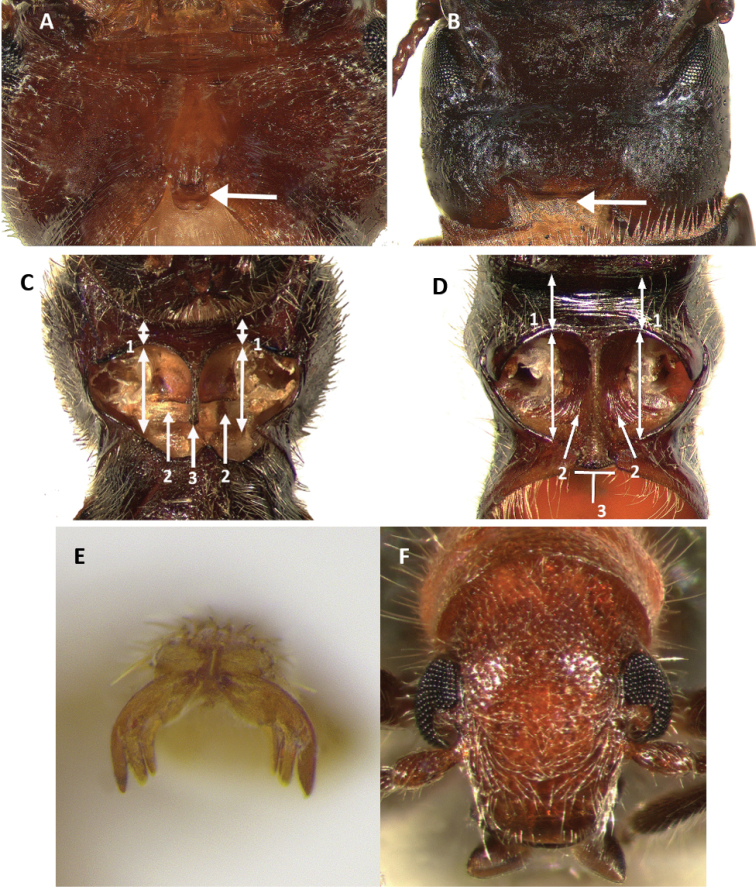
**A–B** Gular structure of: **A** *Cymatodera californica*, arrow indicates post-gular process present **B** *Temnoscheila virescens* (Trogossitidae), arrow indicates post-gular process absent **C–D** Procoxal cavities of: **C** *Enoclerus zonatus* **D** *Cymatodera sallei*; arrows 1 indicate longitudinal length of procoxal cavities in relation to longitudinal length of prosternum; arrows 2 indicate interior portion of procoxal cavities; arrow 3 indicates intercoxal process **E** Tarsal claw of *Araeodontia peninsularis* **F** Eye structure of *Cymatoderella collaris*.

**Figure 7.**
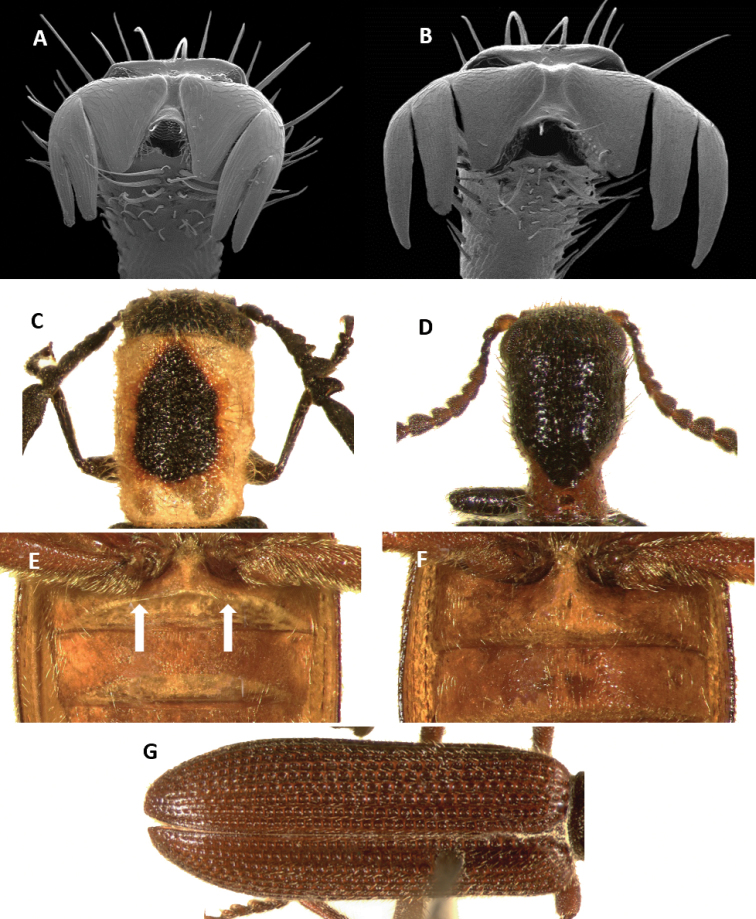
**A–B** Tarsal claws of: **A** *Bogcia oaxacae* **B** *Cymatodera balteata* **C–D** Pronotal structure of: **C** *Monophylla terminata* **D** *Barrotillus kropotkini* **E–F** First and second visible ventrite of: **E** *Cymatodera mitae* (male), arrows indicate longitudinal carina **F** *Cymatodera mitae* (female) **G** Elytral ground of *Lecontella gnara*.

**Figure 8.**
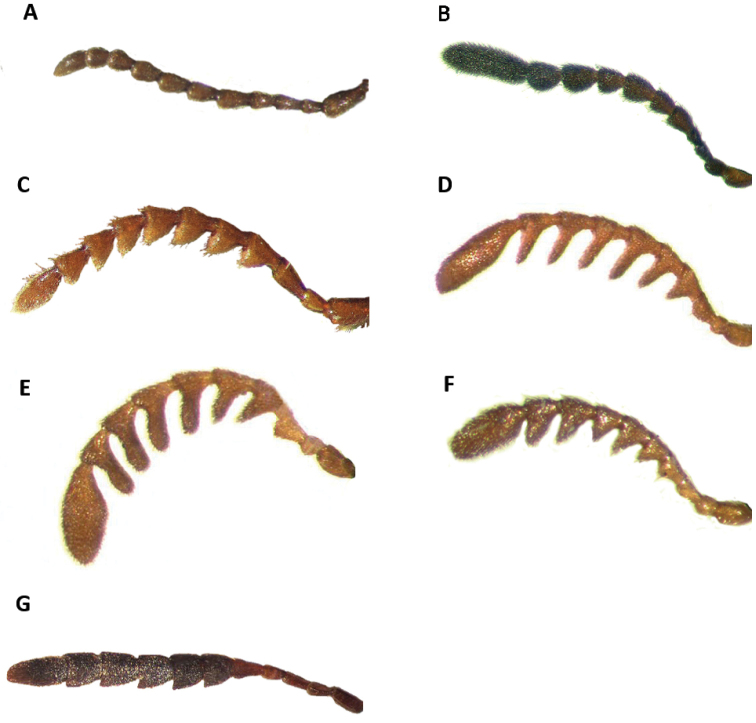
Antennae of: **A** *Araeodontia peninsularis* (male) **B** *Barrotillus kropotkini* (male) **C** *Bogcia oaxacae* (male) **D** *Neocallotillus elegans* (*elegans*) (male) **E** *Neocallotillus elegans* (*vafer*) (male) **F** *Neocallotillus elegans* (*elegans*) (female) **G** *Cylidrus abdominalis* (male).

**Figure 9.**
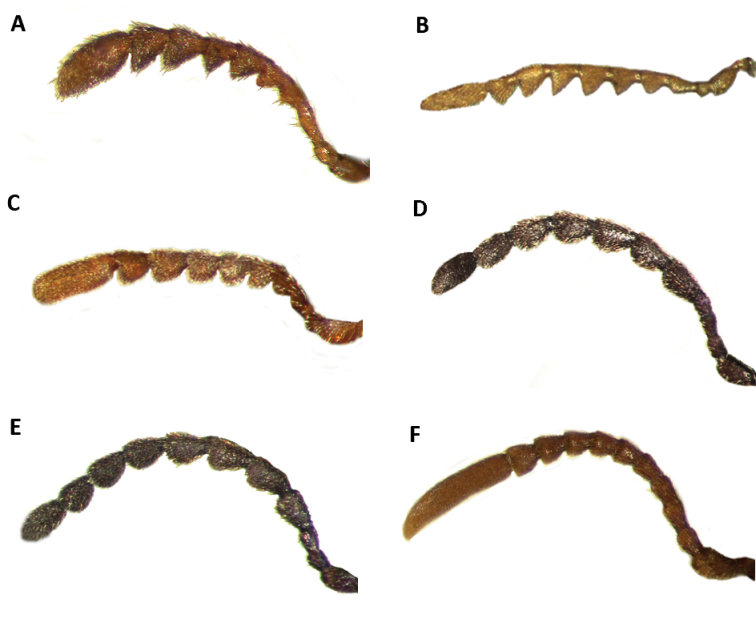
Antennae of: **A** *Neocallotillus elegans* (*vafer*) (female) **B** *Callotillus eburneocinctus* (male) **C** *Callotillus eburneocinctus* (female) **D** *Cymatoderella collaris* (male) **E** *Cymatoderella morula* (male) **F** *Lecontella brunnea* (male).

**Figure 10.**
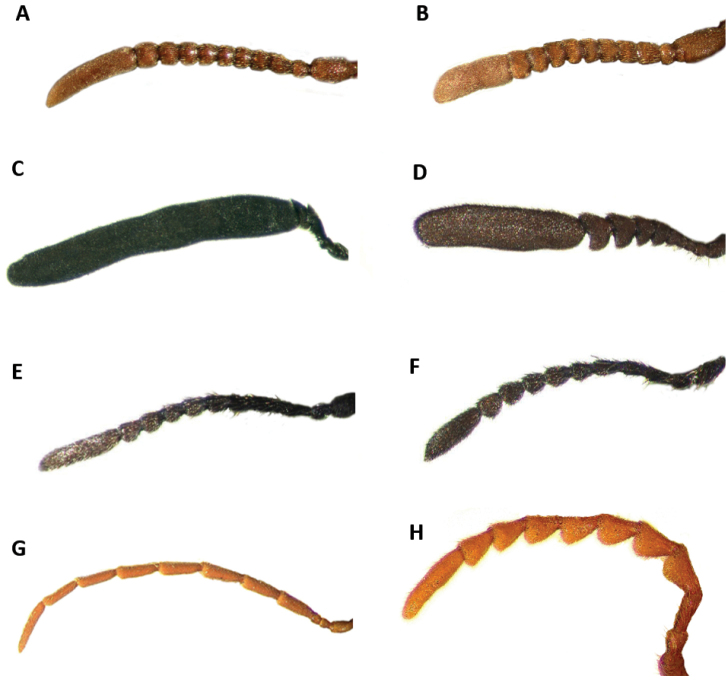
Antennae of: **A** *Lecontella gnara* (male) **B** *Lecontella striatopunctata* (male) **C** *Monophylla californica* (male) **D** *Monophylla terminata* (female) **E** *Onychotillus cubana* (male) **F** *Onychotillus vittatus* (male) **G** *Cymatodera longicornis* (male) **H** *Cymatodera limatula* (male).

**Figure 11.**
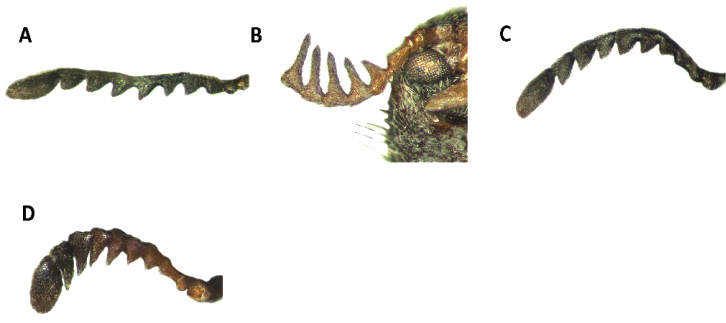
Antennae of: **A** *Callotillus bahamensis* (male) **B** *Neocallotillus intricatus* (male) **C** *Callotillus bahamensis* (female) **D** *Neocallotillus intricatus* (female).

**Figure 12.**
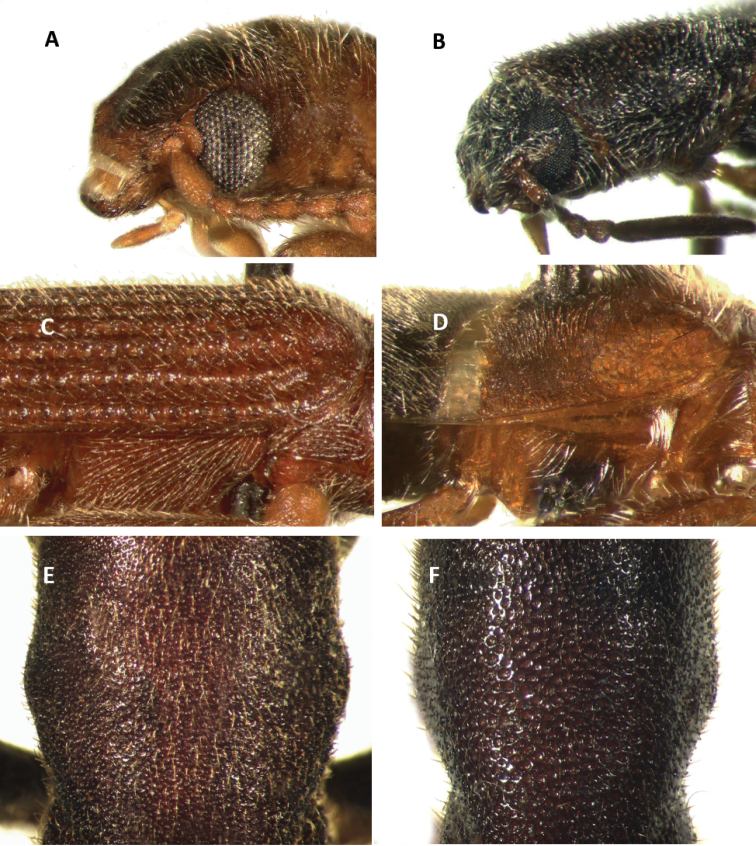
**A–B** Eye structure of: **A** *Cymatodera laevicollis* **B** *Monophylla californica* **C–D** Mesepisternum: **C** Hidden throughout its length in *Lecontella gnara* **D** Visible throughout its length in *Callotillus eburneocinctus* **E–F** Pronotal surface of: **E** *Lecontella brunnea* **F** *Lecontella gnara*.

**Figure 13.**
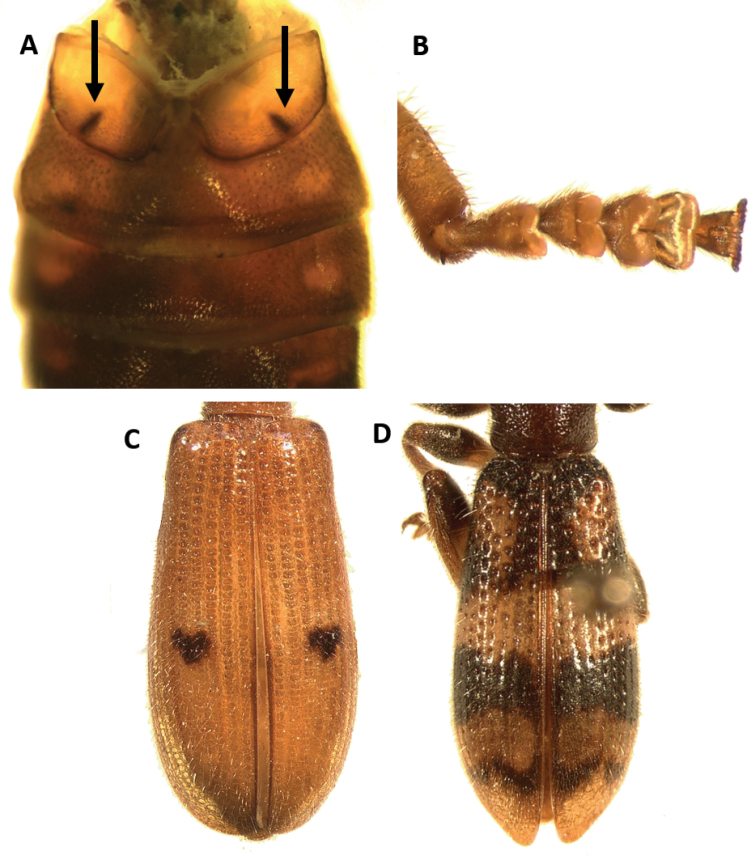
**A** Abdomen of *Cymatodera balteata*, arrows indicate first abdominal segment bearing a pair of carinae **B** Protarsomere of *Cymatodera tuta* **C–D** Elytral punctations of **C** C*ymatodera bipunctata* **D** *Cymatodera grossa*.

Tillinae is characterized by the fusion of the procryptosternum with the pronotal extension, a character that distinguishes this group of checkered beetles from other Cleridae (Fig. 6C–D). Secondary characters that readily differentiate Tillinae from other clerid subfamilies are: body oblong, narrow to robust (Figs 1–5); eyes most often with coarse ommatidia (Fig. 12A); antennae consisting of 9 to 11 antennomeres (Figs 8–11); pronotum campanulate to bisinuate (Figs 5E, 7 C–D, 12E–F); procoxal cavities closed internally and posteriorly (Fig. 6D), one longitudinal carina on the anterior portion of each metacoxal cavity (Fig. 13A); dorsolateral ridge absent (Figs 12A–B); and tarsal formula 5-5-5 (Fig. 13B).

#### Redescription.

Body form: Slender to moderately robust (Figs 1E, 2E, 3B–D) oblong, elongate to short. Pronotum: oblong, long, constricted posteriorly and sometimes anteriorly; anterior and posterior margins truncate; lateral margins parallel, sinuate or bisinuate (Figs 3E, 5A, 7C–D). Size: 3–40 mm. Integument color: From black to piceous and light piceous, with some metallic tones. Elytral fasciae with predominant hues of testaceous, brown, ferrugineous and/or yellow hues (Figs 2D–E, 3A, 4B–C, 5F).

Head: Large to very large; epistomal sutures parallel to feebly sinuate, well developed and extended posteriorly; clypeus well developed; eyes small to very large, always emarginate anteriorly, moderately to strongly emarginate; ommatidia slightly to coarsely faceted (Figs 6F, 5A–B); gula broad, extended posteriorly; postgular process well developed (Fig. 6A); antennae composed of 9 to 11 antennomeres (Figs 8E–F, 9E–F); antennal shape from filiform to pectinate, with various degrees of serration observed, rarely capitate (Figs 8E–G, 9B, 10G–H); mandibles well developed, stout; maxilla with well-developed laterolacinia; terminal labial palpi digitiform to cylindrical; terminal maxillary palpi cylindrical to securiform; labium developed.

Thorax: Pronotum ranging from long bisinuate to campanulate to subquadrate (Figs 5A–E, 7C–D); dorsolateral carinae absent (Fig. 12A–B); abdominal sutures complete; prosternum longitudinally expanded anteriorly; prointercoxal process expanded anteriorly, closed internally and posteriorly. Mesoventrite cylindrical; punctations on elytral disc bearing setae; punctations may reach apex or not; epipleural fold developed and positioned laterally.

Legs: With tarsal pulvilli well developed, fourth tarsomere never reduced (Fig. 13B); tarsal claws well developed, with one or two tarsal denticles (Figs 6E, 7A–B); tarsal formula 5-5-5; tibia and femora about the same length; tibial spur formula 2-2-2, 0-2-2, 2-1-1, or 0-0-0; tarsal pulvilliar formula 4-4-4, 4-4-3, 4-3-3, or 4-2-1; posterior wing venation well developed.

Abdomen: Six visible ventrites. First ventrite almost always longitudinally carinate proximal to metacoxal cavities (Fig. 13A). Sixth visible ventrite incised distally or not; spicular fork well developed, plates developed, intraspicular plate expanded anteriorly;

Aedeagus: Feebly to strongly sclerotized, phallobasic apodeme complete, phallobase acuminate distally; internal ovipositor elongate, usually as long as length of abdomen (Figs 18A, 20C).

#### Key to New World genera updated and modified from Opitz (2002) and Burke et al. (2015)

**Table:** 

1	Anterior coxal cavities opened internally and posteriorly (Fig. 6C)	**non-TillinaeCleridae**
–	Anterior coxal cavities closed internally and posteriorly (Fig. 6D)	**Tillinae** (2)
2(1)	Frons with a pair of prominent horns arising immediately above eyes	***Bostrichoclerus***
–	Frons without a pair of prominent horns	**3**
3(2)	Head subquadrate, conspicuously enlarged throughout its length, as wide as or wider than pronotum; body integument feebly clothed (Figs 3A, 5D)	***Cylidrus***
–	Head not subquadrate, somewhat enlarged throughout its length (Figs 3B–F, 14A–B); body moderately to conspicuously clothed (Figs 2C–F, 4A, 6F)	**4**
4(3)	Last antennomere flattened laterally, much longer than length of preceding antennomeres combined (Figs 4B–D, 10C–D)	***Monophylla***
–	Last antennomere not flattened laterally, not enlarged; length of last antennomere shorter than length of preceding antennomeres combined (Figs 8–9)	**5**
5(4)	Antennae composed of 10 antennomeres (Figs 11A, C–D); mesanepisternum visible in lateral view (Fig. 12-D)	**6**
–	Antennae composed of 11 antennomeres (Fig. 10A–B, G–H); mesanepisternum concealed in lateral view (Fig. 12C)	**7**
6(5)	Slender species (Fig. 2B–C); elytra in lateral view flat; male and female pygidia not modified (Figs 16 G–L, 17A–B); frons wide (Fig. 14A); simple aedeagus (Figs 15A–C, 19A)	***Neocallotillus***
–	Robust species (Figs 2D–E); elytra in lateral view moderately to strongly compressed medially; frons narrow (Fig. 14B); simple aedeagus (Fig. 15D)	***Callotillus***
7(5)	Antennomeres 4-10 strongly serrate (Fig. 8C); length of specimens approximately 7 to 10 mm	***Bogcia***
–	Antennomeres 4-10 slightly to moderately serrate (Figs 9E–F; 10E–H) but never strongly serrate; length of specimens 2–40 mm	**8**
8(7)	Tarsal claws with one inner denticle	***Onychotillus***
–	Tarsal claws with two inner denticles (Figs 6E, 7A–B)	**9**
9(8)	Basal denticle of tarsal claws digitiform (Fig. 6E)	***Araeodontia***
–	Basal denticle of tarsal claw trigonal (Fig. 7A–B)	**10**
10(9)	Elytral punctations coarse, elytral striae extending to apex of elytra (Figs 3E–F, 4A, 7G)	***Lecontella***
–	Elytral punctations very feebly to moderately impressed, elytral striae not extending to apex of elytra (Figs 1A–D; 13D)	**11**
11(10)	Elytral disc with a pair of pale, oblique, elevated fasciae, and a pair of pale, elevated maculae (Fig. 1E); antennae with 10 antennomeres (Fig. 8B); small specimens	***Barrotillus***
–	Elytral disc without elevated fasciae or maculae, small to very large specimens (Figs 3B–D; 5E; 13C–D)	**12**
12(11)	Ommatidia finely faceted (Fig. 6F); small individuals; without lateral carina on first visible ventrite	***Cymatoderella***
–	Ommatidia coarsely faceted (Fig. 12A); small to very large individuals; with or without lateral carina on first visible ventrite (Fig. 7E–F)	***Cymatodera***

### Araeodontia

Taxon classificationAnimaliaColeopteraCleridae

Barr, 1952a

#### Type species.

*Cymatodera peninsularis* (Schaeffer, 1904), original designation.

#### Distribution.

Shown in Fig. 21B.

#### Differential diagnosis.

Members of *Araeodontia* can be separated from the similar *Cymatodera* by the structure of the protarsal claws. The basal denticles of the protarsal claws in *Araeodontia* are digitiform (Fig. 6E), while members of *Cymatodera* have these denticles trigonal (Fig. 7B).

#### Redescription.

Size: 6–12 mm. Color: light testaceous to dark brown, fasciae on elytral disc ranging from testaceous to dark brown. Body: Winged species, somewhat elongate, robust.

Head: Including eye width wider than pronotum; integument smooth to feebly punctate; eyes large, coarsely faceted, feebly emarginate anteriorly; antennae filiform to somewhat serrate, composed of 11 antennomeres, reaching posterior half of pronotum; frons can be bi-impressed or not; terminal labial palpi securiform; terminal maxillary palpi cylindrical, compressed laterally.

Thorax: Pronotum smooth to feebly punctate, widest at middle, sides more constricted behind middle. Prosternum smooth to slightly punctate. Mesoventrite feebly to strongly punctate. Metaventrite slightly punctate, glabrous to conspicuously vested; metaventral process not compressed anteriorly. Metanepisternum concealed throughout its length in lateral view.

Elytra: Elongate, subparallel, slightly broader behind middle; surface feebly punctate, punctations extending to posterior third but never reach apex; scutellum ovoid, not compressed; vested; epipleural fold complete, narrowing toward apex.

Legs: Moderately to coarsely rugose; feebly vested; profemora slightly swollen; pulvillar formula 4-4-4; two tarsal denticles, tarsal denticles digitiform in shape (Fig. 6E).

Abdomen: Six visible ventrites. Ventrites 1-5 impressed laterally or not. Pygidium of males somewhat differentiated from that of females (Fig. 16A–D); males with sixth ventrite moderately, narrowly V-shaped emarginate (Fig. 16B); pygidium of females simple, broadly rounded (Fig. 16D). Male and female pygidium shape are not variable for all the species in the genus.

#### Remarks.

Barr (1952a) conducted a revisionary work of those *Cymatodera* species possessing digitiform tarsal denticles (Fig. 6E). In this revision, he indicated that, based on the state of the tarsal denticles, these species should be assigned to a different genus. The tarsal denticles of *Cymatodera* are triangular (Fig. 7B); however, this character was inconsistent in three species originally assigned to *Cymatodera* occurring in northern Mexico, Lower California, and the southwestern United States. As a result, the genus *Araeodontia* was erected and two new species, *Araeodontia picta* and *A. marginalis*, were also described. Barr indicated that, based on differences in the structure of the protarsal denticles, *Araeodontia* could be further divided into two separate groups, one solely composed of *A. picta* Barr, and the second composed of the remaining species. In this revisionary work, we examined a significant number of specimens from all *Araeodontia* species, except *A. picipennis*, and while differences in the size of the protarsal denticles exist, they are subtle and there is not a clear division of two separate groups within the genus (Fig. 1A–D).

#### Key to species of *Araeodontia*

**Table:** 

1	Elytra immaculate, uniformly brown to dark brown	***Araeodontia picipennis***
–	Elytra with an array of markings that range from prominent fasciae to maculae, elytral disc light testaceous to dark brown	**2**
2(1)	Each elytron with a longitudinal, light brown macula on the posterior third of the elytral disc (Fig. 1A), this macula never reaches the lateral margin of the elytron	***Araeodontia isabellae***
–	Each elytron with a light testaceous to testaceous fascia that extends from the anterior margin of the elytral disc to the elytral apex (Fig. 1B–D), this fascia may or may not reach the lateral margin of the elytron	**3**
3(2)	Integument color of head brown to dark-brown, darker than the rest of the body; two longitudinal fasciae on each elytron, the first located on elytral suture and the second along epipleural fold, these fasciae may be interconnected on elytral apex or not (Fig. 1B)	***Araeodontia marginalis***
–	Integument color of head the same color as the rest of the body	**4**
4(3)	Elytra with an anterior pair of maculae reaching epipleural fold, these maculae more proximal to the humeri (Fig. 1C)	***Araeodontia peninsularis***
–	Elytra with an anterior pair of maculae that do not reach the epipleural fold, these maculae are more distal to the humeri (Fig. 1D)	***Araeodontia picta***

### Araeodontia isabellae

Taxon classificationAnimaliaColeopteraCleridae

(Wolcott, 1910)

Figs 1A , 18A

#### Synonyms.

*Cymatodera isabellae* Wolcott, 1910. Field Museum Natural History, zool. Ser., vol. 7, no. 10, 9 345. Wickham and Wolcott 1912 University of Iowa Bulletin Laboratory of Natural History, vol. 6, no. 3 p. 52. Wolcott 1921 Proc. U.S. Natl. Mus., vol. 59, p. 285. Barr 1950b, Proc. California Acad. Of Sci., ser. 4, vol. 24, no. 12, p. 496.

#### Type material not examined.

#### Type locality.

United States: Utah, St. George, Washington Co. Type depository: National Museum of Natural History (USNM).

#### Distribution.

USA: AZ, CA, NV, TX, UT.

#### Differential diagnosis.

*Araeodontia isabellae* is most similar to *A. picipennis*. The two species can be distinguished based on the color of the elytral disc and elytral patterning. *Araeodontia isabellae* has the elytral disc pale testaceous to testaceous and possesses two brown to light brown maculae on each elytron (Fig. 1A), while *A. picipennis* has the elytra uniformly brown to dark brown and lacks maculae on the elytral disc.

#### Redescription.

Male. Form: Somewhat slender, slightly elongate. Color: Head, mouthparts and pronotum light testaceous to brown; thorax, elytra, abdomen and legs light testaceous to testaceous; two longitudinal brown to testaceous maculae on the posterior half of each elytron, the first located proximate to the elytral suture, the second adjacent to the epipleural fold, neither of these maculae reach the elytral apex, these maculae can be faint to almost absent in some specimens (Fig. 1A).

Head: Surface moderately to densely punctate; frons bi-impressed; eyes enlarged, bulging laterally, coarsely faceted; antennae extending to posterior half of pronotum; antennomeres 2-3 reduced in length; fourth antennomere about 2× the length of third antennomere; antennomeres 4-10 about the same length as fourth antennomere; antennomeres 4-10 somewhat slender, feebly serrate; eleventh antennomere robust, subacuminate.

Thorax: Pronotum slightly punctate; faintly rugose laterally, smooth; vested by erect and semi-erect setae; broadest at middle; disc flat, indistinctly impressed in front of middle, subbasal tumescence absent. Mesoventrite very slightly punctate, smooth. Metaventrite convex, puncticulate; covered with fine erect and semi-erect setae. Scutellum subquadrate, notched posteriorly.

Legs: Vested with short, recumbent setae intermixed with long, erect setae that become more densely arranged on the distal half of the tibia. Femora rugulose; finely punctate. Tibiae transversely rugose, coarsely punctate, vested with short, recumbent setae intermixed with semi-erect setae.

Elytra: Humeri rounded, indicated; sides subparallel; base wider than pronotum; widest behind middle; disc flattened apically; apices subtriangular, very slightly dehiscent; disc convex, surface rugulose; vested, vestiture composed of erect and semi-erect setae; sculpture consisting of small, coarse punctations arranged in striae that are gradually reduced in size behind middle and do not reach elytral apex; interstices smooth, 3.0× the width of punctuation at anterior margin.

Abdomen: Ventrites 1-4 rugulose, vested with short, recumbent setae and some long, semi-erect setae, indistinctly, finely punctate. First visible ventrite approximately 1.5× the length of second ventrite. Fifth visible ventrite small, convex, lateral margins subparallel, posterior margin broadly, feebly emarginate. Sixth visible ventrite subquadrate, surface somewhat excavated medially, convex laterally; slightly punctate, lateral margins oblique; posterior margin broadly, deeply emarginate, emargination V-shaped, posterolateral angles rounded. Fifth tergite slightly convex, finely punctate, rugulose, lateral margin subparallel, posterior margin broadly, shallowly, very feebly, emarginate. Sixth tergite subtriangular; rugulose; surface convex; longer than broad; finely punctate; inconspicuously covered with short, recumbent setae; lateral margins oblique; posterior margin narrowly, very shallowly emarginate; hind angles rounded. Posterior margin of sixth tergite fully covering sixth visible ventrite and produced ventrally.

Aedeagus: Phallobasic apodeme present; phallus with copulatory piece rounded at apex; phallic plate devoid of denticles; intraspicular plate absent; phallobasic apodeme long, expanded distally; phallobase trigonal; parameres free; tegmen complete, fully covering phallus; parameres pointed anteriorly; endophallic struts long, at least the length of tegmen; endophallic struts slender distally (Fig. 18A).

Sexual dimorphism: Females can be differentiated from males by the shape of the last abdominal segment. In females of *A. isabellae* the last abdominal segment is broadly rounded and convex to almost flat; males have this segment broadly and deeply emarginate posteriorly. The structure of the sixth abdominal segment is very consistent for all females examined.

#### Material examined.

2 males, 3 females: Phoenix, AZ, VIII-23-1932, D. K. Duncan; 2 males, 2 females: Nevada, VII-24-1950; 1 male: Texas, VI-2-1950; 1 male: Phoenix, AZ, 5409, Chas Palm; 2 males: Imperial Co., CA, Calipatria, VI-4-1962, Kilgore; 1 male, 2 females: Riverside Co., CA, Palm Canyon 1000, VII-21-1973, W. Barr; 1 female: Clark Co., NV, Logandale, IX-13-1984, Riley, Nelson and Wheeler; 5 females: Yuma Co., AZ, Morelos Dam, VI-22-1977, E. Giesbert; 1 male, 3 female: Yuma, AZ, Laguna Dam, VIII-9-1954, Butler and Tuttle; 2 males, 1 female: Riverside Co., CA, Blythe, VII-30, 31-1956, Truxal, Honey and Menke; 2 females: Riverside Co., CA, 15 mi N Blythe, VII-12-1977, Schuster and Smith; 3 males: Riverside Co., CA, 12 mi N Blythe, VII-12-1977, P. Bertrand; 3 males: Phoenix, AZ, VIII-31-1935, Parker; 1 female: El Centro [CA], IX-5-1953, Parker; 1 female: 12 mi E of Herbert, V-12-1956, T. R. Haig; 4 males, 5 females: Phoenix AZ, VIII-31-1953, no collector data; 2 males: Clark Co., NV, Logandale, IX-2-1959, E. D. Parker; 1 male: San Diego Co., CA, Anza-Borrego Springs National Park, VI-5-1971, Sweet and Sweet; 3 males, 3 females: Phoenix, AZ, VIII-31-1935, F. H. Parker; 1 female: Riverside Co., CA, 15 mi N of Blythe, VII-12-1977, R. C. Schuster and N. J. Smith; 1 male: Riverside Co., CA, 12 mi N Blythe, VII-12-1977, R. C. Schuster and N. J. Smith; 2 females: Plumas Co., CA, Johnsville, VIII-8-1959, J. S. Buckett; 1 female: Needles, CA, VII-13-1977, R. C. Schuster and N. J. Smith; 5 males, 2 females: Phoenix, AZ, VIII-31-1935, Parker; 2 females: Imperial Co., CA, 12 mi E of Heber, 12-V-1956, T. R. Haig; 1 female: Clarke Co., NV, Logandale, 2-IX-1959, F. D. Parker; 2 males, 2 females: La Paz Co., AZ, 19-VI-1996, Cibola NWR, D. Anderson.

### Araeodontia marginalis

Taxon classificationAnimaliaColeopteraCleridae

Barr, 1952a

Figs 1B , 18B

#### Paratype.

One male examined.

#### Type locality.

Mexico, Samalayuca, Chihuahua. Type depository: American Museum of Natural History (AMNH).

#### Distribution.

USA: TX; Mexico: Chihuahua, Coahuila, Sonora.

#### Differential diagnosis.

*Araeodontia marginalis* is most similar to *A. isabellae*. The fascia pattern on the elytral disc can be used to separate these species. *Araeodontia marginalis* has two longitudinal fasciae that extend from the elytral base to the apex, the first band is located adjacent to the elytral suture and the second runs along the epipleural fold; for some specimens, the latter band can be absent on the anterior half of the elytral disc; both fasciae are interconnected at the apex (Fig. 1B). *Araeodontia isabellae* has the elytral disc uniformly light testaceous and each elytron has one brown macula (Fig. 1A).

#### Redescription.

Male. Form: Body relatively stout, elongate. Color: Head, anterior margin of pronotum and mouthparts brown to dark brown; pronotum, thorax, elytra, abdomen and legs testaceous to light brown; two brown, longitudinal fasciae on each elytron, the first located on the elytral suture, and extends from anterior margin of elytra and reaches apex, this fascia abruptly reduced in width on second and last fourth, the second adjacent to epipleural fold, and also extends from anterior margin of elytron and reaches apex, this fascia may be reduced to absent on the anterior half of elytral length, both fasciae may be interconnected at the elytral apex (Fig. 1B).

Head: Feebly vested by light, recumbent setae; surface weakly punctate; frons bi-impressed; eyes enlarged, bulging laterally, coarsely faceted; antennae extending to anterior third of elytra; third antennomere about twice the length of second antennomere; antennomeres 3–10 about the same length; antennomeres 4–10 somewhat robust, slightly serrate; eleventh antennomere robust, subacuminate.

Thorax: Pronotum scarcely punctate; faintly rugose laterally, smooth; vested by semierect seta interspersed with fine, recumbent setae; broadest at middle; disc flat, very feebly impressed in front of middle, more strongly constricted behind middle, subbasal tumescence absent. Mesoventrite very finely vested, smooth. Metaventrite smooth, convex, puncticulate; covered with fine, semi-recumbent setae. Scutellum subquadrate, notched posteriorly.

Legs: Vested with short, recumbent setae intermixed with long, erect and semi-erect setae. Femora rugulose; finely punctate. Tibiae longitudinally rugose, punctate, vested with short, recumbent setae intermixed with semi-erect setae.

Elytra: Humeri rounded, indicated; sides subparallel, widest behind middle; base wider than pronotum; disc flattened apically; apices subtriangular, slightly dehiscent; disc convex; vestiture composed of stiff, erect and semi-erect setae intermixed with stiff, semirecumbent setae; sculpturing consisting of small, shallow punctations arranged in striae that gradually reduce in size on middle third and do not reach elytral apex; interstices smooth, 4.0× the width of punctuation at anterior margin.

Abdomen: Ventrites 1-4 rugulose, feebly vested with short, recumbent setae, indistinctly, finely punctate. First visible ventrite about the same length of second ventrite, ventrite 3-4 subquadrate, smooth, feebly vested with fine recumbent setae. Fifth visible ventrite reduced, convex, lateral margins subparallel, posterior margin broadly, slightly emarginate. Sixth visible ventrite subquadrate, surface somewhat concave medially, convex laterally, feebly punctate; lateral margins oblique; posterior margin broadly, moderately deeply emarginate, emargination V-shaped, posterolateral angles rounded. Fifth tergite convex; finely punctate; rugulose; lateral margin subparallel; posterior margin broadly, shallowly, slightly, emarginate. Sixth tergite subtriangular; surface convex; longer than broad; finely punctate; scarcely covered with short, recumbent setae; lateral margins oblique; posterior margin narrowly, very shallowly emarginate; hind angles rounded. Posterior margin of sixth tergite produced ventrally, fully covering sixth visible ventrite.

Aedeagus: Phallobasic apodeme present; phallus with copulatory piece expanded at apex; phallic plate without denticles; intraspicular plate present, elongate; phallobasic apodeme long, expanded distally; phallobase trigonal; parameres free; tegmen complete, covering phallus; parameres pointed anteriorly; endophallic struts long, the length of tegmen; endophallic struts slender distally (Fig. 18B).

Sexual dimorphism: The female of *A. marginalis* can be separated from males based on the structure of the last abdominal segment. In females, the lateral and posterior margins of the sixth tergite and the sixth visible ventrite are broadly rounded, making a single semicircular margin; males have the sixth tergite and the sixth visible ventrite subquadrate in shape, and the posterior margin narrowly, shallowly emarginate, the emargination seen in the sixth visible ventrite is slightly deeper than that observed in the sixth tergite. Remaining characters are similar.

#### Material examined.

PARATYPE: 1 male: Pine Springs, TX, VII-12-16-1928, W. Benedict.

#### Additional material examined.

USA: 1 male: Hudspeth Co., TX, 9 mi SW Dell City, VII-31-1950, R. F. Smith; 2 males: Valentine, TX, VI-25-1947, R. H. Beawer. MEXICO: 1 male, 1 female: Sonora, Mexico, near San Jose beach, Ciudad Obregon, 40 mi SW of V-16-23-1961, Howden and Martin; 1 female: Coahuila, Mexico, sand dunes, near Bilbao, 8 mi N of Viesca, V-30-31-1981, J. Doyen and J. Liebherr.

### Araeodontia peninsularis

Taxon classificationAnimaliaColeopteraCleridae

(Schaeffer, 1904)

Figs 1C , 6E , 8A , 16A–D , 18C

#### Synonyms.

*Cymatodera peninsularis*. Schaeffer, 1904, Jour. New York Ent. Soc., vol. 12, p 214. Wolcott 1910, Field Museum of Natural History, zool. Ser., vol. 7, no. 10, p. 34; 1921, Proc. U.S. Natl. Mus., vol. 59, p. 286. Chapin 1949, Smithsonian Misc. Coll., vol. 111, no. 4, p. 9. Barr 1950b, Proc. California Acad. Of Sci., ser. 4, vol. 24, no. 12, p. 496.

#### Type material not examined.

#### Type locality.

Mexico, San Felipe, Baja California Sur, Cape region. Type depository: National Museum of Natural History (USNM).

#### Distribution.

USA: AZ, CA, NM; Mexico: Baja California, Sinaloa, Sonora.

#### Differential diagnosis.

*Araeodontia peninsularis* is most similar to *A. picta*. Differences in the size and position of the maculae on the elytral disc will help to distinguish these species. The anterior pair of testaceous maculae on the elytral disc of *A. peninsularis* reach the epipleural fold and these spots are more closely approximate to the anterior margin on the anterior half of the elytral disc (Fig. 1C). The elytral disc of *A. picta* possesses two maculae that do not reach the epipleural fold, and these spots are more distant from the anterior margin on the anterior half of the elytral disc (Fig. 1D). Additionally, antennomeres 3-10 on *A. peninsularis* are shorter in length than those found on *A. picta*.

#### Redescription.

Male. Form: Body somewhat slender, somewhat elongate. Color: Head, pronotum, thorax, abdomen, mouthparts and legs testaceous to light brown, elytra brown to dark brown; mandibles in lateral view brown with posterior half black; two irregular, testaceous maculae on each elytron, the first located on the anterior half, reaching middle third of elytral disc, and the second maculae adjacent to epipleural apex (Fig. 1C).

Head: Feebly vested by semi-erect setae; surface weakly punctate; frons bi-impressed; eyes large, bulging, coarsely faceted; antennae extending to anterior third of elytra; third antennomere about 1.5× the length of second antennomere; antennomeres 3–10 about the same length; antennomeres 4–10 robust; eleventh antennomere robust, subacuminate, slightly longer than tenth antennomere (Fig. 8A).

Thorax: Pronotum punctate; somewhat rugose laterally, disc smooth; vested by stiff semi-erect seta interspersed with fine, recumbent setae; broadest at middle; disc flat, moderately impressed in front of middle, more strongly constricted behind middle, subbasal tumescence absent. Mesoventrite very finely vested, smooth. Metaventrite smooth, convex, puncticulate, covered with fine, semi-recumbent and recumbent setae. Scutellum subquadrate, notched posteriorly.

Legs: Femora rugulose; finely punctate; vested with short, recumbent setae intermixed with long, semi-erect setae. Tibiae longitudinally rugose; rather punctate; vested with short, recumbent setae intermixed with semi-erect setae.

Elytra: Humeri indicated; sides subparallel, widest behind middle; base wider than pronotum; disc flattened apically; apices subtriangular, feebly dehiscent; disc convex, vestiture on elytral disc composed of stiff, abundant, semi-erect setae intermixed with less numerous, stiff, semi-recumbent setae and some erect setae scattered throughout elytral disc; sculpturing consisting of deep punctations arranged in regular striae that gradually reduce in size on posterior third and do not reach elytral apex; interstices smooth, 2.5 to 3.0× the width of punctuation at anterior margin.

Abdomen: Ventrites 1-4 rugulose, feebly vested with short, recumbent setae; indistinctly, finely punctate. First visible ventrite about twice the length of second ventrite; ventrite 2-4 subquadrate, short, smooth, feebly vested with fine recumbent setae. Fifth visible ventrite reduced, convex, lateral margins subparallel, posterior margin broadly, deeply emarginate. Sixth visible ventrite subquadrate, surface somewhat concave medially, convex laterally; slightly punctate, lateral margins oblique; posterior margin broadly, shallowly emarginate, emargination V-shaped, posterolateral angles rounded (Fig. 16B). Fifth tergite convex; finely punctate, rugulose, lateral margins subparallel, posterior margin broadly, shallowly, feebly, emarginate. Sixth tergite subtriangular; surface convex; longer than broad; finely punctate; scarcely vested with some short, recumbent setae; lateral margins oblique; posterior margin narrowly, very shallowly emarginate; hind angles rounded (Fig. 16A). Posterior margin of sixth tergite partially produced ventrally, fully covering sixth visible ventrite.

Aedeagus: Phallobasic apodeme present; phallus with copulatory piece rounded apically; phallic plate devoid of denticles; intraspicular plate present, somewhat elongate; phallobasic apodeme long, conspicuously expanded distally; phallobase trigonal; parameres free; tegmen complete, fully covering phallus; parameres pointed anteriorly; endophallic struts long, as long as the length of tegmen; endophallic struts slender distally (Fig. 18C).

Sexual dimorphism: Females of this species can be distinguished from males based on the structure of the last abdominal segment. Females have the lateral and posterior margins of the sixth tergite and the sixth visible ventrite broadly rounded, forming a single semicircular margin (Fig. 16C–D). Males have the sixth tergite and the sixth visible ventrite subquadrate in shape, and the posterior margin narrowly, shallowly emarginate, the emargination observed in the sixth visible ventrite is somewhat deeper than in the sixth tergite (Fig. 16A–B). Remaining characters are similar for both sexes.

#### Material examined.

2 females: Baboquivari Mts. AZ, Baboquivari Canyon, VII-17-1949, F. Werner and W. Nutting; 2 males, 3 females: Tucson, AZ, VIII-5-1935, Bryant; 1 male, 2 females: Hualpai Mts. AZ, VII-4-19, D. J. Knull and J. N. Knull; 1 male, 1 female: Tucson AZ, VII-12-19, Knull and J. N. Knull; 1 male: Santa Rita Mts., AZ, VII-13, [Compared with Type], Knull and J. N. Knull; 1 male, 1 female: Carlsbad, NM, VII-27, Knull and J. N. Knull; 1 male, 1 female: Globe, AZ., V-1939, D. K. Duncan; 1 male, 1 female: Globe, AZ, VII-20-1939, Parker; 2 males: Tucson, AZ, VIII-10-1939, Bryant; 2 females: Pima Co., AZ, Sabina Canyon, VII-17-1973, E. Giesbert; 1 male: Baboquivari Mts., AZ, sweeping slash, in desert, VII-31-1950, R. H. Arnett; 1 male, 3 females: Pima Co., AZ, 1 mi S of Kits Peak rd., IX-10-1974, J. M. Cicero; 1 female: Sta. Catalina Mts., AZ, Mouth of Bear Cn., VII-3-1961, Werner and Nutting; 2 males: foothills Sta. Catalina Mts., AZ, VII-2-1975, K. Stephan; 1 female: Riverside Co. CA, Palm Desert, V-15-1970, A. Mayor; 2 males: Riverside Co., CA, Deep Cyn. Des. Res. Center Sec. 17, R6E, T6S, 116°22'36"W, 33°36'19"N, 10-year Malaise trap study, VI-24-27-1980, J. D. Pinto and S. I. Frommer; 2 males, 3 females: Santa Cruz Co., AZ, Madera Cyn. 4880 ft., VII-23-1963, V. L. Vesterby; 1 female: Pima Co., AZ., Sabino Cyn., VI-25-1963, F. D. Parker and L. A. Stange; 2 males, 1 female: San Diego Co., CA, 6 mi E Banner, VII-13-1963, T. Bolton; 1 male: Baboquivari Mts. AZ., Baboquivari Cyn., VII-17-1949; 2 females: Mohave Co., AZ, Mohave Valley, VI-10-1980; 1 female: Globe, AZ, [September], D. K. Duncan; 2 males: Baboquivari Mts. AZ, Brown Cyn., VIII-4-1961, U. V. lt., W. Nutting; 1 female: Pima Co. AZ, IBP site, Sta. Rita Range Res., UV trap, VIII-31-1973, W. Nutting; 1 female: Pima Co., AZ, Sta. Rita Ranch, VII, R. Lenczy; 2 males: 3 females: Pima Co., AZ, Organ Pipe Natl. Mon., VI-14-1952, M. Cazier and R. Schrammel; 1 male, 2 females: Pima Co., AZ, 15 mi E. Tucson, 2600 ft., VIII-18-1950, T. Cohn, P. Boone and M. Cazier; 2 males: Hidalgo Co., NM, Cienega Ranch N Rodeo, VII-12-1948, C. Vaurie and P. Vaurie; 1 male, 2 females: San Carlos, AZ, VIII-13-1933, Parker; 2 males, 1 female: Globe, AZ, VIII-3-1933, Parker; 1 female: Coyote Mts. AZ., VIII-4-7-1916, 31°50'N 111°29'W 35000 ft., 2 males: Tucson, AZ, AC. 5409, Palm, no collector data; 1 male, 1 female: Baboguivari Mts., AZ, Near Kits Peak, VIII-7-9-1916, 32°00'N 111°36'W ~3600; 2 males: Globe, AZ, D. K. Duncan. MEXICO. 1 male, 2 females: Sonora, Mexico, Tastiota, VII-18-1952, C. Vaurie and P. Vaurie; 2 females: Sinaloa, Mexico, 16 miles SW Guamuchi, VI-16-1961, F. D. Parker.

### Araeodontia picipennis

Taxon classificationAnimaliaColeopteraCleridae

Barr, 1952a

#### Synonyms.

*Cymatodera picipennis* Barr, 1950b, Proc. California Acad. Sci., ser. 4, vol. 24, no. 12, p 495.

#### Type material not examined.

#### Type locality.

Venancio, Lower California. Type depository: California Academy of Sciences (CASC).

#### Distribution.

Mexico: Baja California.

The following is Barr’s (1950a) original description for *Cymatodera picipennis*.

Female: Medium size, somewhat elongate; piceous; pronotum faintly paler at sides and across middle; elytra with brownish subapical spots, right elytron with a broad, faintly indicated, brownish ante-median area along lateral margin at middle; undersurface dark testaceous. Head finely, somewhat sparsely punctured, finely wrinkled at base, sparsely clothed with short, erect brownish hairs; front feebly bi-impressed; antennae brown, stout, reaching basal fourth of elytra, second segment two-thirds as long as third, third segment slightly longer than fourth, segments 5 to 10 nearly equal in length, longer than those preceding, cylindrical, outer margin of each of these segments broadly rounded, slightly incrassate at apex. Pronotum one-third longer than basal width; surface finely, sparsely punctured, sparsely clothed with short, fine pale hairs, intermixed with moderately long, erect brown hairs; ante-scutellar impression wanting. Elytra two and one-half times longer than basal width, nearly twice as wide as pronotum at base; humeri distinct; sides widest behind middle; apices nearly conjointly rounded; surface with striae consisting of fine punctures, extending to subapical spots, interspaces much wider than punctures, sparsely clothed with short, suberect pale hairs. Legs dark testaceous, piceous at apices of femora and bases of tibiae, finely, densely punctured, densely clothed with short, brown hairs; middle tibiae dark. Metaventrite finely and very sparsely punctured. Abdomen finely, densely punctured; fifth sternite rounded at apex, deeply incised at middle; sixth sternite semicircular in shape; sixth tergite longer and broader than sixth sternite, narrowly rounded at apex. Length: 7 mm.

Holotype, female (C. A. S. No. 5622) from Venancio, July 17, 1938, collected by Michelbacher and Ross. *C. picipennis* belongs to the *Xanti* group in Wolcott’s key and will run to *C. tuta* Wolcott and *C. laevicollis* Schaeffer. It may be separated from these two species by the dark piceous color with the brown, subapical elytral spots and by the structure of the antennae. This species is described from a single female which is in a somewhat damaged condition, the left antenna is broken off at the fourth segment, one of the hind legs is missing, and several of the tarsi are gone. However, the critical characters are present and the species appears to be sufficiently distinct to warrant a name at this time.

#### Remarks.

Barr (1952a), in his revision of the genus *Araeodontia*, stated that this species is restricted to an area in the vicinity of San Venancio, Baja California Sur, Mexico, and it is only known from the female holotype. Barr indicated that *A. picipennis* is most similar to *A. peninsularis*; however, the two species can be differentiated by the structure of the tarsal claws and the elytral disc pattern; specifically, in *A. picipennis*, the two inner tarsal denticles are slender and closely approximated and the elytral disc is immaculate, in a pale testaceous tone. In *A. peninsularis*, the tarsal denticles are thicker and distinctly separated, and each elytron has two irregular testaceous maculae, the first located on the anterior half, reaching the middle third of elytral disc, and the second maculae adjacent to epipleural apex. Barr pointed out that the validity of the species is questionable and perhaps its rarity is due to its close resemblance with *A. peninsularis*, with the holotype possibly just a case of the maculae being absent. If so, *A. picipennis* would be treated as a junior synonym of *A. peninsularis*.

### Araeodontia picta

Taxon classificationAnimaliaColeopteraCleridae

Barr, 1952a

Fig. 1D

#### Paratypes.

 Two females examined.

#### Type locality.

Mexico, Chihuahua, Valle de Olivos. Type depository: American Museum of Natural History (AMNH).

#### Distribution.

Mexico: Chihuahua.

#### Differential diagnosis.

*Araeodontia picta* is most similar to *A. peninsularis*. The two species can be reliably separated based on the maculae on the elytral disc. The anterior pair of testaceous maculae of *A. picta* are well separated from the anterior margin of the elytral disc and do not reach the epipleural fold (Fig. 1D); these spots are noticeably closer to the anterior portion of the elytral disc in *A. peninsularis* and are in partial or total contact with the epipleural fold (Fig. 1C).

#### Redescription.

Female. Form: Body relatively slender, feebly elongate, similar in shape to remaining *Araeodontia* species. Color: Head, pronotum, thorax, abdomen, mouthparts and legs testaceous to light brown, elytra brown to dark brown; mandibles black; two irregular testaceous maculae on each elytron, the first located on middle of elytral disc, the second adjacent to epipleural apex (Fig. 1D).

Head: Feebly vested by semierect, stiff setae mixed with semi-recumbent fine setae; surface weakly punctate; frons slightly bi-impressed; eyes large, bulging, coarsely faceted; antennae extending slightly beyond elytral humeri; third antennomere about 2× the length of second antennomere; third antennomere shorter than fourth antennomere; antennomeres 4-10 somewhat robust, about the same length, feebly serrate; eleventh antennomere robust, acuminate, somewhat longer than previous antennomere.

Thorax: Pronotum punctate, more densely punctate than head; disc smooth; lateral sides rugulose; moderately vested with stiff, semi-erect seta interspersed with some fine, recumbent setae; broadest at middle; disc flat, inconspicuously impressed in front of middle, more strongly constricted behind middle, subbasal tumescence absent. Mesoventrite very finely vested, smooth, vestiture consisting of fine, semi-recumbent setae. Metaventrite smooth, convex, puncticulate, covered with fine, semi-recumbent and recumbent setae. Scutellum subquadrate, notched posteriorly.

Legs: Femora rugulose; finely punctate; vested with short, recumbent setae. Tibiae longitudinally rugose; more heavily punctate than femora; vestiture consisting of short, semi-recumbent setae intermixed with some semi-erect setae.

Elytra: Humeri indicated; sides subparallel, widest behind middle; base wider than pronotum; disc flattened apically; apices subtriangular, rather dehiscent; disc convex, vestiture on elytral disc consisting of stiff, semi-erect setae intermixed with numerous finer, semi-recumbent setae; sculpturing consisting of shallow punctations arranged in regular striae that gradually reduce in size on middle third and do not reach elytral apex; interstices smooth, about 4.0× the width of punctation at elytral base.

Abdomen: Ventrites 1-4 rugulose, feebly vested with short, recumbent setae; indistinctly, finely punctate. First visible ventrite about twice the length of second ventrite, ventrites 2-4 subquadrate, short, smooth, weakly vested with fine, recumbent setae. Fifth visible ventrite subtriangular, convex, lateral margins oblique, posterior margin truncate. Sixth visible ventrite rugulose, surface slightly concave, punctate, lateral and posterior margins broadly rounded. Fifth tergite convex, lateral margins subparallel, posterior margin truncate. Sixth tergite rugulose, surface feebly convex, broader than long, lateral and posterior margins broadly rounded. Posterior margin of sixth tergite slightly extending beyond posterior margin of sixth visible ventrite.

Aedeagus: Not available.

Sexual dimorphism of this species is provided in the original description given by Barr (1952a): [male] densely punctate; sternites 1-4 with a smooth, hind margins narrowly membranous; fifth [ventrite] shallowly compressed medially, lateral margins oblique, slightly arcuate, hind margins narrowly broadly, semicircularly emarginate; sixth [ventrite] broader than long, lateral margins nearly parallel, hind angles nearly square, broadly rounded, [posterior] margin more or less broadly arcuate, deeply, nearly semicircularly notched at middle; sixth tergite broader and longer than sixth [ventrite], slightly broader than long, disk feebly convex, lateral margins slightly oblique, hind margin nearly semicircular in shape, ventral surface with a very distinct, broad, transverse, subapical, V-shaped carina.

#### Material examined.

PARATYPE: 1 female: 20 mi SW Camargo Chihuahua, Mex., 4500 ft., VII-13-1947, Cazier. PARATYPE: 1 female: 63 miles W. of Santa Barbara Chihuahua, Mexico, 5500 ft., VII-20-1947, W. Gertsch and C. D. Michener.

#### Additional material examined.

2 females: 63 mi. W of Santa Barbara, Chihuahua, Mexico, 5500 ft., VII-02-1947, Michener.

#### Remarks.

Barr (1952a), in his revisionary work of *Araeodontia*, described *A. picta* as a new species endemic to the southern portion of Chihuahua, Mexico. In the type material revised by him, he indicated the existence of a single male, in this case, the holotype. Remaining specimens in the type series are females. The species is particularly uncommon in most collections; consequently, it was impossible to obtain males for this revisionary work. For that reason, the female paratype was redescribed.

### Barrotillus

Taxon classificationAnimaliaColeopteraCleridae

Rifkind, 1996

#### Type species.

*Barrotillus kropotkini* Rifkind, 1996, original designation (monotypy).

#### Distribution.

Shown in Fig. 21C.

### Barrotillus kropotkini

Taxon classificationAnimaliaColeopteraCleridae

Rifkind, 1996

Figs 1E , 7D , 8B

#### Paratype.

One male paratype examined.

#### Type locality.

Francisco Morazán, Tegucigalpa, Honduras. Type depository: Natural History Museum of Los Angeles (LACM).

#### Distribution.

Francisco Morazán, Honduras.

#### Differential diagnosis.

This monotypic species is most closely allied to members of *Neocallotillus*, with a particular resemblance to *Neocallotillus elegans*. A number of characters are useful to separate these species: *Barrotillus kropotkini* has the antennae composed of 11 antennomeres with the segments moderately serrate (Fig. 1E; 8B), the anterior portion of the pronotum is strongly constricted posteriorly, and the pronotal disc is coarsely and deeply punctate (Fig. 7D). *Neocallotillus elegans* has the antennae with 10 antennomere which are pectinate in males (Fig. 8D–E) and serrate in females (Fig. 8-F), the pronotal disc is somewhat constricted posteriorly (Fig. 2B), and the punctations on the elytral disc are shallowly and slightly impressed.

#### Redescription.

Male. Form: Body elongate, slender, small size, 5.3 mm. Color: Head, dorsal portion of pronotum, elytra, abdomen, legs and labial palpi piceous; ventral and posterior portion of pronotum, prosternum, mesoventrite, labrum, mouthparts and posterolateral portion of metaventrite rufous, antennae dark-brown; each elytron with one macula and one fascia, both markings white and raised from elytral surface, the macula is located on the median region of the anterior third of the elytral disc and the fascia is located on the median region of the elytral disc, the fascia begins on the epipleural fold and do not reach the elytral suture (Fig. 1E).

Head: Including eyes wider than pronotum; integument smooth, punctate; eyes large, finely faceted, anteriorly emarginate; frons not bi-impressed; clothed with semi-erect setae of two sizes; antennae reaching humeral angles, consisting of 11 antennomeres; antennomeres 2-4 small, slightly robust, filiform; antennomeres 5-6 feebly serrate; antennomeres 7-10 moderately serrate; antennomeres 5-10 gradually increasing in size; eleventh antennomere as long as the combined length of antennomeres 8-10; last antennomere rather compressed at middle (Fig. 8B); terminal labial palpi securiform; terminal maxillary palpi cylindrical.

Thorax: Pronotum longer than broad, campanulate; disc convex; sides sinuate; clothed with long, semi-erect setae intermixed with less numerous, long, semi-erect setae; widest on anterior margin; conspicuously constricted posteriorly; moderately punctate, punctations rather deep and coarse. Prosternum smooth, punctate, finely vested with pale, semirecumbent setae. Mesoventrite smooth, feebly punctate, coarsely deeply punctate, finely vested with some pale, semi-recumbent setae. Metaventrite slightly punctate, surface smooth, vested with fine, recumbent and semi-recumbent setae, longitudinal depression and metaventral process absent. Metepisternum visible throughout its length in lateral view.

Elytra: Humeri indicated, slender, elongate, subparallel, slightly broader on posterior third, convex on anterior third, then moderately compressed in middle third, and conspicuously convex again in posterior third, sinuosity observable on lateral view, sculpturing consisting on shallow punctations irregularly arranged, punctations extending to apex, elytral apices rounded, feebly dehiscent, interstices at elytral base about 2.5× the width of punctation; scutellum subquadrate, profusely vested with fine, recumbent and long setae, not compressed; epipleural fold compete, narrowing toward apex.

Legs: Femora shiny; smooth; vested with semi-recumbent setae interspersed with some semi-erect setae. Tibiae more profusely vested than femora; vestiture consisting on fine, short, recumbent setae on proximal face of tibiae and semi-erect setae on distal face of tibia. Pulvillar formula 4-3-3. Two tarsal denticles, tarsal denticles trigonal in shape.

Abdomen: Six visible ventrites. Ventrites 1-5 shiny, smooth, subquadrate, vested with fine, short, vested with semi-recumbent and recumbent setae; not compressed laterally. Fifth visible ventrite subtriangular; slightly clothed with recumbent setae; lateral margins oblique; posterior margin truncate. Sixth visible ventrite small, shiny, smooth, conspicuously broader than long; lateral margins strongly oblique; posterior margin broadly, shallowly emarginate; posterolateral angles rounded. Sixth tergite feebly concave; surface smooth; lateral margins strongly oblique; posterior margin notched medially; posterolateral angles semicircular in shape; lateral and posterior margins clothed with conspicuously long, erect setae. Sixth tergite extending beyond apical margin of sixth visible ventrite; fully covering sixth ventrite from dorsal view.

Aedeagus: Not available.

Sexual dimorphism: Rifkind (1996) indicated the presence of antennal differences between the male and the female of this species. The eleventh antennomere of the female is somewhat shorter than that of the male; also, in the female, this segment is not medially compressed. Additional differences between males and females are seen in the sixth sternite, where females have the posterior margin of this segment complete and rounded, rather than notched, as observed in males.

#### Material examined.

PARATYPE: 1 male: Honduras: Francisco Morazán, El Rincón, Tegucigalpa, X-5-1988, R. D. Cave.

#### Remarks.

Rifkind (1996) mentioned the close resemblance this genus has with many species of *Stenocylidrus* Spinola, checkered beetles restricted to the Afrotropical region and some Australasian islands. Rifkind further indicated that the campanulate state of *B. kropotkini* is similar to certain species of *Cladiscus* Chevrolat, *Pseudopallenis* Kuwert and *Eburneocladiscus* Pic, those genera occurring in the tropical regions of Africa and Madagascar. In the New World, *Barrotillus kropotkini* is most closely allied to *Neocallotillus*. The campanulate state of the pronotum of this species can be considered a homoplasy shared with many tillinids, rather than an indication of relatedness.

### Bogcia

Taxon classificationAnimaliaColeopteraCleridae

Barr, 1978

#### Type species.

*Bogcia disjuncta* Barr, 1978, original designation.

#### Distribution.

Shown in Fig. 21D.

### Bogcia disjuncta

Taxon classificationAnimaliaColeopteraCleridae

Barr, 1978

Figs 1F , 2A , 7A , 8C , 16E–F , 18D–E

#### Synonyms.

*Bogcia oaxacae* Barr, 1978, syn. n. Taxonomy of the New World Clerid Genus *Bogcia* from Mexico, The Pan-Pacific Entomologist, 54: 287–291.

#### Paratypes.

One male and one female examined.

#### Type locality.

Mazatlán, Sinaloa, Mexico. Type depository: California Academy of Science (CASC).

#### Distribution.

Mexico: Chiapas, Guerrero, Jalisco, Nayarit, Oaxaca, Sinaloa; Central America: Nicaragua*

#### Differential diagnosis.

*Bogcia disjuncta* (Figs 1F, 2A) most closely resembles *Cymatodera bogcioides* Burke (Fig. 4F). The two species can be readily distinguished based on differences in the structure of the protarsal claw and the antennae. *Bogcia disjuncta* has the protarsal denticle in close proximity to the protarsal claw (Fig. 7A) and the antennae is strongly serrate (Fig. 8C). *Cymatodera bogcioides* has the protarsal claw conspicuously separated from the protarsal denticle (Fig. 7B) and the antennae is moderately serrate.

#### Redescription.

Male. Form: Rather robust, moderately wider posteriorly, elongate. Color: Head, antennae, mouthparts, thorax legs, elytra and abdomen testaceous to brown; posterior half of mandibles black 2-3 irregularly fuscous. Each elytron with a broad, black to brown oblique fascia located behind median region of elytron with varying degrees of extension, ranging from the epipleural fold to the elytral suture, to two reduced, dark maculae, this fascia is preceded by a narrow, pale region; in addition to the dark fascia there is one small, brown to black humeral macula, this spot is absent in some specimens examined (Figs 1F, 2A).

Head: Measured across eyes wider than pronotum; surface rugose; frons feebly bi-impressed; coarsely punctate; eyes medium-sized, somewhat rounded, inconspicuously longer than wide, emarginate in front, bulging laterally, separated by approximately 2.5 eye-widths; antennomeres 2-3 very slightly serrate; third antennomere about 2× the length of second antennomere; fourth antennomere as long as third antennomere; antennomeres 4-10 strongly serrate, about the same length, as broad as long, posterior distal angle sharply pointed; eleventh antennomere about the same length as the tenth antennomere, with its distal margin moderately oblique (Fig. 8C).

Thorax: Pronotum rugose; widest behind middle; middle slightly wider than front margin; sides constricted subapically, more strongly constricted behind middle; disc flat, impressed in front of middle; subbasal tumescence somewhat pronounced. Prosternum smooth, slightly to moderately punctate. Mesoventrite rugulose, feebly to coarsely punctate. Scutellum subquadrate; wider than long; notched medially.

Legs: Femora shiny; finely transversally rugulose; indistinctly punctate. Tibiae coarsely, densely punctate; longitudinally rugose; clothed with long, erect setae and some short, recumbent setae.

Elytra: Anterior margin bisinuate, wider than pronotum; disc smooth, flattened above; humeri indicated; sides subparallel, widest behind middle; apices weakly dehiscent, triangular, covering sixth tergite; elytral declivity somewhat procurved, females slightly wider than males; sculpturing consisting of coarse punctations arranged in striae that gradually reduce in size behind middle; interstices smooth, about 2× the width of punctation.

Abdomen: Six visible ventrites. Ventrites 1-4 smooth; finely punctate; posterior margins truncate. First visible ventrite with a longitudinal carina that reaches the posterolateral angles (Fig. 7E); ventrites 3-4 slightly convex; hind margins truncate. Fifth visible ventrite convex; lateral margins oblique; posterior margin broadly, relatively deeply emarginate; hind angles narrowly rounded. Sixth visible ventrite subtriangular; rugulose; surface puncticulate, feebly convex; broader than long; lateral margins broadly oblique; posterior margin narrow, truncate; hind angles rounded (Fig. 16F). Fifth tergite rugulose; surface moderately convex; finely punctate; posterior margin shallowly emarginate. Sixth tergite broadly triangular; rugulose; surface very slightly convex; lateral margins strongly oblique, narrowing apically, producing a constricted, somewhat acuminate posterior margin (Fig. 16E). Sixth tergite extending beyond apical margin of sixth visible ventrite.

Aedeagus: Phallobasic apodeme present; phallus with copulatory piece tapered at apex; phallic plate unarmed, devoid of denticles; intraspicular plate present, elongate; phallobasic apodeme short, not expanded distally; phallobase subparallel; parameres free; tegmen incomplete, partially covering phallus; parameres pointed anteriorly; endophallic struts long; endophallic struts slender distally (Fig. 18D–E).

Sexual dimorphism: Females differ from males by having the posterior margin of the first and second visible ventrites truncate (Fig. 7E), and not acuminate (Fig. 7F) as observed in males. Additionally, females have the fifth visible ventrite rugose, with the lateral margins oblique and the posterior margin truncate; the sixth visible ventrite is rugulose, semicircular in shape, with the surface convex, and the lateral and posterior margins broadly rounded; the fifth tergite is rugulose, the lateral margins are oblique and the posterior margin is truncate; and the sixth tergite is rugulose, broader than long, with the surface inconspicuously convex, and the lateral and posterior margins strongly oblique and slightly acuminate posteriorly.

#### Material examined.

PARATYPES: 1 male, 1 female: 23 mi S of Matias Romero, Oaxaca, Mexico, 6-IV-1962, F. D. Parker and L. A. Stange.

#### Additional material examined.

2 males, 3 females: Jalisco, Mexico, Estacion de Biologia Chamela, VIII-21-1991, E. Ramirez; 3 females: Jalisco, Mexico, Estacion de Biologia Chamela, trampa de luz, VII-15-1986, R. A. Usela; 1 female: Jalisco, Mexico, Estacion de Biologia Chamela, trampa de luz, VII-13- 1986, R. A. Usela; 3 females: Jalisco, Mexico, Estacion de Biologia Chamela, trampa de luz, VII-9- 1986, R. A. Usela; 2 males, 1 female: Jalisco, Mexico, Estacion de Biologia Chamela, trampa de luz, VII-3- 1986, F. A. Noguera; 2 males, 3 females: Jalisco, Mexico, Estacion de Biologia Chamela, VII-15- 1986, R. A. Usela; 2 males: Jalisco, Mexico, Estacion de Biologia Chamela, atraido a la luz, VII-15- 1986, F. A. Noguera; 3 males, 1 female: Jalisco, Mexico, Chamela, VI-17- 1990, A la luz, F. A. Noguera; 2 males, 3 females: Jalisco, Mexico, Chamela, VI-15- 1990, A la luz, F. A. Noguera; 1 male: Mexico, Jalisco, Chamela, atraido a la luz, VII-7- 1986, F. A. Noguera; 2 males, 2 females: Jalisco, Mexico, Estacion de Biol. Chamela, VII-15-23-1987, F. T. Hovore, at UV and MV light; 3 males, 2 females: Jalisco, Mexico, Chamela, vic. UNAM, VII-9-19-1993, J. E. Wappes; 3 females: Jalisco, Mexico, vic. Estacion de Biologia Chamela, UNAM, VII-9-14-1993, Black light, Morris, Huether, Wappes; 2 males, 4 females: Jalisco, Mexico, Est. Biol. Chamela, VII-10-20-1985, E. Giesbert; 3 males, 2 females: Jalisco, Mexico, Chamela, vic. UNAM, VII-9-19-1993, J. Wappes; 2 males: Mexico, Jalisco, Estacion Biologica Chamela, VII-10-10-1985, E. Giesbert; 3 males, 1 female male: Jalisco, Mexico, Est. Biologica Chamela, VII-9-1981, Curoecol, trampa de luz, no collector data.

#### Remarks.

Interspecific variation in integument color and fasciae arrangement is a very common condition among numerous clerid species, and various descriptive works (Wolcott 1909, 1921, Rifkind 1993b, Leavengood 2008, Rifkind et al. 2010; Burke 2013, Burke and Zolnerowich 2014, 2015) have shown that abdominal and aedeagal differences are the most reliable morphological characters used for delineating interspecific boundaries within Cleridae. Barr (1978) described *Bogcia disjuncta* and *B. oaxacae* from the Pacific coast of Mexico, designating *B. disjuncta* as the type species. Differences in integument color and fascia pattern were the principal characters used by Barr to separate these species. The integument color and fasciae pattern from specimens examined here and identified as *B. disjuncta* and *B. oaxacae*, including one male and one female paratype of *B. oaxacae*, were highly variable and many intermediate forms were observed (Figs 1F, 2A). Additionally, the aedeagal and pygidial structures of these individuals were very similar and consistent (Fig. 18D–E). Consequently, the characters provided by Barr to separate these species are not sufficient to retain them as separate entities, and we designate *B. oaxacae* as a junior synonym of *B. disjuncta*.

### Bostrichoclerus

Taxon classificationAnimaliaColeopteraCleridae

Van Dyke, 1938

#### Type species.

*Bostrichoclerus bicornis* Van Dyke, 1938, original designation (monotypy).

#### Distribution.

Shown in Fig. 21E.

#### Type locality.

Palm Cañon, Angel de la Guardia, Golf of California, Mexico. Type depository: California Academy of Sciences (CASC).

#### Distribution.

USA: CA; Mexico: Baja California.

Due to the rarity of the species, this unusual clerid was not examined in this revisionary work; however, in order to complement the revision of the Tillinae in the New World, the descriptive work of Van Dyke (1938) is given here.

**Differential diagnosis.** The species is most similar to *Cymatodera*. It, however, does not look like any species of the latter genus, but at first sight rather like a large species of the genus *Polycaon* of the family Bostrichidae, also because of its size and general appearance somewhat suggests *Natalis* [Cleridae: Clerinae]. Its distinctive peculiarities are the prominent horns, the type of antennae and the glabrous elytra.

**Description.** Large, elongate, very finely and sparsely pilose. Head large; eyes large, transverse, coarsely granular, feebly emarginate in front, and very prominent; antennae long, 11 segmented, scape robust, segments 2-5 about twice as long as broad, feebly clavate and quite glabrous, a few stiff hairs only being evident, segments 6-10 moderately serrate, eleventh fusiform, the free angles of 6-8 densely clothed with fine silky pile and the three following segments completely clothed; a prominent horn, laterally compressed and bifid at apex, arising from in front of each eye and just within the insertion antennae giving the latter the appearance of arising from their base; mandibles robust; maxillary palpi four segmented, labial palpi three segmented, the terminal segments of both sets securiform, that of the labial palpi the larger, and almost an equilateral triangle. Prothorax robust, somewhat longer than broad, broadly constricted at sides in front of middle and narrowed posteriorly, basal margin a complete and well defined bead; coxal cavities rounded and narrowly opened behind. Elytra almost 3× as broad as prothorax, two and a half times as long as broad. Finely, densely and irregularly punctured and without striae except for fine sutural striae close to the suture and extending from about the middle almost to the apex. Anterior coxae conical, very narrowly separated, trochantine not visible; middle coxae somewhat conical well separated and with evident trochantine; hind coxae transverse. Abdomen with five free ventral segments. Legs long and slender; tibiae with short terminal spurs; tarsal segments all well developed, flattened dorsally, 1-4 broad yet longer than broad, with usual membranous appendages and densely papillose beneath, the fifth with sides somewhat papillose; claws simple.

***Bostrichoclerus bicornis*** Van Dyke, 1938.

**Description.** Holotype: unique from Palm Cañon, Angel de La Guardia Island, Gulf of California, collected May 3, 1921, by J. C. Chamberlin, from beneath bark. Moderately large, dark brown and somewhat shining. Head flattened in front, densely punctured above, smooth and sparsely punctured anteriorly, with a faint medial, longitudinal impression on front and sparsely pilose. Prothorax about a sixth longer than broad, base lobed at middle and sinuate each side, apex broadly arcuate and overhanging, disk irregularly punctured, more closely and deeply so in front and with short, reclinate hairs widely scattered about, and broadly and feebly impressed at middle. Scutellum semicircular, densely punctured, rugose and concave. Elytra convex, with pronounced though well rounded humeri, sides almost parallel and disc somewhat dull as the result of the dense punctations and fine rugoseness. Beneath somewhat shining, densely punctured anteriorly and sparely behind. Legs with apices of tibiae beneath and undersurfaces of the tarsal segments from 1-4 densely clothed with short, silky, orange pile. Length 20 mm. with head flexed, breadth 6.5 mm.

#### Remarks.

*Bostrichoclerus* is remarkably different from other tillinid species in the New World. Van Dyke (1938) indicated that, based on the coarsely faceted structure of the eyes, the genus should be placed within Tillinae. He thought *Bostrichoclerus* was closely related to *Cymatodera*, but *Bostrichoclerus* is very different from all known forms of *Cymatodera*. According to Van Dyke, *Bostrichoclerus bicornis* is easily identified based on the prominent frontal horns, the shape of the antennae, and the completely glabrous elytral disc. *Bostrichoclerus bicornis* was described based on single specimen collected in Isla Angel de la Guardia in the Golf of California, Baja California, Mexico. Later on, a second specimen was collected in southern California (Barr 1957). Material of this species has not been collected since.

### Cylidrus

Taxon classificationAnimaliaColeopteraCleridae

Latreille, 1829

#### Type species.

*Clerus cyaneus* Fabricius, 1787, original designation.

#### Synonyms.

*Epiteles* Newman, 1842. List of insects collected at Port Philipp, South Australia, by Edmund Thomas Higgins. Esq. (continued): Entomologist, vol. 1, pp. 361–369; 401–405 (1842).

#### Differential diagnosis.

Member of *Cylidrus* can be separated from remaining New World Tillinae species by the structure of the head (Figs 3A, 5D) and antennae (Fig. 8G). No other tilline in the New World has the head subquadrate in shape and conspicuously enlarged, the frons as wide as the total pronotal width, and the antennomeres 5-11 or 6-11 expanded and compressed laterally (Fig. 8G).

#### Redescription.

Size: 5–15 mm. Color: Light testaceous to black, fasciae on elytral disc absent or present, if present of various sizes and shapes. Body: Subparallel, elongate, robust to slender.

Head: Large, subquadrate in shape; including eye width wider than pronotum; integument shiny, somewhat punctate to smooth; eyes small, ovoid in shape, feebly faceted, weakly emarginate anteriorly, not bulging laterally; antennae with 11 antennomeres; antennomeres 5-11 to 6-11 expanded, compressed laterally; frons conspicuously wide, much wider than eye width, bi-impressed or not; terminal labial palpi subsecuriform; terminal maxillary palpi cylindrical, compressed laterally.

Thorax: Pronotum smooth to feebly punctate, inconspicuously widest at middle, sides subparallel. Prosternum smooth to slightly punctate. Mesoventrite moderately punctate. Metaventrite smooth, glabrous to conspicuously vested; metaventral process impressed to almost absent. Metepisternum concealed throughout its length in lateral view.

Elytra: Elongate; subparallel; inconspicuously widest behind middle; surface shiny to feebly punctate, punctations may extend to posterior third but never reach apex; scutellum ovoid, not compressed; vested to devoid of vestitures; epipleural fold complete, narrowing toward apex.

Legs: Feebly rugose; profemora not swollen; tibial spur formula 4-4-4; pulvillar formula 4-4-4; pulvilli weakly developed; two tarsal denticles; tarsal denticles digitiform in shape; moderately vested.

Abdomen: Six visible ventrites. Ventrites 1-5 impressed laterally or not; pygidium of males slightly differentiated from that of females; males with sixth ventrite emarginate or not; sixth ventrite of females simple, broadly rounded to rather subquadrate (Fig. 16D). Male and female pygidium shape similar.

### Cylidrus abdominalis

Taxon classificationAnimaliaColeopteraCleridae

Klug, 1842

Fig. 3A

#### Synonyms.

Cylidrus fasciatus var. spinolai Schenkling, Clerites II, 1910, p. 122.

#### Type locality.

Santa Catarina, Brazil. Type depository: Germany, Berlin, Museum für Naturkunde der Humboldt-Universität (ZMHB).

#### Distribution.

States of Espirito Santo, Mato Grosso do Sul, and Santa Catarina, Brazil.

#### Differential diagnosis.

*Cylidrus abdominalis* is most similar to *Cylidrus fasciatus* Laporte, a species inhabiting central and southern Africa. Gorham (1876) indicated that *C. fasciatus* was introduced to South America and eventually became adapted to this new habitat. The five Brazilian specimens of *C. abdominalis* examined here do not differ from *C. fasciatus*, a finding contrary to Gorham’s observations on elytral fasciae differences between these two entities. The fasciae observed in individuals examined under the name *C. fasciatus* display slight differences in shape, color, and pattern (Figs 3A, 5D). These fasciae can range from dark testaceous to almost albus, and can extend from the elytral suture to the epipleural fold, to only a pair of spots on the median region of the elytral disc. Specimens of *C. abdominalis* examined here are consistent with this variation. Remaining characters were not variable for material of both species.

#### Redescription.

Female. Form: Body elongate, slender, elytra subparallel. Color: Head, thorax, elytral fuscus; legs, mouthparts and abdomen testaceous; antennomeres 1-5 dark testaceous, antennomeres 6-11 fuscus. Each elytron with a median, transversal, testaceous fascia, this fascia initiates at the elytral suture and does not reach the epipleural fold (Fig. 3A).

Head: Longer than wide; enlarged throughout its length; including eyes wider than pronotum; eyes small, taller than wide, not bulging laterally, finely faceted, feebly emarginate posteriorly; antennal notch located in front of eye emargination; frons not bi-impressed; clypeus crenulate posteriorly; gena carinate, encircling eyes; submentum rugose, somewhat shiny; gular sutures parallel, slightly marked; integument punctate, rugose, more strongly rugose below eyes punctations fine and shallow, clothed with fine, pale, short, recumbent setae; antennae composed of 11 antennomeres; first antennomere slender; second antennomere slightly shorter than first antennomere; third antennomer somewhat longer than second antennomere; fourth antennomere about the same length as second antennomere; fifth antennomere about the same length as fourth antennomere; sixth antennomere about the same length as fifth antennomeres; antennomeres 6-10 about the same length, clavate; eleventh antennomere slightly longer than tenth antennomere, elongate, robust, obtusely rounded (8G); terminal labial palpi subsecuriform, terminal maxillary palpi, slender, cylindrical.

Thorax: Lateral margins of pronotum parallel, sides very feebly narrowing apically, strongly compressed in behind anterior margin; surface shiny, rugose, clothed with some fine, short, pale, semierect setae and some long, pale erect setae, vestiture more abundant laterally; very finely, scarcely punctate, punctations small and shallow. Prosternum convex, wider than long, smooth, polished, very feebly punctate, punctations small and shallow. Mesoventrite as long as wide, concave; strongly rugose; slightly vested with fine, pale, semi-erect setae; scarcely punctate, punctations coarse and deep. Metaventrite strongly convex; surface finely rugose, inconspicuously vested with fine, pale, semirecumbent setae; longitudinal depression and metaventral process absent. Metepisternum exposed throughout its length. Scutellum ovoid, compressed medially, glabrous.

Elytra: Slightly broader than pronotum; sinuate in lateral view; somewhat elongate; humeri feebly indicated, rounded; sides parallel, broader at middle; disc flat above; surface shiny, smooth; apices subtriangular, dehiscent; elytral declivity gradual, integument clothed with fine, short, dark, semirecumbent setae interspersed with very few scattered, long, semierect setae; sculpturing consisting of fine and shallow punctations irregularly arranged throughout elytral length; punctations at elytral base absent; epipleural fold narrow, gradually reducing toward distal end, absent on posterior fourth. Last two abdominal segments fully visible in dorsal view.

Legs: Femora shiny, smooth; slightly punctate; swollen; compressed laterally; clothed with some pale, fine, semirecumbent and semi-erect setae uniformly located throughout femoral integument. Tibiae somewhat slender; slightly broadening toward distal end; rather punctate; longitudinally rugose; vestiture consisting of pale, semirecumbent setae intermixed with some semierect setae.

Abdomen: Six visible ventrites. First visible ventrite longer than second visible ventrite. Ventrites 1-4 subquadrate, shiny, smooth, convex, finely punctate, clothed with fine, long, pale, recumbent setae; posterior margins truncate. Fifth visible ventrite subquadrate; integument convex, shiny, smooth; weakly clothed with fine, long, recumbent setae; lateral margins parallel; posterior margin broadly, shallowly, V-shaped emarginate. Sixth visible ventrite subquadrate, smooth, shiny, convex, almost flat; inconspicuously punctate; clothed with some erect and semierect, long, piceous setae; vestiture more abundant on anterolateral margins; lateral margins feebly oblique; posterior margin broadly rounded to almost truncate. Fifth tergite subquadrate; surface concave, rugulose, glabrous, punctate; lateral margins subparallel; posterior margin truncate. Sixth tergite subquadrate, slightly rugulose, longer than wide; posterolateral margins conspicuously vested with long and short erect setae; posterior margin more strongly vested; integument moderately, minutely punctate; lateral margins slightly oblique; posterior margin slightly rounded to almost truncate. Sixth tergite extending slightly beyond apical margin of sixth visible ventrite, fully covering sixth ventrite in dorsal view.

Aedeagus: Not available.

Sexual dimorphism: No males were available for examination.

#### Material examined.

1 female: Espirito Santo, [Brazil], Schmidt, 100 m, 1905; 2 females: Mato Grosso do Sul, Brasil, Selvíria, UNESP Farm, ex *Hevea brasiliensis* bole, VII-10-1990, S. R. Rodrigues; 1 female: Nova Teutonia, Santa Catarina, Brazil, VIII-7-1944, F. Plaumann; 1 female: Brazil, Nova Teutonia, IX-1973, F. Plaumann.

#### Remarks.

*Cylidrus* Latreille is composed of 19 species and seven subspecies distributed in the tropical regions of Africa and Oceania (Corporaal 1950). Gorham (1876) indicated that *Cylidrus abdominalis* is most similar to the African *C. fasciatus* and was probably transported from the Old World and became established in Brazil. *Cylidrus abdominalis* is here redescribed from material collected in the southeastern Brazilian provinces of Espirito Santo, Mato Grosso do Sul and Santa Catarina. Irrespective of its origin, whether a natural occurrence or an introduced species, the material examined here confirms the existence of this genus in the New World.

### Cymatoderella

Taxon classificationAnimaliaColeopteraCleridae

Barr, 1962

#### Type species.

*Tillus collaris* Spinola, 1844, original designation.

#### Distribution.

Shown in Fig. 21H.

#### Differential diagnosis.

*Cymatoderella* is most similar to various *Cymatodera* species of moderate dimensions. The two genera can be recognized based on the size of the ommatidia. *Cymatoderella* species have the diameter of the ommatidia somewhat small (Fig. 6F) compared to *Cymatodera* species (Fig. 12A). Additionally, the bicolored composition of the integument in *Cymatoderella*, with a testaceous to ferrugineous coloration on the head and pronotum and a piceous tone on the rest of the body (Fig. 3B–D) will serve to separate these genera. *Cymatodera bicolor* (Say) is the only species in the genus with a similar color pattern, but it has an elongate and narrow body shape (Fig. 5E), not a robust one, as observed in *Cymatoderella* (Fig. 3B–D).

#### Redescription.

Size: 3–7 mm. Body: Small, relatively robust individuals. Color: Pronotum bicolored, testaceous to ferruginous in the median region and piceous on the margins to uniformly testaceous to ferruginous; legs, antennae, thorax, elytra and abdominal segments piceous; head and mouthparts can be testaceous, ferruginous, or with an array of piceous tones; for *C. patagoniae*, visible ventrites 4-6 can be testaceous to ferruginous. Form: Small sized individuals, body short, robust, elytra subparallel to moderately expanded posteriorly.

Head: Eyes medium sized, taller than wide, bulging laterally, emarginate (Fig. 6F); sculpturing variously impressed; vestiture variable; antennal insertion located in front of emargination; clypeus emarginate medially; antennae with 11 antennomeres; sexual dimorphism slightly difficult to observe in the last abdominal segment; terminal maxillary palpi cylindrical; terminal labial palpi securiform (Fig. 3B–D).

Thorax: Pronotum narrower than elytral base; widest at middle; sides constricted subapically; more strongly constricted behind middle; disc convex; anterior depression feebly indicated; antescutelar impression absent; posterior margin conspicuously constricted transversally. Prosternum smooth, variously punctate and vested. Mesoventrite wider than long; shiny, variously punctate. Metaventrite convex, smooth, shiny, moderately clothed.

Legs: Femora swollen; tibia slender; rugose to rugulose; tibial spur formula 2-2-2, pulvillar formula 4-4-4.

Elytra: Broad; robust; gradually expanded behind middle; humeri strongly indicated; elongate; surface convex, expanded behind middle; moderately to coarsely sculptured; sculpturing arranged in regular striae; elytral declivity somewhat steep; epipleural fold complete, narrowing toward apex; pygidium concealed in dorsal view.

Abdomen: Six visible ventrites. First visible segment shiny; smooth; 1.5× longer than remaining segments. Ventrites 2-4 subquadrate; smooth; shiny; variously impressed and clothed; lateral margins parallel; posterior margins truncate. Fifth visible ventrite subquadrate; variously vested; lateral margins oblique; posterior margin truncate. Sixth visible ventrite subtriangular, displaying a degree of sexual dimorphism; lateral margins strongly oblique; posterior margin rounded to moderately emarginate. Fifth tergite subquadrate; posterior margin truncate. Sixth ventrite subtriangular.

#### Remarks.

*Cymatoderella* was established by Barr (1962) to separate *Tillus collaris* Spinola and *T. patagoniae* Knull, two New World species, from *Tillus* (Olivier), a widely distributed genus with a concentration of species in Africa and Oceania. Later on, Rifkind (1993a) described a third species, *Cymatoderella morula. Cymatoderella collaris* is widely distributed throughout North and Central America; the species ranges from the eastern and southern United States, extending southward to Mexico and the Central American countries of Guatemala, Honduras and El Salvador. *Cymatoderella morula* and *C. patagoniae* are species with a limited distributional range. *Cymatoderella morula* inhabits regions of southern Mexico, Guatemala, Honduras, and Nicaragua (Rifkind 1993a), and *C. patagoniae* is found in southern Arizona and Guerrero, Michoacan and Sonora, Mexico.

#### Key to species of *Cymatoderella* Barr

**Table:** 

1	Last abdominal segment ferruginous, remaining abdominal segments testaceous to almost piceous; elytral disc conspicuously clothed with pale, short, semirecumbent setae (Fig. 3D); elytra robust; Arizona, USA, Sonora, Jalisco and Guerrero, Mexico	***Cymatoderella patagoniae***
–	Last abdominal segment piceous to dark testaceous, the same color as remaining segments; distribution more widespread	**2**
2(1)	Antennomeres 2-4 subequal in length, short, cylindrical; antennomeres 5–10 robust, moderately serrate (Fig. 9D); widely distributed, from eastern and southern USA south to Nicaragua	***Cymatoderella collaris***
–	Antennomeres 2-3 subequal in length, short, cylindrical; antennomeres 4-10 robust, moderately serrate (Fig. 9E); distribution from southern Mexico to Guatemala and Honduras	***Cymatoderella morula***

### Cymatoderella collaris

Taxon classificationAnimaliaColeopteraCleridae

(Spinola, 1844)

Figs 3B , 6F , 9D , 19B

#### Synonyms.

*Tillus collaris* Spinola, 1844. Clérites I, Lec. Ann. Lyc. Nat. Hist. New York V, 1849.

#### Type material not examined.

#### Type locality.

l’Amérique Septentrionale. Type depository: Italy, Torino, Museo Regionale di Scienze Naturali (MRSN).

#### Distribution.

USA: AL, FL, GA, KY, LA, MD, MS, OH, SC, TN, TX; Mexico: Chiapas, Estado de Mexico, Jalisco, Nayarit, Nuevo Leon, San Luis Potosi, Sinaloa, Tamaulipas, Veracruz.

#### Differential diagnosis.

*Cymatoderella collaris* is most similar to *C. morula*. The two species can be differentiated based on the structure of the antennae. Antennomeres 2-4 of *C. collaris* are short, cylindrical and subequal in length, and antennomeres 5-10 are elongate, robust and moderately serrate (Fig. 9D). In contrast, *Cymatoderella morula* has antennomeres 2-3 short, cylindrical and subequal in length, and antennomeres 4-10 elongate, robust and moderately serrate (Fig. 9E). The geographic distribution of these species may also serve to differentiate them; *C. collaris* is widely distributed from the eastern and southern USA south to El Salvador, while *C. morula* is found in southwest Mexico, Guatemala, Honduras and Nicaragua.

#### Redescription.

Male. Form: Body short, robust, elytra gradually expanded toward apex, then abruptly narrowing behind distal fourth. Color: Pronotum uniformly testaceous to ferruginous throughout its surface to bicolored, if bicolored, ranging from testaceous to ferruginous in the median region and piceous on the margins; legs, antennae, thorax, elytra piceous; abdomen piceous to dark testaceous; head and mouthparts with various of piceous tones. Elytral disc devoid of any bands or fasciae (Fig. 3B).

Head: Including eyes wider than pronotum; eyes of moderate size, taller than wide, conspicuously bulging laterally, finely faceted, emarginate posteriorly; antennal notch located in front of emargination; frons impressed; integument shiny, punctate, punctations coarse; sparsely clothed with fine, pale, semirecumbent and semi-erect setae; antennae composed of 11 antennomeres; antennomeres 2-4 short, robust, subequal in length; fourth antennomere about 2.5× the length of fifth antennomere; antennomeres 5-10 robust, slightly serrate, approximately the same size; last antennomere elongate, robust, obtusely rounded, slightly longer than tenth antennomere (Fig. 9D); terminal labial palpi securiform; terminal maxillary palpi cylindrical, compressed distally.

Thorax: Pronotum bisinuate, widest at middle; sides constricted subapically, more strongly constricted behind middle, slightly constricted in front of middle; surface shiny, smooth, vested with fine, long pale, semirecumbent setae intermixed with some long semierect, fine, pale setae; in some individual vestiture is more abundant on the posterolateral area of the pronotum; finely punctate; punctations small and shallow; anterior transverse depression and subbasal tumescence absent, abruptly compressed on posterior margin. Prosternum conspicuously wider than long; smooth; polished, devoid of punctation in most individuals, some specimens very feebly punctate, punctations coarse and shallow; vested with fine, pale, semi-erect setae. Mesoventrite shiny, smooth, vested with fine, pale, semi-erect setae. Metaventrite strongly convex, surface shiny to finely rugulose, inconspicuously vested with fine, pale, recumbent setae; longitudinal depression and metaventral process present. Metepisternum hidden throughout its length. Scutellum ovoid, compressed medially, clothed with pale, fine, recumbent setae to glabrous.

Elytra: Broader than pronotum, elongate; broader than long; humeri indicated, rounded; sides subparallel, gradually broadening toward distal end, broadest behind middle, then abruptly narrowing toward apex on posterior fourth; surface shiny, rugulose; apices subtriangular; inconspicuously dehiscent; elytral declivity moderately steep; surface clothed with fine, short, recumbent, pale setae interspersed with some pale, fine, long, semi-erect setae; surface strongly, coarsely punctate; sculpture consisting of coarse, deep punctations arranged in regular striae that gradually reduce in size toward elytral apex, disappearing on posterior fourth; interstices at elytral base about 3 to 4× the width of punctation; interstices shiny to moderately rugulose.

Legs: Femora shiny, smooth; punctate, posterior and middle femora swollen, anterior femora more swollen; clothed with some pale, fine, semirecumbent and semi-erect setae uniformly located throughout the femoral surface; tibiae slender, punctate, longitudinally rugose, vestiture consisting of pale, recumbent setae interspersed with semi-erect setae.

Abdomen: Six visible ventrites. Ventrites 1-4 shiny; smooth; convex; subquadrate; punctate; vested with fine, long, pale, recumbent setae; not compressed laterally; posterior margins truncate. Fifth visible ventrite subtriangular; shiny; smooth; polished; surface convex; weakly clothed with fine, long, recumbent setae; lateral margins strongly oblique, arcuate; posterior margin broadly, shallowly emarginate to almost truncate in some individuals. Sixth visible ventrite small, rugulose; feebly convex; moderately, finely punctate; clothed with some erect and semierect, long, piceous setae; conspicuously broader than long; lateral margins strongly oblique; posterior margin broadly, shallowly emarginate to almost truncate; posterolateral angles broadly rounded. Fifth tergite subquadrate, convex; rugulose; glabrous; slightly punctate; posterior margin truncate. Sixth tergite subtriangular; rugulose; wider than long; convex; clothed with fine, pale, recumbent setae; integument punctate; lateral margins oblique, posterior margin truncate to rounded; posterolateral angles moderately to strongly rounded; some long, erect, pale, stout setae located along the posterior margin. Sixth tergite extending beyond apical margin of sixth visible ventrite, fully covering sixth ventrite in dorsal view.

Aedeagus: Phallobasic apodeme present; phallus with copulatory piece tapered at apex; phallic plate unarmed, denticles absent; intraspicular plate present, elongate, rounded; phallobasic apodeme short, expanded distally; phallobase subparallel; parameres free; tegmen incomplete, partially covering phallus; parameres pointed distally; endophallic struts long, as long as the length of tegmen; endophallic struts in horizontal position in relation to tegmen when in horizontal view; endophallic struts robust distally (Fig. 19B).

Sexual dimorphism: Females of *C. collaris* can be distinguished from males based on the shape of the last abdominal segment. Females have the sixth visible ventrite conspicuously long and broad, appearing as a semicircle, rather than short, subtriangular in shape, and broadly and shallowly emarginate posteriorly, as observed in males.

#### Material examined.

USA: 5 males, 9 females: Hidalgo Co., TX, III-26-1953, D. J. and J. N. Knull; 2 females: Hidalgo Co., TX, III-26-1956, D. J. and J. N. Knull; 1 male, 1 female: Hidalgo Co., TX, 07-IV-1961, D. J. and J. N. Knull; 3 females: Hidalgo Co., TX, IV-03-1961, D. J. and J. N. Knull; 1 male, 3 females: Mobile, AL, V-20-1922, H. P. Loding; 2 males, 4 females: Starr Co., TX, III-20-1952, D. J. and J. N. Knull; 1 male: Spring Hills, AL, 10-V-1918, H. P. Loding; 1 female: Gadsden Co., FL, V-8-1939, D. J. and J. N. Knull; 1 female: TX, Apple Springs, 13-V-1974, R. Reeve; 1 male: Jefferson Co., AL, Birmingham, 5-VII-1956, H. R. Steeves Jr., at light; 1 female: Ft. Mount, GA, IX-7-1937, P. W. Fatting; 2 female: Hidalgo Co., TX, 07-V-1957, D. J. and J. N. Knull; 1 males: Morehead, KY, 21-VI-1949, D. J. and J. N. Knull; 1 male, 2 females: Great Smoky Mountains Nat. Park, TN, VI-14-1942, D. J. and J. N. Knull; 2 males: Hidalgo Co., TX, III-29-1953, D. J. and J. N. Knull; 6 males, 1 female: Starr Co., TX, III-28-1950, D. J. and J. N. Knull; 1 male: Stone Mt., GA, VI-17-1949; P. W. Fattig; 1 male: Jefferson Co., AL, Vestavia, VII-18-1981, T. King, at light; 1 female: Starr Co., TX, III-31-1963, D. J. and J. N. Knull; 1 male, 2 females: Jefferson Co., AL, Birmingham, Shades Mts., VI-15-1982, T. King, at light; 1 male: Walker Co., AL, Jasper, X-09-1978, T. King, at light; 1 female: Walker Co., AL, nr. Jasper, Devil’s Ladder, 04-VII-1981, T. King, at light; 2 females: Clinch Co., GA, N of Homerville at Atkins Co. line, V-28-2004; beating in cypress bog, P. Skelley; 1 male: Liberty Co., FL, Torreya State Park, VII-17-1987, Matthews and Skelley, at light; 1 male: Dixie Co., FL, 3.5 mi N of Old Town, RT. 349, IV-27-1980, M. C. Thomas; 1 male: Alachua Co., FL, Hwy. 241 at Santa Fe River, IV-5-1989, C. W. Mills III, on bark on *Carya illionensis*; 2 females: Starr Co., TX, IV-5-1963, D. J. and J. N. Knull; 1 male: Liberty Co., FL, Torreya State Park, V-6-1989, R. Turnbow; 2 males, 1 female: Bexar Co., TX, Leon Valley, VI-14-1971, G. H. Nelson, beating *Diospytos texana*. MEXICO: 2 females: Mexico, San Luis Potosi, 41 mi N of San Luis Potosi, 26-VI-1965, G. H. and D. E. Nelson; 1 male, 1 female: Mexico, San Luis Potosi, 25.7 km W of Rio Verde, 4100’, 2-VI-1987; 1 male: Chiapas, Mexico, La Sepultura, V-2-2008, A. Burke; 2 males: Veracruz, Mexico, 2 km S Jalapa, VII-1985, J. Peña; 1 male: Jalisco, Mexico, Mismaloya River, 5 km E of Hwy. 200, VI-8-1991, W. B. Warner; 1 female: Estado de Mexico, Mexico, Temascaltepec, Bejucos, VII-1993, H. E. Hinton, R. L. Usinger; 1 male, 1 female: Nayarit, 3 mi NW Santa Maria del Oro, June 27, 1963, J. Doyen. 2 males, 1 female: Nuevo Leon, Mexico, 28 km SW Linares, VIII-12-2009, A. Burke, D. Cibrian.

#### Remarks.

After examination of material of *Cymatoderella collaris*, we observed that the morphology of this species is generally consistent throughout its geographical range. Certain characters, however, may vary in accordance to the collecting locality; this variation is apparent when comparing material collected in southeastern USA and the Florida peninsula with specimens collected elsewhere. Such variation is observable in the integument color of the abdomen of both sexes. Material collected in the southeastern USA and the Florida peninsula have the abdomen uniformly piceous; while those individuals collected in the mid-southern USA, Mexico and Central America have the abdomen moderately fuscous to dark testaceous. Remaining characters were constant for all individuals studied.

### Cymatoderella morula

Taxon classificationAnimaliaColeopteraCleridae

Rifkind, 1993a

Figs 3C , 9E , 19C

#### Paratypes.

Two males examined.

#### Type locality.

Mexico, Oaxaca, Sierra de Miahuatlán. Type depository: California Academy of Science (CASC).

#### Distribution.

Mexico: Oaxaca; Central America: Guatemala, Honduras.

#### Differential diagnosis.

*Cymatoderella morula* is most similar to *C. collaris*. Characters to distinguish these species appear in the diagnosis section of *C. collaris*.

#### Redescription.

Male. Form: Small and robust individuals, elytra gradually expanded toward apex, then abruptly narrowing behind distal fourth. Color: Pronotum uniformly testaceous to ferruginous throughout its surface to bicolored, if bicolored, can range from testaceous to ferruginous in the median region and piceous on the margins; legs, thorax and elytra piceous; abdomens dark testaceous; antennae uniformly piceous, or with scape and pedicel dark testaceous to piceous and remaining antennomeres piceous; head and mouthparts with various tones of piceous to brown tones. Elytral disc devoid of any bands or fasciae (Fig. 3C).

Head: Including eyes slightly wider than pronotum; eyes of moderate size, taller than wide, conspicuously bulging laterally, finely faceted, posteriorly emarginate; antennal notch located in front of emargination; frons bi-impressed; integument shiny, smooth, finely, sparsely punctate, punctations small, shallow; clothed with fine, pale, semirecumbent setae interspersed with some semi-erect setae; antennae composed of 11 antennomeres; antennomeres 2-3 short, robust, subequal in length; third antennomere about 2.5× the length of fourth antennomere; antennomeres 4-10 robust, moderately serrate, subequal in length; last antennomere elongate, robust, obtusely rounded, slightly longer than tenth antennomere (Fig. 9E).

Thorax: Pronotum bisinuate, widest at middle; sides constricted subapically, more strongly constricted behind middle, moderately constricted in front of middle; surface shiny, rugulose; vested with fine, short, pale, recumbent setae intermixed with some long and very long, erect, fine, pale setae, the latter setae located on the lateral margins of the pronotum; finely to moderately punctate; punctations small and shallow; anterior transverse depression and subbasal tumescence absent, compressed on posterior margin. Prosternum conspicuously wider than long; smooth; polished; carinate; devoid of punctation; glabrous. Mesoventrite shiny, smooth, vested with fine, pale, semi-erect setae; slightly punctate, punctations coarse and deep. Metaventrite strongly convex, surface shiny, smooth, inconspicuously vested with fine, pale, recumbent setae; longitudinal depression and metaventral process present. Metepisternum hidden throughout its length. Scutellum elongate, compressed medially, clothed with pale, fine, semirecumbent setae.

Elytra: Broader than pronotum; broader than long; humeri indicated, rounded; sides subparallel, gradually broadening toward distal end; broadest behind middle, then abruptly narrowing toward apex behind posterior third; disc flat above; surface shiny, smooth; elytral apices subtriangular; inconspicuously dehiscent; elytral declivity moderately steep; surface clothed with fine, short, pale, recumbent setae interspersed with some scattered pale, fine, long, erect setae; surface strongly, coarsely punctate; sculpturing consists of coarse, deep, punctations arranged in regular striae that gradually reduce in size toward elytral apex and completely disappear on posterior fifth; interstices at elytral base about 2× the width of punctation; interstices shiny, smooth.

Legs: Femora shiny, smooth, swollen, anterior femora conspicuously more swollen than middle and posterior femora; clothed with some pale, fine, semirecumbent and semi-erect setae; tibiae feebly rugulose, vestiture similar to that observed on femora, some specimens have tibiae more strongly vested than femora.

Abdomen: Six visible ventrites. Ventrites 1-4 shiny, convex, smooth, subquadrate, punctate, clothed with fine, long, pale, recumbent setae; not compressed laterally; posterior margins truncate. Fifth visible ventrite subtriangular; shiny; smooth; polished; surface moderately convex; clothed with fine, long, pale, recumbent setae; lateral margins strongly oblique, arcuate; posterior margin narrowly, shallowly emarginate. Sixth visible ventrite small, shiny, convex; finely punctate; sparsely clothed with some short, pale, fine, semi-erect setae; conspicuously broader than long; lateral margins strongly oblique, arcuate; posterior margin broadly, shallowly emarginate; posterolateral angles broadly rounded. Fifth tergite subquadrate, convex; glabrous; punctate; posterior margin truncate. Sixth tergite subtriangular; rugose; wider than long; convex; sparsely clothed with fine, pale, recumbent setae; integument moderately, finely punctate; lateral margins oblique, posterior margin rounded; posterolateral angles strongly rounded; some long, erect, dark, stout setae located along the posterior margin and posterolateral angles. Sixth tergite extending beyond apical margin of sixth visible ventrite, fully covering sixth ventrite in dorsal view.

Aedeagus: Phallobasic apodeme present; phallus with copulatory piece conspicuously swollen at apex; phallic plate unarmed, devoid of denticles; intraspicular plate present, short and rounded; phallobasic apodeme short, expanded distally; phallobase subparallel; parameres free; tegmen incomplete, partially covering phallus; parameres pointed distally; endophallic struts long, the length of tegmen; endophallic struts slender distally (Fig. 19C).

Sexual dimorphism: Females of *C. morula* can be distinguished from males based on the shape of the last abdominal segment. Females have the sixth visible ventrite broadly rounded posteriorly, rather than subtriangular in shape and broadly, shallowly emarginate, as observed in males.

#### Material examined.

PARATYPE: 1 male: Oaxaca, Mexico, Sierra de Miahuatlán, 5500’, Highway 175 10 km S of Miahuatlán, VII-5-1989, on dry oak forest, E. Barchert, A. Evans, J. Rifkind. PARATYPE: 1 male: Honduras, vic. L. Yojoa, Montaña de Pozo Azul, 28-V-1979, E. Giesbert.

#### Additional material examined.

1 male: departamento de Alta Verapaz, Guatemala, 57 km N of El Rancho, on new Cobán highway, V-30-1973, 1463 m, W. Opitz; 1 female: Granada, Nicaragua, Reserva Natural Volcan Mombacho, 11°50'04"N, 85°58'48"W 1100 m, V-19-2000, m.v. light. Smith, Ocampo, Cave, Cordero.

### Cymatoderella patagoniae

Taxon classificationAnimaliaColeopteraCleridae

(Knull, 1946)

Figs 3D , 17C–D , 19D

#### Synonyms.

*Tillus patagoniae* Knull, 1946. A new species of *Tillus* from Arizona (Coleoptera: Cleridae). Ohio Journal. Sc. 46(2): 72 1951.

#### Paratypes.

Eighteen males and 12 females examined.

#### Type locality.

Arizona, Patagonia Mountains, Santa Cruz Co. Type depository: Field Museum of Natural History (FMNH).

#### Distribution.

USA: Arizona; Mexico: Guerrero*, Jalisco, Michoacan*, Morelos, Sonora.

#### Differential diagnosis.

*Cymatoderella patagoniae* can be differentiated from congeners based on the color of the pygidium and the elytral vestiture. *Cymatoderella patagoniae* has the pygidium testaceous to ferrugineous (Fig. 17C–D), and the elytral vestiture is pale to whitish, fine and recumbent (Fig. 3D); on the other hand, *C. collaris* and *C. morula* have the last abdominal segment brown to almost black, and the elytral disc is clothed with pale, yellowish to testaceous, semirecumbent setae interspersed with some semierect setae.

#### Redescription.

Male. Form: Small and robust, elytra gradually expanded toward apex, abruptly narrowing behind the posterior fourth. Color: Head and scutellum ranging from uniformly testaceous, ferruginous, with different tones of dark testaceous, light brown, to completely piceous integument; mouthparts with various tones of piceous to brown tones; pronotum uniformly testaceous; legs, thorax and elytra piceous; abdominal segments 1-5 dark testaceous to ferrugineous, pygidium testaceous to ferrugineous; antennae uniformly piceous, Elytral disc devoid of any bands or fasciae (Fig. 3D).

Head: Including eyes wider than pronotum; eyes of moderate size, taller than wide, bulging laterally, finely faceted, emarginate posteriorly; antennal notch located in front of emargination; frons bi-impressed; integument shiny, finely, sparsely punctate, punctations small, shallow; conspicuously clothed with fine, whitish, semirecumbent setae interspersed with some erect, pale setae located around eyes; antennae with 11 antennomeres; antennomeres 2-4 short, robust, subequal in length; fourth antennomere about 3× the length of fifth antennomere; antennomeres 5-10 robust, somewhat serrate, subequal in length; last antennomere elongate, robust, obtusely rounded, 1.5× longer than tenth antennomere.

Thorax: Pronotum bisinuate, widest at middle; sides constricted subapically, more strongly constricted behind middle and constricted in front of middle; surface shiny, rugulose, profusely clothed with fine, short, pale, semirecumbent setae intermixed with some long, erect, fine, pale setae; moderately punctate; punctations wide and shallow; anterior transverse depression present, subbasal tumescence absent; compressed posteriorly. Prosternum conspicuously wider than long; smooth; polished; feebly carinate; devoid of punctation; glabrous. Mesoventrite rugulose, vested with fine, pale, semi-erect setae; punctations coarse and deep. Metaventrite strongly convex, surface shiny, rugulose, inconspicuously vested with fine, pale, recumbent setae; longitudinal depression and metaventral process present. Metepisternum hidden throughout its length. Scutellum elongate, clothed with pale, fine, semirecumbent setae.

Elytra: Broader than pronotum, slightly elongate, broader than long; humeri indicated, rounded; sides gradually broadening toward distal end, broadest on posterior fourth, then abruptly narrowing toward apex behind posterior fourth; disc flat above; surface shiny, smooth; elytral apices subtriangular; inconspicuously dehiscent; elytral declivity relatively gradual; surface conspicuously vested with fine, short, whitish, recumbent setae interspersed with some whitish, fine, long, erect setae; surface punctate, punctations arranged in regular striae; sculpturing consists of moderately coarse, shallow punctations arranged in regular striae that gradually reduce in size and depth toward elytral apex and completely disappear on posterior sixth; interstices at elytral base about 2.5× the width of punctation; interstices shiny, rugulose.

Legs: Femora shiny, rugulose, feebly swollen, clothed with some whitish, fine, semirecumbent and semi-erect setae; tibiae longitudinally rugulose, vestiture similar to but more abundant than femora.

Abdomen: Six visible ventrites. Ventrites 1-4 shiny, smooth, polished, convex, subquadrate, slightly punctate, clothed with fine, long, yellowish pale, recumbent setae; not compressed laterally; posterior margins truncate. Fifth visible ventrite subtriangular; shiny; smooth; polished; surface convex; vested with fine, long, pale, recumbent setae; lateral margins strongly oblique, arcuate; posterior margin broadly, shallowly emarginate. Sixth visible ventrite small, moderately to strongly rugulose; surface flat, finely punctate; clothed with short, pale, fine, semi-erect setae intermingled with some long, pale, erect setae; conspicuously broader than long; lateral margins strongly oblique, arcuate; posterior margin broadly, somewhat deeply emarginate; posterolateral angles broadly rounded (Fig. 17D). Fifth tergite subquadrate, convex; glabrous; punctate; posterior margin truncate. Sixth tergite subquadrate; wider than long; convex; clothed with fine, pale, recumbent setae; surface finely punctate; lateral margins oblique, posterior margin truncate to semicircularly emarginate, posterolateral angles rounded; some long, erect, dark, stout setae located along the posterolateral margins (Fig. 17C). Sixth tergite extending beyond apical margin of sixth visible ventrite, fully covering sixth ventrite in dorsal view.

Aedeagus: Phallobasic apodeme present; phallus with copulatory piece tapered at apex; phallic plate unarmed, devoid of denticles; intraspicular plate present, elongate; phallobasic apodeme short, expanded distally; phallobase subparallel; parameres free; tegmen incomplete, partially covering phallus; parameres pointed distally; endophallic struts long, slightly longer the length of tegmen; endophallic struts in horizontal position in relation to tegmen when in horizontal view; endophallic struts robust distally (Fig. 19D).

Sexual dimorphism: Females have the sixth visible abdominal segment broadly rounded posteriorly, rather than broadly, shallowly emarginate, as observed in males.

#### Material examined.

PARATYPES: 18 males, 12 females: Patagonia Mountains, AZ, VII-2-3, D. J. and J. N. Knull.

#### Additional material examined.

USA: 1 male, 1 female: Peloncillos Mountains, AZ, 33 mi E of Douglas, VII-17-1973, S. McCleve. MEXICO: 2 males, 3 female: Sonora, Mexico, Highways 15, 12 mi N of Hermosillo, 14-VIII-1965, G. H. Nelson, on *Olneya tesota* Gray; 1 male: Jalisco, Mexico, 22 km SW Llano Grande, 270 m, VI-28-1995, R. L. Westcott; 1 specimen: Michoacán, 10.6 mi S Uruapan, August 8, 1978, Plitt and Schaffner; 1 specimen: Guerrero, 10-12 km E Xochipala, 795–885 m, N17.48 W98.24-25, June 30, 1992, C. L. Bellamy.

#### Remarks.

Rifkind (1993a) examined material identified by him as *C. patagoniae* that was collected in the western portion of the Mexican state of Jalisco. We compared one of those specimens with the extensive type series Knull (1946) designated as *C. patagoniae* and this specimen matches Klug’s original description. Rifkind (*pers. comm.)* also mentioned that material of *C. patagoniae* has been collected from the Mexican states of Guerrero and Michoacan. Finally, Toledo et al. (2014) reported the existence of this species in the state of Morelos. Consequently, the geographic range of this species is extended to central Mexico.

### Lecontella

Taxon classificationAnimaliaColeopteraCleridae

Wolcott & Chapin, 1918

#### Type species.

Cymatodera (Lecontella) cancellata LeConte, 1854, original designation.

#### Distribution.

Shown in Fig. 21I.

#### Differential diagnosis.

*Lecontella* resembles various members of *Cymatodera*, but it can be differentiated from species of this genus if the elytral punctations are coarse, deep, and extending to the elytral declivity (Figs 3E–F, 4A, 7G, 12 C, E–F), and the antennae are moderately serrate with antennomeres 2-10 feebly to conspicuously compacted (Figs 9F, 10A–B). Species of *Cymatodera*, on the other hand, have the elytral disc moderately punctate, the striae almost never reach the elytral apex (Figs 4F, 5E, 13C–D), the antennal shape ranges from filiform to variously serrate, and antennomeres 2-10 are not compacted (Figs 10G–H).

#### Redescription.

Size: 8–28 mm. Color: Body uniformly fuscous to testaceous except abdomen, slightly lighter than rest of the body, integument can range from brown-testaceous to almost ferrugineous in some individuals. Elytral disc with fasciae or maculae absent. Body: Winged species, body elongate, somewhat robust.

Head: Including eye slightly wider than pronotum; integument coarsely punctate, punctations vary from narrow to wide; eyes large, coarsely faceted, feebly emarginate anteriorly conspicuously bulging laterally; antennae moderately to strongly serrate, consisting of 11 antennomeres; antennomeres 2-10 variously compacted (Figs 9F, 10A–B); frons bi-impressed or not; terminal labial palpi securiform; terminal maxillary palpi cylindrical, compressed laterally.

Thorax: Pronotum deeply punctate, punctation may range from narrow to conspicuously wide (Fig. 12E–F); pronotum widest at middle, sides more constricted behind middle. Mesepisternum fully covered by elytron in lateral view (Fig. 12C). Prosternum rugose to smooth; slightly punctate, punctation coarse. Mesoventrite wider than long; smooth; feebly punctate, punctations coarse. Metaventrite wider than long; surface conspicuously punctate; punctation moderately wide. Metaventral process compressed anteriorly. Metepisternum hidden by elytra throughout its length in lateral view.

Elytra: Elongate, subparallel, slightly broader on posterior third; surface coarsely punctate (Fig. 7G), punctations arranged in regular striae, punctations extending to apex; scutellum ovoid, compressed; vested; epipleural fold complete, narrowing toward apex.

Legs: Femora moderately to coarsely rugose; rather swollen. Tibiae slender rugulose to rugose; pulvillar formula 4-4-4; two tarsal denticles, tarsal denticles trigonal in shape; feebly to strongly vested.

Abdomen: Six visible ventrites. Ventrites 1-4 not impressed laterally; pygidium of males feebly differentiated form that of females.

Aedeagus: Sclerotized; length of aedeagus shorter than the length of abdomen; tegmen triangular; phallic plate devoid of denticles; phallobasic apodeme short, as long as or longer than phallus; endophallic struts enlarged, swollen distally.

#### Remarks.

Wolcott and Chapin (1918) established the genus *Lecontella*, designating Lecontella (Cymatodera) cancellata (LeConte) as the type species; subsequently, *L. cancellata* was synonymized by Ekis (1975) with *L. brunnea* (Spinola). The genus is currently composed of two species: *Lecontеlla brunnea* (Spinola), a species originally described as Cymatodera longicornis var. brunnea by Melsheimer (1846), later on transferred to *Lecontella* by Wolcott and Chapin (1918) and the current type species for the genus, and *L. gnara* Wolcott, 1927. Based on an extensive examination of material identified as *Cymatodera striatopunctata* Chevrolat, a third species is designated to *Lecontella* in this revision. This change is based on the close similarities on elytral punctations (Fig. 4A), antennae (Fig. 10B), and aedeagus (Fig. 20A) of *C. cancellata* with *L. brunnea* (Figs 3E, 9F, 19E) and *L. gnara* (Figs 3F, 10A, 19F).

Mawdsley (2002) has indicated that the larvae of *L. brunnea* can be parasites in nests of solitary bees and wasps. Additionally, immature stages of *L. brunnea* were observed preying on larvae of wood-boring species of the Cerambycidae and Buprestidae families. Adults of *Lecontella* species are commonly attracted to lights.

#### Key to species of *Lecontella* Wolcott and Chapin

**Table:** 

1	Punctations on pronotal disc coarse, deep and wide (Fig. 12F)	***Lecontella gnara***
–	Punctations on pronotal disc small, fine and shallow (Fig. 12E)	**2**
2(1)	Male antennomeres 2-10 moderately compacted and robust, last antennomere of males cylindrical, elongate, flattened apically, at least 4-5× the length of tenth antennomere (Fig. 9F); antennae as long as combined length of head and pronotum; length of last antennomere of female 2-3× shorter than the length of last antennomere of male	***Lecontella brunnea***
–	Male antennomeres 2-10 conspicuously compacted and robust, last antennomere of males cylindrical, robust, moderately elongate, not flattened apically, at least 3-4× the length of tenth antennomere (Fig. 10B); antennae shorter than combined length of head and pronotum; the length of last antennomere of female 2× shorter than the length of last antennomere of male	***Lecontella striatopunctata***

### Lecontella brunnea

Taxon classificationAnimaliaColeopteraCleridae

(Spinola, 1844)

Figs 3E , 9F , 12E , 19E

#### Synonyms.

Cymatodera longicornis var. brunnea (Melsheimer, 1846) nec Spinola 1844, Proc. Acad. Philad., II-12, 1844-45 (1846) p. 306 (*Cymatodera*), synonymized by LeConte (1854), and transferred to *Lecontella* by Wolcott and Dybas (1943); *Cymatodera cancellata* LeConte, 1854, Proc. Acad. Philad. VII, 1854, p. 81 (*Cymatodera*), synonymized by Wolcott (1921).

#### Type material not examined.

#### Type locality.

Brownsville, Texas. Type depository: Italy, Torino, Museo Regionale di Scienze Naturali (SCUT).

#### Distribution.

USA: AR, FL, GA, IA, IN, KS, ME, MI, MO, NC, NJ, OH, OK, PA, TX, VA; Mexico: Baja California, Jalisco, Michoacan, Morelos, Nayarit, Nuevo Leon, Oaxaca, Sinaloa, Sonora, Tamaulipas.

#### Differential diagnosis.

*Lecontella brunnea* is most similar to *L. gnara*. The two species are partially sympatric but can be differentiated based on the structure of the pronotal punctations and the antennae. The pronotal punctations on *L. brunnea* are conspicuously numerous and small (Fig. 12E), while in *L. gnara* these punctations are scarce, coarse, deep and broad (Fig. 12F). In addition, antennomeres 3-5 of *L. brunnea* are somewhat slender, moderately compacted and serrate, serration increasing toward the distal end (Fig. 9F); on the other hand, specimens of *L. gnara* have the antennomeres 3-5 somewhat robust, compacted, and feebly serrate (Fig. 10A).

#### Redescription.

Male. Form: Medium-sized to large, slightly robust. Color: Head, pronotum, thorax, scutellum, legs and antennae light brown to almost black; elytra light brown to brunneous; mouthparts fuscous, posterior half of mandibles piceous; abdominal segments light testaceous to brown; elytral disc devoid of any bands or fasciae (Fig. 3E).

Head: Including eyes wider than pronotum; eyes large, taller than wide, bulging laterally, coarsely faceted, emarginate posteriorly; antennal notch located in front of emargination; frons bi-impressed; integument coarsely, conspicuously, deeply punctate; clothed with fine, whitish, semirecumbent setae interspersed with some erect, pale setae; antennomeres robust; antennae consisting of 11 antennomeres; antennomeres 2-10 about same length, gradually increasing in width toward distal end; last antennomere of males sexually dimorphic, conspicuously elongate, robust, parallel, cylindrical, posterior portion rounded 4-6× longer than length of tenth antennomere.

Thorax: Pronotum bisinuate, widest at middle, moderately short in length; sides constricted subapically, tapered, widest in the middle; surface rugulose, conspicuously punctate, punctations small and deep (Fig. 12E), clothed with fine, short, pale, recumbent setae intermixed with some long, erect, fine, pale setae, long setae more abundant on lateral area of pronotum; anterior transverse depression present, subbasal tumescence absent; posterior margin of pronotum compressed. Prosternum conspicuously wider than long; smooth; polished; very feebly punctate to absent; surface vested to glabrous. Mesoventrite with surface rugulose, vested with fine, pale, semi-erect setae, coarsely, conspicuously punctate, punctations wide and deep. Metaventrite surface rugulose, convex; numerously, coarsely punctate; vested with fine, pale, recumbent setae; longitudinal depression and metaventral process present. Scutellum elongate, clothed with pale, fine, semirecumbent setae, compressed medially.

Elytra: Broader than pronotum; elongate; humeri indicated, rounded; sides inconspicuously broadening toward distal end, broadest on posterior third, then abruptly narrowing toward apex at posterior fourth; disc flat above; surface rugose to rugulose; elytral apices subtriangular; inconspicuously dehiscent; elytral declivity moderately steep; surface conspicuously vested with fine, short, whitish, recumbent setae sporadically interspersed with some whitish, fine, long, erect setae; conspicuously, coarsely punctate, punctations arranged in regular striae; sculpturing consisting of coarse, deep, wide punctations arranged in regular striae of the same size through the length of the striae, punctation reaching the elytral apex; interstices at elytral base about 0.5× the width of punctation; interstices rugulose. Epipleural fold gradually narrowing toward apex, last sixth moderately crenulate.

Legs: Femora rugose; feebly swollen; clothed with some whitish, fine, semirecumbent and semi-erect setae; integument conspicuously punctate, punctations small and shallow. Tibiae rugulose; punctate; punctations shallow and small; vestiture consisting of stiff recumbent and semirecumbent setae.

Abdomen: Six visible ventrites. Ventrites 1-4 shiny, smooth, polished, convex, subquadrate, punctate, clothed with fine, long, yellowish pale, recumbent setae; not compressed laterally; posterior margins truncate. Posterior margin of first visible ventrite conspicuously elevated with a transverse carina originating next to posterolateral angles producing a broad, deep, arcuate emargination. Fifth visible ventrite subtriangular; shiny; surface rugulose, convex, punctate, punctations shallow and moderately small; vested with fine, short pale, recumbent setae; lateral margins strongly oblique, arcuate; posterior margin broadly, shallowly emarginate. Sixth visible ventrite small, subtriangular in shape; feebly to strongly rugulose; surface convex, finely punctate; clothed with short, pale, fine, semierect setae; broader than long; lateral margins strongly oblique, arcuate; posterior margin short, broadly, deeply, V-shaped emarginate. Fifth tergite subtriangular, surface convex, shiny, conspicuously rugulose; punctate; posterior margin truncate. Sixth tergite subtriangular; rugose; longer than wide; surface convex; clothed with fine, pale, recumbent setae; surface finely punctate; lateral margins strongly oblique, posterior margin small, nearly acuminate, inconspicuously truncate. Sixth tergite slightly extending beyond apical margin of sixth visible ventrite, fully covering sixth ventrite in dorsal view.

Aedeagus: Phallobasic apodeme present; phallus with copulatory piece conspicuously swollen at apex; phallic plate unarmed, devoid of denticles; intraspicular plate present, elongate; phallobasic apodeme long, feebly expanded distally; phallobase subparallel; parameres free; tegmen complete, fully covering phallus; parameres pointed distally; endophallic struts long, slender distally (Fig. 19E).

Sexual dimorphism: Females of *L. brunnea* can be differentiated from males based on differences on the last abdominal segment. In females the eleventh antennomere is broadly rounded, while in males this antennomere is somewhat triangular in shape, with the lateral margins moderately to strongly oblique, and the posterior margin short and V-shaped emarginate. In addition, females have the eleventh antennomere short, slightly robust, rounded, and longer than the tenth antennomere, while males have the last antennomere cylindrical, not compressed medially and conspicuously longer than tenth antennomere.

#### Material examined.

USA: 12 males, 16 females: San Bernardino Co., CA, Joshua Tree Nat. Park, 12-V to 25-VIII-2010, Black light, E. Sadler; 2 females: New Braunfels, TX, VI7, H. Mittendorf; 1 male: Bethel, TX, [no collecting date]; 1 female: S. Pines, NC, VI-29-1919, A. H. Manee; 1 female: Harrisburg, PA, VII-3-1936, J. N. Knull; 2 females: Rockville, PA, V-25-1919, J. L. Knull; 1 female: Starr Co., TX, V-25-1951, D. J. and J. N. Knull; 1 female: Mont Alto, Pennsylvania, 1-VII-1931, A. Champlain; 1 female: Uvalde Co., TX VIII-4-1934, D. J. and J. N. Knull; 2 males: Brownsville, TX, V-11-1934, J. N. Knull; 1 female: Archbold Biol. Sta., Highlands Co. FL, IX-11-1958, S. W. Frost; 1 female: Yuma, AZ, [no collecting date], W. Lipe; 1 female: Benton Co., AR, VII-5-1942, M. W. Sanderson; 1 male, 2 females: Hidalgo Co., TX, VI-8-1958, D. J. and J. N. Knull; 1 female: Alpine, TX, VIII-6 (no collecting date), D. Larsen; 1 female: El Paso, TX, VI-8-1914; F. Larsen; 1 female: Texas [no data available]; 1 male, 1 female: Fedor, TX, [no collecting data], J. D. Sherman; 1 female: TX, 1918, C. V. Riley; 1 female: TX, [no collecting date], G. Wells; 2 males: TX, 1939 [no collecting date], New Hampshire, J. D. Sherman; 1 male: Lee Co., TX, [no collection date]; 1 female: Cameron Co., TX, Sabal Palm Groove Audubon Sanctuary, VI-2-6-1986, R. M. Brattain; 1 female: Warren Co., IN, VI-1970 [no collector data]; 1 male: Brooks Co., TX, 12 m, W of Rachal, V-8-1986, N. M. Downie; 1 male: Comal Co., TX, Bulverde, mv + bl, VI-1-1998, R. Turnbow; 2 females: Kinney Co., TX, 7 mi NE Brackettville, mv + bl, VI-23-2000, R. Turnbow; 1 female: Hidalgo Co., TX, Santa Ana Ref., VI-10-1975, J. E. Wappes; 1 female: Cameron Co., TX, Near Brownsville, VI-28-1989; R. L. Heitman; 1 male, 2 females: Jackson Co., MO, Raytown, VII-4-1978, G. H. Nelson; 1 female: Jackson Co., MO, Blue Springs, at UV light, G. H. Nelson; 1 male: Starr Co., TX, Falcon Heights, at lite, VI-12-1975, H. Turnbow; 1 female: Grantsburg, IN, at black light, VII-4-1964, D. Eckert; 2 males: Monroe Co., FL, Flamingo, Everglades National Park, VII-8-1977 [no collector data]; 1 female: Aransas Co., TX, Goose Island State Park, North of Aransas Pass, VI-14-1969, R. L. Heitzman; 1 female: Highlands Co., FL, Arch Biol. Sta., IX-15-1978, L. L. Lampert, Jr; 1 male: Cameron Co., TX, Brownsville, V-22-1967, W. H. Tyson: 1 female: Columbia, MO, VI-27-1966, S. Poe; 2 males: Monroe Co., FL, Big Pine Key, 19-VI-1975, J. B. Heppner; 1 female: Lancaster Co., NE, VII-19-1984, L K. Rieske; 1 male: Comanche Co., OK, Fort Sill, West Range Near Strip 15, VII-19-2006, B. Kondratieff and W. Cranshaw; 1 male, 2 females: Comanche Co., OK, Fort Sill, Quanah Range, near twin gates, VIII-2-2006, B. Kondratieff, M. Camper and J. Owens; 1 male, 2 females: Comanche Co., OK, Fort Sill, Quanah Range, Quanah Cr., IX-16-2006; 1 male: Comanche Co., OK, Fort Sill, East Range, Nat. Res. Building, VIII-2-2006, B. Kondratieff, M. Camper and J. Owens; 1 male, 8 females: Starr Co., TX, Round Mountain, [no collecting date], Riley; 1 female: Gonzalez Co., TX, Seguin, VII-16-18-1984, K. W. Vick, bl trap; 1 female: TX, Hidalgo Co., Bentsen-Rio Grande State park, VII-1-1961, R. H. Arnett Jr and E. Van Tassell; 1 female: Pima Co., AZ, Sonoran desert Mus., IX-2-1975, R. Turnbow; 2 males: Jeff Davis Co., TX, Davies Mt., St. Park, VII-18-21-1973, F. T. Hovore; 1 female: Johnson Co., TX, Cleburne, St. Park, 7-VII-1971, G. H. Nelson and Family; 2 females: Bexar Co., TX, Leon Valley, IV-4-1971, G. H. Nelson, on *Prosopis chilensis*; 2 males, 2 females: FL, Myakka River State Park, VII-25-2000, funnel trap; Ford Co., TX, Highway 6 at Wichita River, VIII-3-6-1996, S. P. Holmes; 1 male, 1 female: Comal Co., TX, Bulverde, VI-21-26-1993, J. E. Wappes; 1 male: Hidalgo Co., TX, Anzulduas Park, Hg light, V-27-1986, Morris and Morris; 2 females: Hidalgo Co., TX, Bentsen- Rio Grande State Park, VIII-10-1996, D. J. Heffern; MEXICO: 1 female: Apatzingan [Michoacan], Mexico, VIII-5-1942, H. Hoogstraag; 2 males, 1 female: Jalisco, Mexico, 14 km N Guadalajara, Ruta 54, Posada San Isidro, VI-23, R. Miller and L. Stange; 1 male: Nayarit, Mexico, Rio Santiago, Las Andujas, VII-11-13-1991, E. Barrera; 2 males, 1 female: Oaxaca, Mexico, Dominguillo, 760 msnm, N 17 38 907 O 96 54 703, VIII-20-1998, S. Zaragoza.

### Lecontella gnara

Taxon classificationAnimaliaColeopteraCleridae

Wolcott, 1927

Figs 3F , 7G , 10A , 12C, F , 19F

#### Synonyms.

*Cymatodera cilindricollis* Spinola, 1844, nec. Chevrolat 1833, no. 11 (*Cymatodera*). “Mexique” Synonymized by Ekis (1975).

#### Paratypes.

Two females examined.

#### Type locality.

Sabinas Canyon, Tucson, Arizona. Type depository: Field Museum of Natural History (FMNH).

#### Distribution.

USA: AZ, CA, NM, NV, TX, UT; Mexico: Baja California, Sonora.

#### Differential diagnosis.

*Lecontella gnara* is most closely related to *L. brunnea*. Characters to distinguish these species are given in the diagnosis section of *L. brunnea*.

#### Redescription.

Male. Form: Medium-sized to large, somewhat robust. Color: Head, pronotum, thorax, scutellum, legs, antennae and elytra light testaceous to dark brown; mouthparts fuscous, last fourth of mandibles piceous to black; abdominal segments testaceous to piceous; elytral disc devoid of any bands or fasciae (Fig. 3F).

Head: Including eyes wider than pronotum; eyes large, taller than wide, bulging laterally, coarsely faceted, emarginate posteriorly; antennal notch located in front of emargination; frons bi-impressed; integument coarsely, conspicuously, deeply punctate; clothed with fine, pale, recumbent setae interspersed with some erect, pale setae; antennae with 11 antennomeres; antennomeres 2-10 moderately robust, about the same length, gradually increasing in width toward distal end; antennomeres 1-4 cylindrical; serration in antennomeres 5-10 gradually increasing toward distal end; last antennomere of males sexually dimorphic; conspicuously elongate, robust, parallel, cylindrical, 4-6× longer than length of tenth antennomere (Fig. 10A).

Thorax: Pronotum bisinuate, widest at middle, somewhat short in length; sides constricted subapically, more strongly constricted behind middle and feebly constricted in front of middle; surface conspicuously punctate; elytral disc with punctations coarse, deep interspersed with a smooth disc (Fig. 12F); clothed with fine, short, pale, recumbent setae intermingled with some long, erect, fine, pale setae, long setae more abundant on anterior and lateral area of pronotum; anterior transverse depression present; subbasal tumescence absent; posterior margin of pronotum compressed. Prosternum conspicuously wider than long; moderately to strongly punctate, punctation fine, deep; surface vested to glabrous. Mesoventrite surface rugulose, scarcely vested with fine, pale, semi-erect setae; coarsely top very coarsely punctate, punctations wide, deep. Metaventrite surface smooth to rugulose, convex; rather punctate; punctations coarse, shallow to deep; vested with fine, pale, recumbent setae; longitudinal depression and metaventral process present. Scutellum elongate, clothed with pale, fine, semirecumbent setae, compressed medially.

Elytra: Broader than pronotum; elongate; humeri indicated, rounded; sides inconspicuously broadening toward distal end, broadest on posterior third, then abruptly narrowing toward apex at posterior fourth; disc flat above; surface rugose to rugulose at interstices; elytral apices subtriangular; inconspicuously dehiscent; elytral declivity moderately steep; surface vested with fine, short, pale, recumbent setae interspersed with some pale, fine, long, erect setae; conspicuously, coarsely punctate, punctations consisting of coarse, deep, wide punctations arranged in regular striae of the same size that decrease in size at elytral declivity, punctation reach the elytral apex (Fig. 7G); interstices at elytral base as wide as the width of punctation; interstices smooth. Epipleural fold gradually narrowing toward apex, last sixth moderately crenulate.

Legs: Femora rugulose; feebly swollen posteriorly; clothed with some whitish, fine, semirecumbent and semi-erect setae; surface conspicuously punctate; punctations small and shallow. Tibiae rugulose; punctations shallow and small; vestiture consisting of short, recumbent and semirecumbent setae.

Abdomen: Six visible ventrites. First ventrite coarsely rugose to rugulose. Ventrites 2-4 rugulose; convex; subquadrate; clothed with fine, long, yellowish pale, recumbent setae; not compressed laterally; posterior margins truncate. Posterior margin of first and second visible ventrites elevated with a transverse carina originating next to posterolateral angles, producing a broad, elevated arcuate emargination. Fifth visible ventrite subtriangular; shiny; surface rugulose, convex; punctations shallow, small; vested with fine, pale, recumbent setae; lateral margins oblique, feebly arcuate; posterior margin broadly, shallowly emarginate. Sixth visible ventrite small, subtriangular in shape; rugulose to rugose; surface convex, moderately, finely punctate; clothed with short, pale, fine, recumbent setae; broader than long; lateral margins oblique, arcuate; posterior margin short, broadly, shallowly, emarginate. Fifth tergite subquadrate, surface convex, shiny, finely rugulose; punctate; posterior margin truncate. Sixth tergite subquadrate; rugulose to rugose; wider than long; surface convex; clothed with fine, pale, recumbent setae; surface punctate; lateral margins oblique; posterior margin broadly, shallowly emarginate. Sixth tergite extending beyond apical margin of sixth visible ventrite, covering sixth ventrite in dorsal view.

Aedeagus: Phallobasic apodeme present; phallus with copulatory piece swollen at apex; phallic plate unarmed; intraspicular plate present, elongate; phallobasic apodeme long, slightly expanded distally; phallobase subparallel; parameres free; tegmen complete, completely covering phallus; parameres pointed distally; endophallic struts long, slender distally (Fig. 19F).

Sexual dimorphism: Females of *L. gnara* can be differentiated from males based on the shape of the last abdominal segment. The sixth visible segment in females is broadly rounded, while males have this segment somewhat subquadrate with the posterior margin broadly, very shallowly emarginate. In addition, females of *L. gnara* have the eleventh antennomere short, somewhat robust and longer than the tenth antennomere; males, on the other side, have the last antennomere cylindrical, not compressed medially and conspicuously longer than the tenth antennomere.

#### Material examined.

PARATYPE: 1 female: Sabino Canyon, Tucson, Arizona, 15-VII-1915, L. Liebeck; PARATYPE: 1 female: Copper Basin, near Prescott, Arizona, 9-IX-1907, J. A. Krusche.

#### Additional material examined.

1 female: Arnett Creek, AZ, S. W. Superior, VIII-13-1972, L. J. Bayer; 1 male: Chiricahua Mountains, AZ, VII-20-1953, D. J. and J. N. Knull; 2 males: Chiricahua Mountains, AZ, VII-22-1957, D. J. and J. N. Knull; 1 male: Chiricahua Mountains, AZ, VII-17-1957, D. J. and J. N. Knull; 1 male, 1 female: Chiricahua Mountains, AZ, VIII-8-1952, D. J. and J. N. Knull; 1 male: Chiricahua Mountains, AZ, VIII-26, D. J. and J. N. Knull; 1 male, 3 females: Chiricahua Mountains, AZ, VII-9-1959, D. J. and J. N. Knull; 1 female: Chiricahua Mountains, AZ, VII-14-1957, D. J. and J. N. Knull; 2 males, 1 female: Chiricahua Mountains, AZ, VII-15-1961, D. J. and J. N. Knull; 3 females: Chiricahua Mountains, AZ, VII-22-1961, D. J. and J. N. Knull; 1 male, 3 females: Chiricahua Mountains, AZ, VIII-28-1962, D. J. and J. N. Knull; 2 males, 1 female: Tucson, AZ, VII-29, J. N. Knull; 2 males: Tucson, AZ, VIII-10, J. N. Knull; 2 females: Chiricahua Mountains, AZ, VII-11, D. J. and J. N. Knull; 1 female: Chiricahua Mountains, AZ, VIII-12-1952, D. J. and J. N. Knull; 2 male, 3 females: Tucson, AZ, VIII-27, D. J. and J. N. Knull; 2 females: Chiricahua Mountains, AZ, VII-27-1953, D. J. and J. N. Knull; 1 male: Chisos Mountains, TX, Oak Spring, VIII-15-1962, C. A. Triplehorn; 2 females: Chiricahua Mountains, AZ, VIII-2-1952, D. J. and J. N. Knull; 1 female: Chiricahua Mountains, AZ, VIII-28, D. J. and J. N. Knull; 1 female: Tucson, AZ, VIII-13; J. N. Knull; 1 female: Tucson, AZ, VIII-27, D. J. and J. N. Knull; 1 female: Tucson, AZ, VII-11, J. N. Knull; 2 males: Tucson, AZ, VII-14, J. N. Knull; 2 males: Chiricahua Mountains, AZ, VIII-21-1962, D. J. and J. N. Knull; 1 male: Tumacacori Mountains, AZ, VIII-2-1962; D. J. and J. N. Knull; 2 females: Sabino Canyon, AZ, VIII-15-1945, Tucker; 1 female: Tucson, AZ, VIII-6, J. N. Knull; 1 male: Wickenburg, AZ, VII-25, D. J. and J. N. Knull; 1 male: Tucson, AZ, VIII-6-1913, J. N. Knull; 1 female: Tucson, AZ, 26-VII-1920, J. N. Knull; 2 females: Huachuca Mountains, AZ, 25-VII, J. N. Knull; 3 female: Chiricahua Mountains, AZ, IX-4-1962, D. J. and J. N. Knull; 1 male, 2 females: Chiricahua Mountains, AZ, VII-30-1959, D. J. and J. N. Knull; 2 females: Chiricahua Mountains, AZ, VIII-7-1959, D. J. and J. N. Knull; 1 male, 1 female: Chiricahua Mountains, AZ, VII-10-1961, D. J. and J. N. Knull; 4 males, 2 females: Chiricahua Mountains, AZ, VIII-10-1961, D. J. and J. N. Knull; 1 male: Chiricahua Mountains, AZ, VII-18-1961, D. J. and J. N. Knull; 1 female: Chiricahua Mountains, AZ, IX-11-1962, D. J. and J. N. Knull; 2 females: Sabino Canyon, AZ, VIII-7-1962, D. J. and J. N. Knull; 1 male, 2 females: Chiricahua Mountains, AZ, VIII-14-1962, D. J. and J. N. Knull; 1 female: Tucson, AZ, VII-1929, J. N. Knull, 1 female: Tucson, AZ, VIII-1910, J. N. Knull; 1 female: Tucson, AZ, VIII-1927, J. N. Knull; 1 male: Pecos, TX, 15-VIII-1962, N. M. Downie; 1 female: Globe, AZ, 1-VII-1933, F. H. Parker; 2 males: Santa Cruz Co., AZ, Yanks Spring, 4 m SE Ruby, Pajarito Mountains, 4,000 ft, IX-5-1950, T. Cohn, P. Boone and M. Cazier; 1 male: Pima Co., AZ, El Mirador Ranch, 4 mi NW Sasabe, Baboquivari Mts., 3,900 ft, IX-3-1950, T. Cohn, P. Boone and M. Cazier; 2 females: Pima Co., AZ, Sabino Canyon, Santa Catalina Mts., 5,000 ft, VIII-6-1948, G. E. Ball; 1 male: Pima Co., AZ, 15 mi E Tucson, 2600 ft, VIII-18-1950, T. Cohn, P. Boone and M. Cazier; 1 male, 1 female: Pima Co., AZ, Madrona Canyon, Ranger Station, Rincon Mts, VIII-24-1952, 3,300 ft, G. M. Bradt; 3 males: Pima Co., AZ, Continental, VII-29-1948, G. E. Ball; 2 females: Patagonia, AZ, VII-6-1936, M. Cazier; 2 males: Hidalgo Co., NM, 18 mi N Rodeo, VII-7-1956; 1 male: Pima Co., AZ, Tucson, VII-1953, G. M. Bradt; 1 female: Patagonia, AZ, VII-6-1936, M. Cazier; 1 female: Tucson, AZ, VIII-26-1949, 2,700, G. M. Bradt; 1 female: Globe, AZ, VIII-28-1952, F. H. Parker; 1 female: Huachuca Mountains, AZ, 5,400, on palm, Chass; 1 male: Gila Bend, AZ, [no collecting date and collector data]; 2 females: Baboquivari Mountains, AZ, VII-23-1949; F. H. Parker; 1 male, 1 female: Coyote Mountains, AZ, VII-4-VIII-1916, 31°59'N 111°29'W, 3,500, [no collector data]; 1 female: Patagonia mountains, AZ, VIII-20-1949, F. H. Parker; 1 female: Huachuca Mountains, AZ, VIII-15-1949, F. H. Parker; 1 female: Pima Co., AZ, Lowell Ranger Station, VI-20-VII-1916; 1 female: Globe, AZ, VIII-26-1935, C. Parker; 1 female: Santa Cruz Co., AZ, Pajarito Mts., Pena Blanca, Canyon, VII-27-1978, 1191 m, at light, S. McCleve; 1 male, 1 female : Cochise Co., AZ, Texas Canyon, 5,300’, black lite, VIII-12-1974, S. McCleve; 1 female: Cochise Co., AZ, San Bernardino Ranch, VIII-14-1976, S. McCleve; 2 female: Cochise Co., AZ, Leslie Canyon, VIII-17-1978, S. McCleve; 1 male: Cochise Co., AZ, Texas Canyon, 5300’, black lite, VIII-12-1974; 1 female: Graham Co., AZ, Aravaipa Canyon, Turkey Creek, VIII-11-1998, MV and blacklight, F. W. Skillman Jr; 1 male, 1 female: Tortilla Mountains, AZ, 12 mi N of Tucson, Pima Co., VII-16-1966; 1 female: Pima Co., AZ, Collsal Cave Park, VIII-25-1970, K. Stephan; 1 female: Cochise Co., AZ, South Western Res. Sta., VII-6-1980, UV light, L. L. Lampert Jr; 2 males: Cochise Co., AZ, Cottonwood Canyon, mercury vapor + black light, VII-16-2000, R. Turnbow; 1 male, 1 female: La Paz Co., AZ, 12-VI-1996, Cibola NWR, D. Anderson. MEXICO: 1 male: Sonora, Mexico, Tastiota, VII-18-1952, C. and P. Vaurie; 1 male: Durango, Mexico, Rodeo, San Juan del Rio, 4,700 ft, VII-29-1947; 2 females: Sonora, Mexico, Obregon, VII-29-1952, C. P. Vaurie; 1 male, 1 female: Sonora, Mexico, Minas Nuevas, 7-VIII-1952, C. and P. Vaurie.

### Lecontella striatopunctata

Taxon classificationAnimaliaColeopteraCleridae

(Chevrolat, 1876) comb. n.

Figs 4A , 10B

#### Synonyms.

*Cymatodera striatopunctata* Chevrolat, 1876, Mémoire sur la famille des Clérites. Buquet, Paris, 51 p.

#### Type material not examined.

#### Type locality.

“Mexique”. Type depository: Muséum National d’Histoire Naturelle, Paris (MNHN).

#### Distribution.

Mexico: Jalisco, Morelos, Nayarit, Oaxaca; Central America: Guatemala.

#### Differential diagnosis.

*Lecontella striatopunctata* is most similar to *L. brunnea*. The two species are parapatric in distribution and can be misidentified as each other; however, males of *L. striatopunctata* have the antennomeres 2-10 conspicuously compacted and robust, and the eleventh antennomere is 3-4× the length of tenth antennomere (Fig. 10B). Males of *L. brunnea* have the antennae compacted, antennomeres 3-10 gradually increasing in width toward the distal end, and the eleventh antennomere is 4-5× the length of the tenth antennomere (Fig. 9F). Males and females of these species can also be differentiated based on the shape of the epipleural fold. *Lecontella striatopunctata* has the posterior portion of the epipleural fold smooth, while *L. brunnea* has the same portion of the epipleural fold moderately crenulate.

#### Redescription.

Male. Form: Small to large individuals, moderately robust. Color: Head, pronotum, thorax, scutellum, legs, antennae and elytra light testaceous to dark brown; mouthparts fuscous, last fourth of mandibles piceous to black; abdominal segments testaceous to piceous; elytral disc devoid of any bands or fasciae (Fig. 4A).

Head: Including eyes wider than pronotum; eyes large, taller than wide, bulging laterally, coarsely faceted, emarginate posteriorly; antennal notch located in front of emargination; frons bi-impressed; integument coarsely, conspicuously, shallowly punctate; clothed with fine, pale, recumbent setae intermixed with some erect, pale setae; antennae consisting of 11 antennomeres; second antennomere moderately robust, slightly shorter than third antennomere; antennomeres 3-10 serrate, conspicuously robust and compacted, about the same length; last antennomere of males sexually dimorphic, conspicuously elongate, somewhat robust, parallel, cylindrical, posterior portion rounded 4-5× longer than length of tenth antennomere.

Thorax: Pronotum bisinuate, widest at middle, slightly short in length; sides constricted subapically, more strongly constricted behind middle and feebly constricted in front of middle; surface conspicuously punctate, elytral disc with punctations small, shallow; clothed with fine, short, pale, recumbent setae interspersed with some long, semierect, fine, pale setae; long setae more abundant on anterior and lateral area of pronotum; anterior transverse depression present; subbasal tumescence absent; posterior margin of pronotum feebly compressed. Prosternum conspicuously wider than long; moderately to strongly punctate, punctation fine, deep; surface vested to glabrous. Mesoventrite surface smooth, vested with fine, pale, semi-erect setae; moderately to coarsely punctate, punctations wide, deep. Metaventrite surface smooth to finely rugulose, convex; numerously, rather punctate, punctations coarse, shallow; clothed with fine, pale, recumbent setae; longitudinal depression and metaventral process present. Scutellum wide, clothed with pale, fine, semirecumbent setae, compressed medially.

Elytra: Broader than pronotum; elongate; humeri indicated, rounded; sides inconspicuously broadening toward distal end, broadest on posterior 1/4, then abruptly narrowing toward apex at posterior 1/4; surface rugose to rugulose at interstices; elytral apices subtriangular; inconspicuously dehiscent; elytral declivity moderately steep; surface vested with fine, short, pale, recumbent setae and some pale, fine, long, erect setae; conspicuously, coarsely punctate, punctations arranged in regular striae; sculpturing consisting of coarse, deep, wide punctations arranged in regular striae that decrease in size in posterior fourth, punctation reaching elytral apex; interstices at elytral base 0.5× the width of punctation; interstices smooth. Epipleural fold gradually narrowing toward apex, not crenulate.

Legs: Femora rugose; slightly swollen on distal end; clothed with some pale, fine, semirecumbent setae mixed with some semi-erect setae; surface conspicuously punctate; punctations small, shallow. Tibiae rugulose, punctate; punctations shallow and small; vestiture consisting of fine, recumbent and semirecumbent setae.

Abdomen: Six visible ventrites. First ventrite rugulose. Ventrites 2-4 moderately to strongly rugulose, convex, subquadrate, punctate, vested with fine, long, pale, recumbent setae; not compressed laterally; posterior margins truncate. Posterior margin of first and second visible ventrites elevated with a transverse carina, this carina originating next to posterolateral angles producing a broad, elevated, arcuate emargination. Fifth visible ventrite subtriangular; surface rugulose, convex, moderately punctate, punctations shallow, small; vested with fine, pale, recumbent setae; lateral margins oblique, feebly arcuate; posterior margin broadly, shallowly emarginate. Sixth visible ventrite small, shape subtriangular; rugulose to rugose; surface convex; finely punctate; clothed with short, pale, fine, recumbent setae; as broad as long; lateral margins strongly oblique, arcuate; posterior margin short, somewhat acuminate, very shallowly emarginate. Fifth tergite subquadrate, surface convex, rugulose; posterior margin truncate. Sixth tergite subtriangular; finely to moderately rugulose; surface moderately convex; clothed with fine, pale, recumbent setae; lateral margins oblique; posterior margin truncate. Sixth tergite extending beyond apical margin of sixth visible ventrite, covering sixth ventrite in dorsal view.

Aedeagus: Phallobasic apodeme present; phallus with copulatory piece swollen at apex; phallic plate unarmed, devoid of denticles; intraspicular plate present, elongate; phallobasic apodeme long, expanded distally; phallobase subparallel; parameres free; tegmen complete, covering phallus; parameres pointed distally; endophallic struts long, slender distally (Fig. 20A).

Sexual dimorphism: Females of *L. striatopunctata* differ from males in the shape of the last abdominal segment. Females have the lateral and posterior margins of the sixth abdominal segment broadly rounded. Males have the lateral margins of the sixth abdominal segment subtriangular in shape, strongly oblique, and the posterior margin is short, almost acuminate and shallowly emarginate. In addition, females have the eleventh antennomere short, robust, obtusely rounded, and approximately 2× longer than tenth antennomere, while males have the same antennomere cylindrical, not compressed medially and 4-5× longer than the tenth antennomere.

#### Material examined.

1 male, 1 female: Morelos, Mexico, Tepalcingo, N El Limón, 18°32'18.3"N 98°56'01.7"W, 1272 m, Selva baja caducifolia, trampa de luz, VI-6-2008, M. De León; 1 male: Mexico, Jalisco, Estacion Biologica Chamela, VIII-1-2-1991, E. Giesbert; 1 female: Guerrero, Mexico, Highway 95, 5.6 km S Milpillas, [no collector data]; 3 females: Jalisco, Mexico, Estacion Biologica Chamela, VII-10-20-1985, E. Giesbert; 1 male, 1 female: Jalisco, Mexico, Estacion Biologica Chamela, X-1-2-1991, E. Giesbert; 1 male: El Progreso, Guatemala, Highway 17, vic. Morazán, 1700’, V-29 to VI-2-1989, E. Giesbert; 1 female: Zacapa, Guatemala, 12-14 km S San Lorenzo, 1000-2000’, VI-3-6-1989, E. Giesbert; 1 male: Jalisco, Mexico, Estacion Biologica Chamela, X-15-21-1987, E. Giesbert; 1 male, 1 female: Mex., Jalisco, Mexico, Chamela Estacion UNAM, X-1-2-1991, J. E. Wappes; 2 males: Jalisco, Mexico, Chamela vic. E. B. UNAM, VII-9-19-1993, J. E. Wappes; 3 females: Jalisco, Mexico, vic Chamela, 15-VII-1990, J. E. Wappes; 1 female: Zacapa, Guatemala, 12-14 km S San Lorenzo, 1-2000’, VI-3-6-1989; 1 female: Mexico, Jalisco, 17.6 km N Chamela, VII-16-1987, R. Turnbow, 2 males: Mexico, Jalisco, vic Estacion de Biologia Chamela UNAM, VII-9-14-1993, Black Light, Morris, Huether, Wappes.

#### Remarks.

Chevrolat (1876) described *Cymatodera striatopunctata* from material collected in Mexico (type locality not specified). This species is here transferred to *Lecontella* based on the compacted and serrate condition of the antennae (Fig. 10B), the broadly and deeply excavated elytral punctations that reach the elytral apex (Fig. 4A), and the overall similarities observed in the aedeagus of *C. striatopunctata* (Fig. 20A), *L. brunnea* (Fig. 19E) and *L. gnara* (Fig. 19F). Other characters similar in these species are the uniformly brown to dark brown integument color, the moderately to strongly punctate pronotum, and the male pygidium subtriangular in shape.

### Monophylla

Taxon classificationAnimaliaColeopteraCleridae

Spinola, 1841

#### Type species.

*Cymatodera megatoma* Spinola, 1841 (monotype), synonymized as Monophylla (Tillus) terminata (Say, 1835).

#### Synonyms.

*Macrotelus* Klug, 1842, type species: *Tillus terminatus* Say (monotypic), synonymized by LeConte (1849); *Elasmocerus* LeConte, 1849, type species: *Monophylla terminata* Say (original designation), unnecessary replacement name for *Monophylla* (Say, 1835).

#### Distribution.

Shown in Fig. 21J.

#### Differential diagnosis.

*Monophylla* is conspicuously different from other New World tillinids, and several morphological characters are unique to this genus. The most characteristic feature of the genus is the size of the last antennomere. Species in *Monophylla* have this antennomere conspicuously longer than remaining antennomeres combined (Fig. 10C–D). This character state is also observed in species of the African genus *Teloclerus* Schenkling (Cleridae: Tillinae). The enlarged and feebly emarginate eyes of *Teloclerus* (Fig. 4E) may serve to separate this genus from the New World *Monophylla*, where the eyes are of moderate size and conspicuously emarginate, almost dividing them into two portions (Fig. 12B). Additional characters that will serve to separate *Monophylla* from other New World Tillinae genera are the rectangular shape and strongly rugulose surface of the pronotum (Fig. 7C), and the exposed pygidium in dorsal view (Fig. 4C–D).

#### Redescription.

Size: 4–10 mm. Color: Body ranging from fuscous to ferrugineous; some specimens may possess one pale fascia on each elytron (Fig. 4B–D). Body: Winged species, elongate, slender, subparallel.

Head: Including eye slightly narrower than pronotum; integument numerously, coarsely punctate, punctations vary from narrow to wide and shallow to deep; eyes moderately small, finely faceted, strongly emarginate, emargination almost dividing each eye into two separate halves (Fig. 12B); inconspicuously bulging laterally; number of antennomeres variable, last antennomere as long as or conspicuously longer than the length of remaining antennomeres combined (Fig. 10C–D); frons bi-impressed; terminal labial palpi securiform; terminal maxillary palpi cylindrical, compressed laterally.

Thorax: Pronotum coarsely punctate, punctations may range from narrow to wide, depending on species; lateral margins subparallel, slightly constricted posteriorly (Fig. 7C). Prosternum enlarged, smooth to rugose, variously punctate. Mesoventrite wider than long, smooth to rugulose, somewhat punctate. Metaventrite wider than long, surface conspicuously punctate to almost smooth. Metaventral process not compressed anteriorly. Metepisterna largely exposed, the elytra do not coverer these plates.

Elytra: Elongate, subparallel, surface coarsely punctate, punctations numerous, irregularly arranged, punctations extending to apex; scutellum ovoid, not compressed, wider than long; epipleural fold complete, narrowing toward apex.

Legs: Femora feebly to coarsely rugose; swollen; tibiae slender; pulvillar formula 4-4-4; two tarsal denticles, inner tarsal denticles trigonal in shape.

Abdomen: Six visible ventrites. Ventrites 1-5 not impressed laterally; pygidium of males moderately differentiated from that of females; females with sixth ventrite broadly rounded; pygidium simple; pygidium covered by elytra in dorsal view.

#### Remarks.

*Monophylla* was described by Spinola (1841), assigning *Monophylla megatoma*, a species later synonymized as *Monophylla terminata* (Say), as the type species. Synonyms for the genus were subsequently proposed by Klug (1842) and LeConte (1849). Klug erected *Macrotelus* (1842) to designate *Tillus terminatus* Say as a different entity outside of species of *Tillus*. LeConte (1849) erected the monotypic genus *Elasmocerus* to synonymize Tillus (Macrotelus) terminatus Say. Both names were unnecessary replacement names for *Monophylla* and are now considered junior synonyms. Currently, the genus is composed of four species distributed in the United States, Mexico, Central America and Cuba. Due to a lack of material of *Monophylla cinctipennis* (Chevrolat 1874), this species is not covered in this study. The relatively short description given by Chevrolat (1874) is translated from French and presented here. Remaining species are redescribed here.

Sexual dimorphism is noticeable in all species comprising the genus. The form of the antennae, number of antennomeres, and differences in the shape of the pygidium will help to separate males from females (Figs 10C–D). Due to this dimorphism, keys for identification to *Monophylla* species are given for males and females, separately. It is advisable to determine the sex of the specimen before using the keys provided here. Sex determination can be achieved upon observation of the last antennomere. Males have the last antennomere conspicuously elongate and laterally compressed, with remaining segments remarkably reduced (Fig. 10C). Females, on the other side, have the last antennomere moderately enlarged, slightly longer than remaining antennomeres combined, and antennomeres 7-10 or 6-10 are strongly serrate (Fig. 10D). Additionally, the pygidium of males is subquadrate in shape and emarginate posteriorly (Fig. 17E–F), while females have this segment broadly rounded posteriorly (Fig. 17G–H).

#### Key to male species of *Monophylla* Spinola

**Table:** 

1	Large specimens, ~8 mm long; antennae consisting of 11 antennomeres; integument color mostly black, except the elytral suture and margins testaceous, and scutellum brown; head and pronotum coarsely, heavily punctate; restricted to Cuba	***Monophylla cinctipennis***
–	Smaller specimens, 4–8 mm; antennae consisting of 9 or 10 antennomeres; integument color not as above; head and pronotum variously punctate, species not found in Cuba	**2**
2(1)	Pronotum bicolored, outer region of pronotal disc testaceous to ferrugineous, median region of pronotal disc piceous to black (Fig. 4D); antennomeres 3-9 robust, antennomeres 6-9 serrate, gradually increasing in size toward distal end	***Monophylla terminata***
–	Pronotum not as above; antennae not as above, antennomeres 7-9 serrate or compacted	**3**
3(2)	Antennae composed of 9 antennomeres; antennomeres 3-6 reduced, conspicuously compacted and difficult to distinguish, antennomeres 7-8 serrate (Fig. 10C); pronotum uniformly brown to dark testaceous except the anterior and posterior edge with a narrow tint of testaceous to ferrugineous color (Fig. 4B)	***Monophylla californica***
–	Antennae composed of 10 antennomeres; antennomeres 3-9 compacted, moderately robust, serration on antennomeres 4-9 gradually increasing toward distal end; pronotum uniformly brown to dark testaceous except the anterior and posterior edge with a shade of testaceous to ferrugineous color (Fig. 4C)	***Monophylla pallipes***

#### Key to female species of *Monophylla* Spinola

**Table:** 

1	Antennae with 9 antennomeres; antennomeres 6-8 serrate, serration gradually increasing toward distal end; ninth antennomere slightly longer than the length of remaining antennomeres combined	***Monophylla californica***
–	Antennae with 10 antennomeres (Fig. 10D)	**2**
2(1)	Pronotum brown to almost piceous, a dark testaceous, narrow band of the anterior and posterior margins (Fig. 4C)	***Monophylla pallipes***
–	Pronotum bicolored, outer region of pronotal disc testaceous to ferrugineous, median region of pronotal disc piceous to black (Fig. 4D)	***Monophylla terminata***

### Monophylla californica

Taxon classificationAnimaliaColeopteraCleridae

(Fall, 1901)

Figs 4B , 10C , 12B , 20B

#### Synonyms.

*Elasmocerus californicus* Fall, 1901, Papers Calif. Acad. Sci. VIII, 251 (*Elasmocerus*).

#### Type material not examined.

#### Type locality.

Santa Cruz Mountains, Santa Cruz Co., CA. Type depository: Museum of Comparative Zoology, Harvard University (MCZC).

#### Distribution.

USA: AZ, CA, NV, OR, TX, UT, WY; Mexico: Baja California, Baja California Sur, Sonora, Morelos(?).

#### Differential diagnosis.

*Monophylla californica* is most similar to *M. pallipes*. The two species have a broad, sympatric distribution and can be misidentified. These species, however, are separated with relative ease based on the number of antennomeres. The antennae of *M. californica* are composed of 9 antennomeres, while the antennae of *M. pallipes* have 10 antennomeres.

#### Redescription.

Male. Form: Small to moderately large, slender individuals. Color: Head, antennae, scutellum and legs testaceous to fuscus; pronotum testaceous to almost black, the anterior and posterior margins of the pronotum have a narrow to somewhat wide ferrugineous to testaceous band; thorax fuscous to almost black; elytra light testaceous to almost black, each elytron with a pale, narrow fascia on the median region of the elytral disc that initiates on the epipleural fold but does not reach the elytral suture; mouthparts fuscous, abdominal segments light testaceous to piceous (Fig. 4B).

Head: Including eyes narrower than pronotum; eyes moderately small, taller than wide, slightly bulging laterally, antennal notch located in front of emargination (Fig. 12B); integument coarsely punctate; clothed with fine, pale, semi-erect setae intermixed with erect, pale setae; antennae composed of 9 antennomeres; second antennomere robust, short, antennomeres 3-6 small, conspicuously compacted; antennomeres 7-8 short, serrate, ninth antennomere noticeably enlarged, conspicuously compressed laterally, much longer that remaining antennomeres combined; last antennomere of males sexually dimorphic (Fig. 10C).

Thorax: Pronotum subparallel, widest at middle, feebly to moderately constricted toward posterior margin; surface conspicuously punctate, rugose; elytral disc flat; clothed with fine, short, pale, recumbent setae interspersed with some long and very long, semi-erect and erect, dark setae, these setae are more abundant on lateral area of pronotum; anterior transverse depression feebly slightly impressed; subbasal tumescence absent. Prosternum as long as wide; rather punctate; punctation fine, shallow; surface vested to glabrous. Mesoventrite surface feebly to coarsely punctate, vested with fine, pale, semi-erect setae; moderately punctate, punctations wide, deep. Metepisternum visible throughout its length, not covered by elytra. Metaventrite: Surface smooth medially; moderately to strongly punctate laterally; punctation wide and shallow; clothed with fine, pale, recumbent setae; longitudinal depression present, metaventral process absent. Scutellum wide; clothed with pale, fine, semirecumbent setae; compressed medially.

Elytra: Anterior margin slightly broader than pronotum; elongate; subparallel, humeri feebly indicated, rounded; sides broadening toward distal end, widest on middle third then gradually narrowing toward apex; elytral apices subtriangular; inconspicuously dehiscent; elytral declivity gradual; surface vested with fine, short, pale and dark, semi-erect setae some pale, fine, long, semi-erect setae, the latter more abundant toward epipleural fold; conspicuously, conspicuously punctate, punctations small and shallow, punctations arranged irregularly arranged that reach the elytral apex; interstices smooth. Epipleural fold gradually narrowing toward apex.

Legs: Femora rugulose to smooth, widest on middle half, laterally compressed, clothed with some pale, fine, semirecumbent setae mixed with some semi-erect setae, surface feebly punctate, punctations small and shallow. Tibiae rugulose, slender, punctate; punctations shallow and small; vestiture consisting of fine, semi-erect setae.

Abdomen: Six visible ventrites. Ventrites 1-4 convex, smooth, shiny. First visible ventrite longer than second ventrite, integument rugulose. Ventrites 2-4 subquadrate, punctate, vested with fine, long, pale, recumbent setae and some long semi-erect setae; not compressed laterally; posterior margins truncate. Fifth visible ventrite strongly convex; subquadrate in shape; moderately punctate; punctations shallow, small; vested with fine, pale, recumbent setae; lateral margins subparallel, slightly arcuate; posterior margin broadly, shallowly emarginate. Sixth visible ventrite subtriangular; surface slightly rugulose, convex to almost flat, finely punctate; vested with moderately long and long, erect setae, vestiture more abundant on anterolateral margins; lateral margins oblique, strongly arcuate; posterior margin broadly, deeply emarginate, U-shaped emargination, posterolateral angles rounded. Fifth tergite subquadrate, surface convex; posterior margin truncate. Sixth tergite subquadrate; finely rugulose; surface convex posterior median region compressed; clothed with fine, pale, recumbent setae; lateral margins oblique; posterior margin broadly, deeply emarginate, U-shaped emargination, posterolateral angles rounded. Sixth tergite extending beyond apical margin of sixth visible ventrite, covering sixth ventrite in dorsal view.

Aedeagus: Phallobasic apodeme present; phallus with copulatory piece swollen at apex; phallic plate armed with a row of denticles; intraspicular plate present, elongate; phallobasic apodeme conspicuously short, expanded distally; phallobase trigonal; parameres free; tegmen incomplete, partially covering the phallus; parameres pointed anteriorly; endophallic struts long, truncate distally (Fig. 20B).

Sexual dimorphism: Females of *M. californica* have the ninth antennomere somewhat shorter than in males. In addition, antennomeres 8-6 are larger, subtriangular in shape, and moderately serrate in females, but conspicuously reduced and compressed in males. Finally, the last abdominal segment in females is broadly rounded, rather than emarginate posteriorly, as observed in males.

#### Material examined.

USA: 1 male, 2 females: San Diego Co., CA, 3 mi E of Jacumba, VII-14-1984, G. H. Nelson; 1 male: San Diego Co., CA, 5 mi E Jacumba, VI-29-1984, on *Acacia greggii*, G. H. Nelson; 1 male: Los Angeles Co., CA, Mount Baldy, VI-23-1973, E. Giesbert; 1 male: Imperial Co., CA, 15 mi E Calexico, VI-6-1961, [no collector data]; 1 female: Globe, AZ, IX-1-1933, F. H. Parker; 2 males, 1 female: Riverside Co., CA, VIII-4-1939, A. T. McClay; 2 males: Cochise Co., AZ, 2 mi S of Portal, VII-2-1960, M. Statham; 1 female: Warners, CA, X-12-1924, R. C. Casselberry; 3 females: Imperial Co., CA, Fish Springs, I-1-1939; A. T. McClay; 1 male, 1 female: Riverside Co., CA, 4-VII-1939, beating mesquite, A. T. McClay; 1 female: Pima Co., AZ, [no collecting date and collector]; 1 male: Los Angeles Co., CA; Westwood Hills, X-5-1939; 2 males, 1 female: Huachuca Mountains, AZ, VIII-19-1950, J. N. Knull; 2 females: Yuma, AZ, IV-1-1924, J. N. Knull; 2 males, 1 female: Cottonwood, AZ, VIII-18, J. N. Knull; 2 females: Cave Creek, AZ, VIII-20-1959, J. N. Knull; 8 males, 6 females: Oak Creek Canyon, AZ, VIII-15, D. J. and J. N. Knull; Grand Teton National Park, WY, VII-14-1939, D. J. and J. N. Knull; 2 males, 2 females: Valverde Co., TX, V-6-1941, D. J. and J. N. Knull; 1 male: Calipatria, CA, II-29-1924, J. N. Knull; 1 female: Palm Springs, CA, V-19-1941, D. J. and J. N. Knull; Mecca, CA, VI-19-1948, D. J. and J. N. Knull; 1 male, 1 female: Jacumba, CA, VI-24-1954, D. J. and J. N. Knull; 3 males, 1 female: Winterhaven, CA, VI-25-1952, D. J. and J. N. Knull; 1 male, 2 females: Tucson, AZ, D. J. and J. N. Knull; 1 male, 2 female: Palm Springs, CA, VI-30-1946, D. J. and J. N. Knull; 1 male: Santa Monica Co., CA, 1942, Rivers; 4 females: Santa Cruz Co., CA, Glenwood, VI-16-1968, Tyson; 1 male: Upper City, CA, VI-5-1932, A. T. McClay; 1 female: Imperial Co., CA, I-11-1934, D. J. and J. N. Knull; Riverside Co., CA, VIII-4-1939, A. T. McClay; 2 males: Tucson, AZ, VI-20-1935, Bryant; 1 male: Globe, AZ, VII-30-1949, F. H. Parker. MEXICO: 1 male, 1 female: Baja California [Sur], Mexico, 5 mi S La Paz; VIII-24-1976, E. Giesbert; 2 female: Baja California Mexico, V-29-1987, riparian palm oasis, G. H. Nelson; 1 male: Lower California, [Mexico], Santa Rosa, [missing collecting locality and date]; 1 male: Sonora, Mexico, Minas Nuevas, VIII-7-1952, C. P. Vaurie; 2 males, 1 female: Santa Rosa, Lower California, VII, D. J. and J. N. Knull; 3 males: Morelos, Mexico, Tlaquiltenango, Huaxtla, 18.37917°N 99.04581°W, Trampa de luz, V-23-2009, V. H. Toledo; 1 male: Morelos, Mexico, Tepalcingo, El Limón, 18°31'55.8"N, 98°56'17.2"W, trampa de luz, II-17-2007, V. H. Toledo and M. A. Corona.

#### Remarks.

We have examined a number of specimens tentatively identified as *M. californica* collected in Morelos, Mexico, which would indicate a considerable range expansion for this species. Additional samples are needed to corroborate if this species has a disjunct distributional range, or the present known distribution is the result of a lack of collecting.

### Monophylla cinctipennis

Taxon classificationAnimaliaColeopteraCleridae

(Chevrolat, 1874)

#### Synonyms.

*Macrotelus cinctipennis* Chevrolat, 1874, Rev. et Mag. Zool., p. 281.

#### Type locality.

Cuba (no locality was specified). Type depository: Instituto Cubano de Zoología, Museo D. Gundlach (acronym unknown).

#### Distribution.

Cuba.

**Description.** [The original description given by Chevrolat (1874) is given below.]Color and shape: Head black, elongate, prothorax, femora (knees fourth ahtica), elytra in the suture and on the margin yellow; densely [punctate], striped [striae] elongate; eyes and antennae black; head rounded, [frons] between the eyes convex, furrow [setae] thin; prothorax scarcely longer than broad, widening; form semicylindrical, truncate; [on] hind [area] slightly rounded, long, arching, trifossulato??; scutellum punctiform brown; elytra elongate, parallel, rounded, posteriorly, the legs black. Antennae eleven articulate [composed of 11 antennomeres], first [antennomere] elongate, striped, second [antennomere] short, third [antennomere] the length of the first, a little less than fourth [antennomere], fifth [antennomere] conical, 6-10 [antennomeres] robust, short, last [antennomere 11] long, cylindrical, spongiose??

#### Remarks.

Wolcott (1910), in his notes from Chevrolat’s description of *Macrotelus cinctipennis*, mentioned the rarity of the species; he further indicated that this species was unknown to him and he had not encountered it.

### Monophylla pallipes

Taxon classificationAnimaliaColeopteraCleridae

Schaeffer, 1908

Figs 4C , 20C

#### Type material not examined.

#### Type locality.

Brownsville, Texas. Type depository: United States National History Museum (USNM).

Holotype lost. Lectotype designated by Chapin (1949).

#### Distribution.

USA: AZ, CA, TX; Mexico: Chiapas, Jalisco, Morelos, Quintana Roo, San Luis Potosi, Sinaloa, Tamaulipas, Yucatan; Central America: Costa Rica, Guatemala, Honduras; South America: Chile (introduced).

#### Differential diagnosis.

*Monophylla pallipes* is very similar to *M. californica*. The two species are sympatric in distribution; therefore, they can be misidentified when collected simultaneously. Diagnostic characters are provided in the diagnosis of *M. californica*.

#### Redescription.

Male. Form: Small to moderately large, slender individuals. Color: Head, antennae, pronotum, scutellum and legs dark testaceous to almost piceous; the anterior and posterior margins of the pronotum have a narrow ferrugineous to testaceous band; thorax ferrugineous to almost black; elytra testaceous to piceous, each elytron may have a pale to yellowish fascia on the median region of the elytral disc that initiates on the epipleural fold and does not reach the elytral suture; mouthparts fuscous, abdominal segments light testaceous to fuscous (Fig. 4C).

Head: Including eyes narrower than pronotum; eyes small, taller than wide, feebly bulging laterally, antennal notch in front of eye emargination; integument coarsely to slightly punctate; clothed with fine, pale, semirecumbent and semi-erect setae intermixed with some scattered erect, pale setae; antennae consisting of 10 antennomeres; second antennomeres robust, rather short, antennomeres 3-4 small, conspicuously compacted; antennomeres 5-9 serrate, serration and size gradually increasing toward distal end, last antennomeres noticeably enlarged, conspicuously compressed laterally, much longer than remaining antennomeres combined (Fig. 4C); last antennomere sexually dimorphic.

Thorax: Pronotum subparallel, widest at middle, constricted toward posterior margin; pronotal surface moderately to conspicuously punctate, rugose to rugulose; pronotal disc flat; clothed with fine, short, pale and dark, semirecumbent setae interspersed with some long and very long, erect, dark setae; anterior transverse depression feebly impressed, subbasal tumescence absent. Prosternum as long as wide; strongly punctate; punctation fine, shallow; surface feebly clothed. Mesoventrite: Surface moderately to coarsely punctate; punctations wide, deep; vested with fine, pale, semi-erect setae. Metepisterna visible throughout their length, not covered by elytra. Metaventrite: Convex; integument punctate laterally; punctation wide and shallow; moderately clothed with fine, pale, recumbent setae; longitudinal depression present, metaventral process absent. Scutellum wide, clothed with pale, fine, recumbent setae, compressed medially.

Elytra: Anterior margin slightly broader than pronotum; elongate; subparallel, humeri inconspicuously indicated, rounded; sides gradually expanding toward distal end, widest on middle third then narrowing toward apex; elytral apices subtriangular; inconspicuously dehiscent; elytral declivity gradual; surface clothed with fine, short, pale and dark, semi-erect and erect setae; conspicuously punctate, punctations small and shallow, irregularly arranged, punctations reaching the elytral apex; interstices smooth, narrow. Epipleural fold gradually narrowing toward apex.

Legs: Femora rugulose to smooth; expanded behind middle; laterally compressed; clothed with some pale, fine, semi erect setae; surface feebly, shallowly punctate. Tibiae rugulose, slender, puncticulate; vestiture consisting of fine, pale, semi-erect setae mingled with some pale, semirecumbent setae.

Abdomen: Six visible ventrites. Ventrites 1-4 convex, smooth, shiny. First visible ventrite longer than second visible ventrite, feebly rugulose; ventrites 2-4 subquadrate; punctate; vested with fine, long, pale, recumbent setae; not compressed laterally; posterior margins truncate. Fifth visible ventrite convex; subquadrate; surface rugulose; puncticulate; vested with fine, pale, recumbent setae; lateral margins subparallel, arcuate; posterior margin broadly, shallowly emarginate. Sixth visible ventrite subquadrate; surface slightly to moderately rugulose, convex to almost flat; feebly compressed on the median-posterior region; clothed with some long, erect setae, vestiture more abundant on anterolateral margins; lateral margins oblique, slightly arcuate; posterior margin broadly, deeply emarginate, U-shaped emarginate, posterolateral angles round. Fifth tergite subquadrate, surface convex; posterior margin truncate. Sixth tergite subquadrate; finely rugulose; surface convex posterior median region compressed; clothed with long, fine, pale and dark recumbent setae; lateral margins oblique; posterior margin broadly, moderately deeply emarginate, U-shaped emargination, posterolateral angles rounded. Sixth tergite slightly extending beyond apical margin of sixth visible ventrite, covering sixth ventrite in dorsal view.

Aedeagus: Phallobasic apodeme present; phallus with copulatory piece swollen at apex; phallic plate armed with a row of denticles; intraspicular plate present, elongate; phallobasic apodeme conspicuously short, expanded distally; phallobase sinuate; parameres free; tegmen incomplete, partially covering the phallus; parameres pointed anteriorly; endophallic struts long, conspicuously robust distally (Fig. 20C).

Sexual dimorphism: Females of *M. pallipes* differ from males based on the following characters: the tenth antennomere of females is shorter than in males; antennomeres 6-9 are larger and moderately serrate in females, but conspicuously reduced and compressed in males; and females have the last abdominal segment broadly rounded to feebly truncate, while males have this segment subquadrate in shape and emarginate posteriorly.

#### Material examined.

2 males, 2 females: Hidalgo Co., TX, IV-7-1950, D. J. and J. N. Knull; 2 males, 6 females: Brownsville, TX, V-25-1934, D. J. and J. N. Knull; 2 males, 5 females: Brownsville, TX, V-14, D. J. and J. N. Knull; 1 female: Brewster Co., TX, V-26-1948; 1 female: Cameron Co., TX, VI-4-1950, D. J. and J. N. Knull; 2 females: Corpus Christy, TX, III-30-1961, D. J. and J. N. Knull; l male: Gillespie Co., TX, IV-23, D. J. and J. N. Knull; 1 male, 2 females: Hidalgo Co., TX, III-20-1952, D. J. and J. N. Knull; 1 male, 1 female: Hidalgo Co., TX, III-29-1963, D. J. and J. N. Knull; 1 male: Hidalgo Co., TX, III-26-1953, D. J. and J. N. Knull; 1 male, 1 female: Starr Co., TX, IV-9-1963, D. J. and J. N. Knull; 2 males: Starr Co., TX, III-20-1952, D. J. and J. N. Knull; 1 female: Hidalgo Co., TX, 7-IV-1950, D. J. and J. N. Knull; 1 male, 1 female: Hidalgo Co., TX, V-23-1953, D. J. and J. N. Knull; 2 males: Jackson Co., TX, V-22, D. J. and J. N. Knull; 2 males, 1 female: Brownsville, TX, V-19, D. J. and J. N. Knull; 3 males, 2 females: Uvalde Co., TX, VI-13-1949, J. N. Knull; 1 female: Santa Cruz Co., CA, Glenwood road, VI-16-1968, W. H. Tyson; 1 female: Gillespie Co., TX, VI-1, J. N. Knull; 1 male: Brownsville, TX, V-12, D. J. and J. N. Knull; 1 male: Brownsville, TX, V-5, D. J. and J. N. Knull; 1 female: Brownsville, TX, XI-19-1911, in pasture, Garden; 1 female: Cameron Co., TX, 2 mi E Los Indios, V-13-1978, N. M. Downie; 1 female: Cameron Co., TX, Sabal Palm Grove, IV-20-30-1986, D. H. Heffern; 2 males, 3 females: San Patricio Co., TX, Welder Wildlife Ref., VII-10-20-1981, R. H. Turnbow; 3 males: Cameron Co., TX, Sabal Palm Grove Sanctuary, III-16-1981, R. H. Turnbow; 2 males: Cameron Co., TX, Palm Groove Sanctuary, Brownsville, I-1977, F. T. Hovore; 1 female: Hidalgo Co., TX, Santa Ana National Refugee vic., Willow Lake, T. C. MacRae; 1 male: Starr Co., TX, Rio Grande City, X-10-1972, E. Giesbert; 1 female: San Patricio Co., TX, Welder Wildlife Refuge, V-10-12-1977, E. Giesbert; 1 male: TX, reared from mesquite logs, emerged X-10-1955, H. F. Howden; 1 male, 1 female: TX, Lake Corpus Christy State Park, VI-19-1971; G. H. Nelson; 1 male: Cameron Co., TX, Sabal Palm Grove Sanctuary, III-20-1982, R. Turnbow. MEXICO: 1 male: Sinaloa, Mexico, 3 km E El Marmol, VIII-8-1983, E. Giesbert; 1 male, 2 females: San Luis Potosí, Mexico, 69.5 km N Tamazunchale, VI-5-1987, R. H. Turnbow; 2 males, 3 females: Jalisco, Mexico, 1.2 km S of La Cumbre, VII-19-2011, R. H. Turnbow; 2 males, 1 female: Tamaulipas, Mexico, 1-2 mi E Nuevo Morelos, VI-2-1982, R. H. Turnbow; 1 male: Guerrero, Mexico, 7.3 km NW Ixtapa, VII-17-1985, R. Turnbow; 2 males, 2 females: Quintana Roo, Mexico, highway 186, 17 km S jct. 307, V-30-1984, R. Turnbow; 1 female: Yucatan, Mexico, 2 km E Chichen Itza, V-26-1984, R. Turnbow; 1 female: Chiapas, Mexico, 4 mi NW of Pueblo Nuevo River Bajada, VII-15-1965, G. H. Nelson. CENTRAL AMERICA: 1 female: El Paraiso, Honduras, 31.5 km W Danli, VII-20-1995, R. Turnbow.

#### Remarks.

The holotype of *M. pallipes* was lost and a lectotype was designated by Chapin (1949).

### Monophylla terminata

Taxon classificationAnimaliaColeopteraCleridae

(Say, 1835)

Figs 4D , 7C , 10D , 17E–H , 20D

#### Synonyms.

Tillus (Macrotelus) terminatus Say, 1835, original designation, Bost. Journ. Nat. Hist. Mus., p. 160. Synonymized by Wolcott (1910). *Elasmocerus terminatus* (Say, 1835) designated by LeConte 1849.

#### Type material not examined.

#### Type locality.

“near Council Bluff, on the Missouri River”. Type depository: The type material was destroyed, no lectotype has been designated.

#### Distribution.

Canada: Ontario; USA: AL, AR, AZ, DC, FL, GA, IA, IL, IN, KS, KY, LA, MD, MI, MO, MS, NC, NE, NJ, NY, OK, OH, PA, RI, SC, TX, VA, WV. Mexico: Chihuahua, Sinaloa.

#### Differential diagnosis.

*Monophylla terminata* is most similar to *M. pallipes*. The two species can be recognized based on antennal differences and integument color. In males of *M. terminata*, antennomeres 3-6 are robust and compacted, and antennomeres 7-9 are serrate (Fig. 4D). In *M. pallipes*, antennomeres 3-4 are robust and clavate, and antennomere 5-9 are serrate. In addition, males and females of *M. terminata* have the pronotum bicolored, the outer region of the pronotal disc is testaceous to ferrugineous and the median region is piceous to black (Fig. 4D). In *Monophylla pallipes*, the pronotum is predominantly dark-testaceous to piceous, with the anterior and posterior margins feebly dark-ferrugineous (Fig. 4C), this coloration may be absent in some specimens, with the pronotum completely dark-ferrugineous to almost black.

#### Redescription.

Male. Form: Small to moderately large, slender individuals. Color: Head, antennae, legs and scutellum dark testaceous to almost piceous; thorax bicolored, outer region of pronotal disc testaceous to ferrugineous, median region of pronotal disc piceous to black; prosternum testaceous to ferrugineous to almost black; meso and metathorax bicolored, testaceous to ferrugineous and piceous to black; elytra fuscous to black, the anterior half of the epipleural fold testaceous to ferrugineous, some individuals with a pale to yellowish fascia on the median region of the elytral disc that initiates on the epipleural fold and does not reach the elytral suture; abdomen light testaceous to ferrugineous; mouthparts testaceous (Fig. 4D).

Head: Including eyes narrower than pronotum; eyes medium-sized, taller than wide, feebly bulging laterally, antennal notch in front of eye emargination; integument conspicuously punctate, punctation wide and shallow; vested with fine, pale, semi-erect and erect setae; antennae composed of 10 antennomeres; second antennomere robust, short; antennomeres 3-6 small, robust, conspicuously compacted; antennomeres 7-9 compacted, serrate, serration gradually increasing toward distal end; last antennomere noticeably enlarged, conspicuously compressed laterally, much longer that remaining antennomeres combined; last antennomere sexually dimorphic (Fig. 4D).

Thorax: Pronotum subparallel, expanded at middle, then constricted before posterior margin; pronotum with surface moderately to conspicuously punctate, feebly rugulose; pronotal disc flat; clothed with fine, short, pale and dark, semi-erect setae interspersed with some long and very long, erect, dark setae; anterior transverse depression feebly impressed; subbasal tumescence absent (Fig. 7C). Prosternum rugose, as long as wide; punctation fine to moderately coarse, shallow; integument clothed with fine, long, semi-erect setae. Mesoventrite surface coarsely punctate, punctation coarse and shallow, interstices smooth, shiny; vested with fine, pale, semi-erect setae Metaventrite strongly convex; surface feebly to moderately punctate, punctation small and shallow; clothed with fine, pale, semirecumbent setae; longitudinal depression present, metaventral process absent. . Metepisterna visible throughout their length, not covered by elytra. Scutellum wide, clothed with pale, fine, recumbent setae.

Elytra: Anterior margin slightly broader than pronotum; elongate; subparallel, humeri feebly indicated, rounded; sides gradually expanding toward distal end, wider on middle third, then narrowing toward apex; elytral apices subtriangular to moderately rounded; inconspicuously dehiscent to almost confluent; elytral declivity gradual; surface clothed with fine, short, pale and dark, erect setae, intermingled with some scattered long, erect setae; conspicuously punctate, punctations wide and shallow, irregularly arranged, punctations reaching the elytral apex; interstices smooth to feebly rugulose, narrow; epipleural fold gradually narrowing toward apex.

Legs: Surface of femora smooth, shiny, expanded medially, laterally compressed; feebly clothed with some pale, fine, semi-erect setae, surface shallowly punctate. Tibiae shiny, slender, puncticulate; punctations shallow and small; vestiture consisting of fine, pale, erect and semi-erect setae.

Abdomen: Six visible ventrites. Ventrites 1-4 convex, smooth, shiny, subquadrate, slightly punctate, vested with fine, short, pale, appressed setae; segments not compressed laterally; posterior margins truncate. Fifth visible ventrite convex; subquadrate; integument smooth, shiny, punctate; punctations wide and shallow; vested with fine, pale, recumbent setae; lateral margins oblique, arcuate; posterior margin broadly, shallowly emarginate. Sixth visible ventrite small, subquadrate; conspicuously wider than long; surface slightly rugulose, almost flat; clothed with some long, erect setae; vestitures more abundant on anterolateral margins; lateral margins strongly oblique, moderately arcuate; posterior margin broadly, deeply, U-shaped emarginate; posterolateral angles rounded (Fig. 17F). Fifth tergite subquadrate; surface convex; posterior margin broadly, very feeble emarginate. Sixth tergite subquadrate; finely rugulose; surface convex; clothed with some long, fine, pale, dark recumbent setae; lateral margins moderately oblique; posterior margin broadly, deeply, U-shaped emarginate; posterolateral angles rounded (Fig. 17E). Sixth tergite slightly extending beyond apical margin of sixth visible ventrite, covering sixth ventrite in dorsal view.

Aedeagus: Phallobasic apodeme present; phallus with copulatory piece feebly swollen at apex; phallic plate devoid of denticles; intraspicular plate present, elongate; phallobasic apodeme short, expanded distally; phallobase sinuate; parameres free; tegmen incomplete, partially covering the phallus; parameres rounded anteriorly; endophallic struts short, robust, truncate distally (Fig. 20D).

Sexual dimorphism: Females of *M. terminata* show a number of differences with respect to males. The most apparent is the length of the last antennomere; specifically, this antennomere is somewhat shorter in females compared with that of males; additionally, antennomeres 6-9 are moderately large and serrate in females (Fig. 10D), but conspicuously reduced and compressed in males (Fig. 4D). Finally, females have the last abdominal segment broadly rounded, while males have this segment subquadrate in shape and emarginate posteriorly (Fig. 17G–H).

#### Material examined.

1 female: Taney Co., MO, on wood pile, V-6-1955, B. Miller; 2 females: Brewster Co., TX, Chisos Mountains Basin, Big Bend Nat. Park, VI-15-1948, M. Cazier; 1 female: Terrell Co., TX, Sanderson, VI-12-1948, M. Cazier, 1 male, 1 female: Dimmit Co. TX, Catarina, VI-10-1948, M. Cazier; 1 female: NY, F. Montgomery, VI-17-1910, F. M. Schott; 1 female: Berks Co., PA, Virginville, VI-6-1968, P. Vaurie; 2 males, 1 female: Dekalb Co., GA, reared on *Vitis* sp., 1971, J. E. W.; 1 male, 1 female: TN, Coll. Chass Palm, [no collecting date, no collector data]; 1 male: 1 female: Encinal, TX, IX-13-1955, L. Downs; 1 male: Westfield, NJ, VI-8-1956, G. R. Ferguson, 4 males, 4 females: Fort Lee, NJ, 1912, [no collector data]; 1 female: Nutley, NJ, VIII-12, E. L. Dickenson; Denville, NJ, VII-1-1924, F. M. Schott; Greenwood, New Jersey, V-1930, J. A. Grossbeck; 1 male, 3 females: TX, 4 mi E of Mission, on mesquite, IV-16-1974, G. H. Nelson; 1 female: Southern Pines, NC, V-1-1923, A. H. Manee; 1 male: NY, 7-VIII-1886, [no collector data]; 1 male: Comal Co., TX, VI-12-1910, [no collector data]; 1 female: Washington, NJ, VII-16-1958, [no collector data]; 7 males, 5 females: Hummelstown, PA, VI-20-1920, J. N. Knull; 2 males, 1 female: Harrisburg, PA, V-12-1910, J. N. Knull; 1 male, 2 females: Mont Alto, PA, 29-V-1931, J. N. Knull; 1 female: n. Cumberland, PA, [no collecting data], A. Champlain; 1 male: Columbus, OH, VIII-1-1924, J. N. Knull; 4 females: Hocking Co., OH, VI-4-16, J. N. Knull; 4 males, 2 females: Key Largo, FL, V-13, J. N. Knull; 1 female: Lake Corpus Christi, TX, III-3-1961, D. J. and J. N. Knull; 1 female: Delaware Co., OH, 4-VI, D. J. and J. N. Knull; 1 female: Starr Co., TX, IV-5-1963, D. J. and J. N. Knull; 1 male: Brooks, TX, IV-10-1950, D. J. and J. N. Knull; 1 male: Uvalde Co., TX, VIII-25-1947, D. J. and J. N. Knull; 1 female: Gillespie Co., TX, V-7-1946, D. J. and J. N. Knull; 1 female: Valley, NE, VI-30-1938, D. J. and J. N. Knull; 2 males: Lemoyne, PA, III-12-1911, D. J. and J. N. Knull; 1 male: Brownsville, TX, III-15, J. N. Knull; 2 males: New York, NY, [no collecting date]; G. Beyer; 1 female: Stillwater, OK, V-21-1995, M. Gates; 1 female: Starr Co., TX, 7 mi E El Sauz, V-8-1986, N. M. Downy; 1 male: USA, Marion Co., FL, SR40, at Lynne, IV-17-2007, F. W. Skillman Jr, beating slash; 2 females: San Antonio, TX, VII-4-1968, G. H. Nelson family; Gambier, OH, VI-19-1940, [no collector data]; 2 males, 1 female: Berrien Co., GA, 3 mi E Alapaha, IV-2-4-1973, R. Turnbow; 1 female: Starr Co., TX, Falcon Heights, abandoned park, farm road 2098, [no collecting date], T. C. MacRae; 1 female: Alachua Co., FL, Gainesville, IX-24-1990, M. C. Thomas; 2 males: Taney Co., MO, Henning Cons. Area, White River Balds National Area, T. C. [no collecting date], MacRae; 1 male: Greenbrier Co., WV, Rupert, VIII-31-1992, S. F. Hutchinson; 2 males: Lawrence, KS, summer-1952, attracted to light, S. L. Wood; 1 female: Osage Co., OK, West Bartlesville, V-5-1981, K. Burnham; 1 male: Brazos Co., TX, IV-16-1960, J. N. Knull; 3 males, 4 females: Philadelphia Co., PA, [no collecting date], H. W. Wenzel; 3 males, 2 females: Hummelstown, PA, [no collecting date], J. N. Knull; 1 male: Franklin Co., OH, Columbus, VII-14-1968, [no collector data]; 1 male, 1 female: Carlisle, PA, VI-27-1917, H. R. Kirk; 1 male, 1 female: Benchley, TX, IV-30-1941, D. J. and J. N. Knull; 1 female: Columbus, OH, VI-24-1967, C. A. Triplehorn; 1 male: Kennedy, TX, IV-1944, R. Klieforth; 2 females: Cameron Co., TX, IV-3-1964, D. J. and J. N. Knull; 1 male: Lake Corpus Christy, TX, III-24-1965; 2 females: Seneca Co., OH, VII-25-1955, H. W. Hintz; 1 male: Dubuque, IA, VI-16-1955, H. Hintz; 1 male: Essex Co., Ontario, Wheatly, VI-9-1967, K. Stephan; 1 female: Ela, NC, Swan Co., 20-VIII-1954, G. B. Merrill; 1 female: Zapata Co., TX, 4 mi N San Ygnacio, VI-13-1975, R. Turnbow; 1 male: Latimer Co., OK, V-1986, K. Stephan; 1 male: Oxford, MS, V-15-1949, H. V. Weems Jr.; 1 female: Highlands Hamm Station Park, FL, III-5-1957, H. V. Weems Jr.

### Onychotillus

Taxon classificationAnimaliaColeopteraCleridae

Chapin, 1945

#### Type species.

*Onychotillus vittatus* Chapin, original designation.

#### Distribution.

Shown in Fig. 21K.

#### Differential diagnosis.

Species of *Onychotillus* somewhat resemble small-sized members of *Cymatodera* and species of *Cymatoderella*. These genera can be differentiated without difficulty based on the number of tarsal claws. *Onychotillus* species have a single tarsal claw, while members of *Cymatodera* and *Cymatoderella* have two tarsal claws (Fig. 7B). Additionally, the distribution of *Onychotillus* does not overlap with that of *Cymatodera* and *Cymatoderella*, with the former restricted to the West Indies, while the latter two have a continental distribution.

#### Redescription.

Size: 3–12 mm. Color: Body ranging from light fuscous to ferrugineous; some individuals may possess one pale, median fascia on each elytron. Body: Winged species; short to moderately long, slightly robust, somewhat subparallel specimens (Fig. 5A–B).

Head: Including eye slightly narrower than pronotum; integument coarsely punctate; punctations vary from narrow to wide; eyes large, finely faceted, feebly emarginate posteriorly, bulging laterally; antennae with 11 antennomeres, extending beyond posterior margin of pronotum; last antennomere as long as or conspicuously longer than ninth antennomere (Fig. 10E–F); frons feebly bi-impressed; terminal labial palpi securiform; terminal maxillary palpi cylindrical, distally compressed.

Thorax: Pronotum shiny, smooth to feebly punctate; punctation may range from narrow to slightly wide; lateral margins subparallel, slightly constricted anteriorly, somewhat wider posteriorly. Prosternum: Much wider than long, smooth to rugose, variously punctate. Mesoventrite: Wider than long, smooth, feebly to moderately punctate. Metaventrite: Convex, wider than long, surface conspicuously punctate to almost smooth. Metaventral process compressed anteriorly. Metepisterna largely exposed, the elytra do not coverer these plates in lateral view.

Elytra: Short to elongate, subparallel, widest on middle half, surface moderately to coarsely punctate; punctations arranged in regular striae; punctations do not extend to apex; epipleural fold complete, narrowing toward apex.

Legs: Femora smooth to rugose; rather swollen distally; tibiae rugose to rugulose; pulvillar formula 4-4-4; one tarsal denticle, tarsal denticle subtriangular in shape.

Abdomen: Six visible ventrites. Ventrites 1-5 not impressed laterally. Sixth ventrite of males feebly differentiated from that of females; females with sixth ventrite broadly rounded, pygidium simple.

#### Remarks.

*Onychotillus* is currently composed of five species, all inhabiting the West Indies. Of the five valid species, three are thought to be restricted to Cuba (De Zayas 1988). Based on the limited descriptive work given by De Zayas (1988), where no key for identification was provided, specimens of what appears to be *Onychotillus cubana* were examined (Fig. 5A); however, none of these individuals were collected in Cuba, so these specimens are tentatively assigned to *O. cubana*, pending further material to be examined. It is important to note that De Zayas’ descriptions were based, in most cases, on a single specimen, and access to that material has been particularly difficult. Therefore, due to the lack of availability of material and the poorly detailed descriptions given by De Zayas, three *Onychotillus* species are excluded from this revision, *O. trinitatis*, *O. minuta* and *O. dimidiata*, all endemic to Cuba. On the other hand, we examined material of *Onychotillus vittatus* collected in the Dominican Republic and the Cayman Islands. These collecting localities represent new distribution records for *O. vittatus*.

#### Key to species of *Onychotillus*

**Table:** 

1	Pronotal disc piceous to almost black, with or without a metallic luster (Fig. 5B); last antennal segment about the same length as the two preceding antennomeres combined (Fig. 10F)	***Onychotillus vittatus***
–	Pronotal disc light testaceous to ferrugineous, without a metallic luster (Fig. 5A); last antennal segment about the same length as the three to four preceding antennomeres combined (Fig. 10E)	***Onychotillus cubana***

### Onychotillus cubana

Taxon classificationAnimaliaColeopteraCleridae

De Zayas, 1988

Figs 5A , 10E

#### Type material not examined.

#### Type locality.

Pico Turquino, Cuba. Type depository: unknown.

#### Distribution.

Cayman Islands*, Cuba, Dominican Republic*.

#### Differential diagnosis.

*Onychotillus cubana* can be differentiated from *O. vittatus* based on the integument color, structure of the eleventh antennomere, and body size. In *O. cubana* the pronotal integument is light testaceous to ferrugineous (Fig. 5A), the eleventh antennomere is approximately 4× the length of the tenth antennomere (Fig. 10E), and body length ranges from 3 to 5 mm. *Onychotillus vittatus*, on the other side, has the pronotal integument metallic blue to almost piceous (Fig. 5B), the eleventh antennomere is about the same length as the tenth antennomere (Fig. 10F), and body length ranges from 6 to 11 mm.

#### Redescription.

Male. Form: Rather robust, short. Color: Head, antennae, mouthparts, elytra, legs, meso and metathorax light fuscous to almost piceous; pronotum and prosternum testaceous to ferrugineous; mesoventrite and abdomen, except anterior portion of first visible ventrite, testaceous; metaventrite and anterior portion of first visible ventrite light brown (Fig. 5A).

Head: Measured across eyes wider than pronotum; surface rugose; moderately, coarsely punctate; clothed with long, recumbent setae and some semierect setae behind the eyes; frons bi-impressed; eyes large, rounded, slightly taller than wide, bulging laterally; antennae extending slightly beyond base of elytra; second antennomere short, robust; third antennomere slightly longer than third antennomere; fourth antennomere about the same length as second antennomere; antennomeres 5-10 subequal in length, each about half the length of fourth antennomere; antennomere 2–4 subcylindrical; antennomeres 5–10 feebly serrate; last antennomere cylindrical, acuminate posteriorly, approximately 3.5× the length of tenth antennomere (Fig. 10E).

Thorax: Pronotum short, as wide as long; sides constricted anteriorly and subapically; widest in front of middle; disc convex; anterior transverse depression and subbasal tumescence absent; surface clothed with short and long, semierect setae; surface rugulose to shiny, conspicuously less rugose than head; shallowly punctate. Prosternum rugulose to shiny; punctations absent to very feebly punctate. Mesoventrite convex; conspicuously, coarsely punctate; clothed with long, semirecumbent setae. Metaventrite wider than long; strongly concave; rugulose; shallowly punctate; vested with fine, pale, recumbent setae.

Legs: Femora and tibiae clothed with fine, semirecumbent setae interspersed with long, semierect setae; surface of femora rugulose to smooth. Tibiae transversally rugose; fourth tarsomeres with pulvilli not incised medially.

Elytra: Base wider than pronotum; humeri indicated; sides subparallel; widest behind middle; disc convex; surface rugose; apices subtriangular, feebly dehiscent; clothed with long and short, erect setae; sculpturing consisting of coarse punctations arranged in regular striae that gradually become smaller toward apex, striae reaching elytral apex; interstices at elytral base about 2.5× the width of punctuation.

Abdomen: Six visible ventrites. First visible ventrite medially elevated; lateral portions feeble excavated; ventrites 1–4 slightly rugose, subquadrate, shallowly punctate; vested with long, fine, pale, recumbent setae. Fifth visible ventrite subquadrate; surface convex, coarsely punctate; lateral margins subparallel; posterior margin truncate. Sixth visible ventrite subquadrate; broader than long; surface rugulose; moderately, coarsely punctate; lateral margins conspicuously oblique; posterior margin broadly rounded, producing a semicircular margin. Fifth tergite rugulose; lateral margins subparallel; posterior margin truncate, with a narrow, shallow, subtriangular emargination. Sixth tergite subtriangular; broader than long; surface rugulose; lateral margins feebly arcuate, oblique; posterior margin short, rounded; lateral and posterior angles producing a round posterolateral margin; Sixth tergite extending beyond posterior margin of sixth visible ventrite, fully covering the sixth visible ventrite in dorsal view.

Aedeagus: Not available.

Sexual dimorphism: The only female examined differs from males by having the last abdominal segment broadly rounded and inconspicuously convex to almost flat, rather than subtriangular in shape and with the surface convex, as seen in males. This female also has the eleventh antennomere approximately 0.5× shorter than the same antennomere of males.

#### Material examined.

2 males, 1 female: Cayman Islands, Brac Paradise Subdivision, 19°44.688'N, 79°44.55'W, 6-VI-2008, M. C. Thomas, R. H. Turnbow and B. K. Dozier, blacklight trap; 1 female: Cayman Islands, Major Donald Dr., 4 km E jct. Ashton Reid Dr., 22-V-2009, M. C. Thomas, R. H. Turnbow; 1 male: Dominican Republic, Independencia, Sierra de Neiva, just south of crest, 5 km SW of Angel Feliz, 1780 m, 18°41'N, 71°47'W, 13-15-X-1991, J. Rawlings, R. Davidson, C. Young and S. Thomas.

### Onychotillus vittatus

Taxon classificationAnimaliaColeopteraCleridae

Chapin, 1945

Figs 5B , 10F , 17I

#### Type material not examined.

#### Type locality.

Great Goat, Jamaica. Type depository: National Museum of Natural History (NMNH).

#### Distribution.

Dominican Republic*, Jamaica.

#### Differential diagnosis.

*Onychotillus vittatus* is most similar to *O. cubana*. Characters to distinguish these species are given in the diagnosis of *O. cubana*.

#### Description.

Male. Form: Slender, moderately small, elongate individuals. Color: Head, pronotum, antennae, mouthparts, elytra, meso and metathorax metallic blue to almost piceous; legs with femora bicolored, anterior portion light testaceous to pale yellow, posterior portion metallic blue to almost black; tibiae uniformly metallic blue to almost black; abdomen uniformly piceous to black; elytral disc without fasciae or maculae (Fig. 5B).

Head: Measured across eyes narrower than pronotum; surface rugose, somewhat punctate; punctures broad and shallow; clothed with long, recumbent setae and some semirecumbent setae; frons bi-impressed; eyes large, rounded, slightly taller than wide, bulging laterally, finely faceted. Antennae extending slightly beyond anterior margin of elytra; second antennomere short, robust; third antennomere slightly longer than second antennomere; antennomeres 4-5 each about the same length as third antennomere; sixth antennomere slightly shorter than fifth antennomere; antennomeres 6-10 subequal in length; antennomere 2–5 subcylindrical; antennomeres 6–10 feebly serrate; eleventh antennomere cylindrical, acuminate posteriorly, slightly compressed medially, approximately 2× longer than the length of tenth antennomere (Fig. 10F).

Thorax: Pronotum short, as wide as long to slightly longer than wide; sides weakly constricted anteriorly and subapically; conspicuously widest in front of middle; disc convex; anterior transverse depression and subbasal tumescence absent; surface clothed with short, recumbent setae intermixed with some long, semierect setae; integument rugulose; conspicuously punctate, punctations somewhat small and shallow. Prosternum shiny, with a longitudinal carina that divides this plate; moderately excavated laterally; feebly punctate. Mesoventrite coarsely punctate; punctations wide and deep; glabrous to slightly clothed with long, semirecumbent setae. Metaventrite conspicuously wider than long; strongly concave; surface rugose; moderately, shallowly punctate; vested with fine, pale, recumbent setae.

Legs: Femora feebly rugose, shiny; clothed with fine, pale recumbent and semirecumbent setae. Tibiae transversally rugose; more conspicuously vested than femora; fourth tarsomeres with pulvilli not incised medially.

Elytra: Anterior base wider than pronotum; humeri indicated; sides subparallel; widest at middle; disc moderately convex; surface rugulose; apices rounded, slightly dehiscent; clothed with short, semirecumbent setae intermingled with some long, erect setae; sculpturing consists of coarse punctations arranged in regular striae that gradually become smaller toward apex, striae reaching elytral apex; interstices at elytral base about 2.5× the width of punctuation.

Abdomen: Six visible ventrites. First visible ventrite feebly elevated medially; anterolateral region very feeble excavated; ventrites 1–5 moderately rugose; subquadrate; shallowly punctate; vested with long, fine, pale, recumbent setae. Fifth visible ventrite with lateral margins subparallel and posterior margin truncate. Sixth visible ventrite small, subquadrate, broader than long; surface rugose; somewhat punctate; lateral margins conspicuously oblique; posterior margin broadly rounded (Fig. 17I). Fifth tergite broadly convex, rugulose; lateral margins subparallel; posterior margin truncate. Sixth tergite subquadrate, as broader as long; surface rugulose; lateral margins slightly oblique; posterior margin triangular, acuminate distally (Fig. 17I). Sixth tergite extending beyond posterior margin of sixth visible ventrite; fully covering the sixth visible ventrite from dorsal view.

Aedeagus: Not available.

Sexual dimorphism: Females of *Onychotillus vittatus* differ from males by having the eleventh antennomere approximately 2× longer than the tenth antennomere, rather than 3-3.5× longer, as in males. In addition, females have the lateral and posterior margins of the sixth visible ventrite broadly rounded, giving the appearance of a semicircular margin, rather than subtriangular in shape and posteriorly acuminate, as seen in males (Fig. 17I).

**Figure 14.**
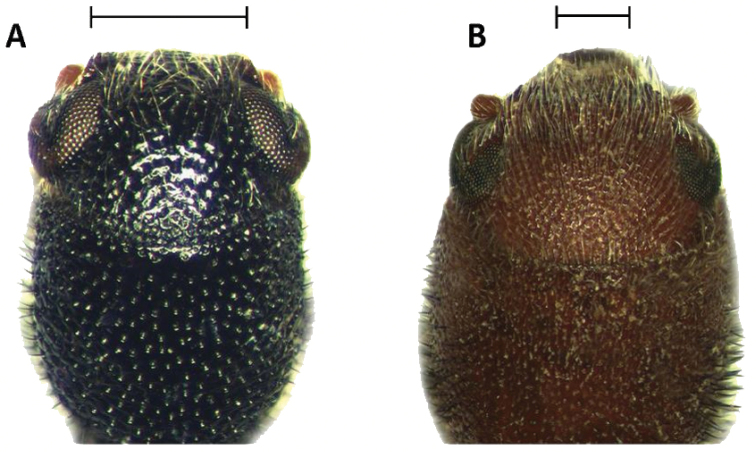
Head of: **A** *Neocallotillus elegans* **B** *Callotillus eburneocinctus*. Bars indicate width of frons.

**Figure 15.**
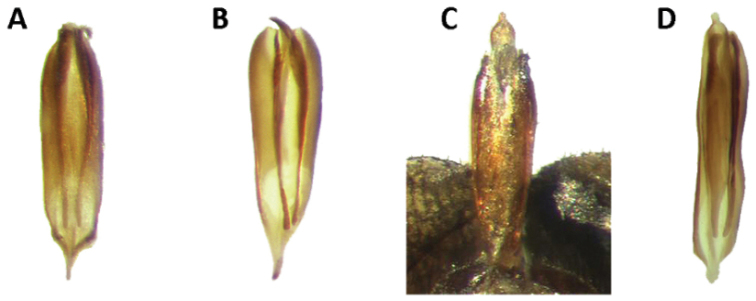
Aedeagus of: **A** *Neocallotillus elegans* (*black morph*) **B** *Neocallotillus elegans* (*bi-colored morph*) **C** *Neocallotillus intricatus* **D** *Callotillus eburneocinctus*.

**Figure 16.**
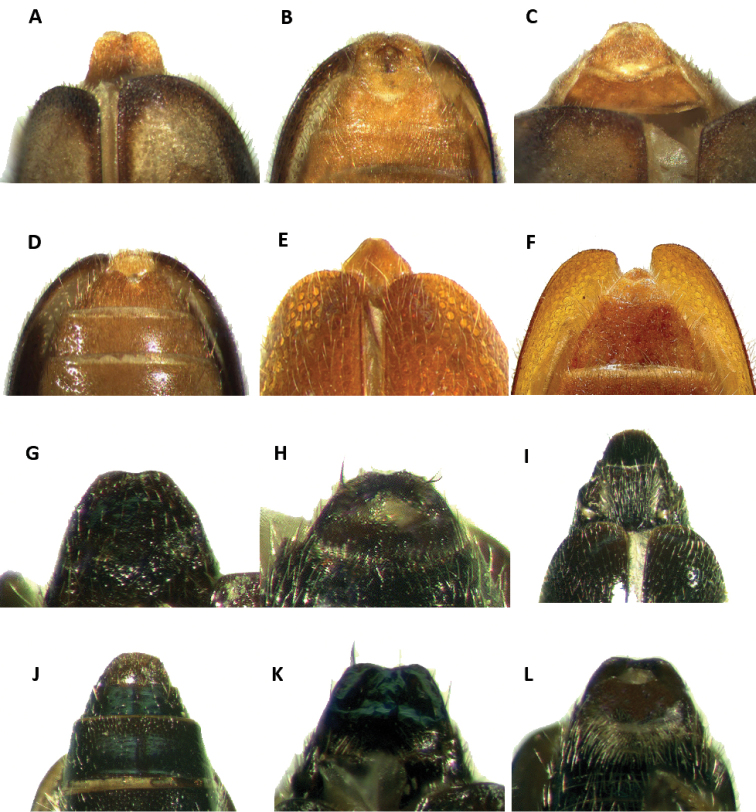
Pygidium of: **A** *Araeodontia peninsularis* dorsal (male) **B** *Araeodontia peninsularis* ventral (male) **C** *Araeodontia peninsularis* dorsal (female) **D** *Araeodontia peninsularis* ventral (female) **E** *Bogcia disjuncta* dorsal (male) **F** *Bogcia disjuncta* ventral (male) **G** *Neocallotillus elegans* (*elegans*) dorsal (male) **H** *Neocallotillus elegans* (*elegans*) ventral (male) **I** *Neocallotillus elegans* (*elegans*) dorsal (female) **J** *Neocallotillus elegans* (*elegans*) ventral (female) **K** *Neocallotillus elegans* (*vafer*) dorsal (male) **L** *Neocallotillus elegans* (*vafer*) ventral (male).

**Figure 17.**
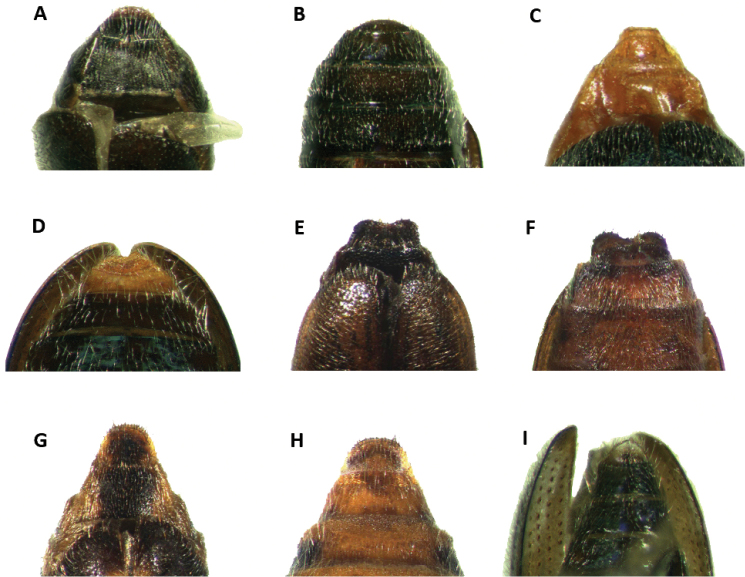
Pygidium of: **A** *Neocallotillus elegans* (*vafer*) dorsal (female) **B** *Neocallotillus elegans* (*vafer*) ventral (female) **C** *Cymatoderella patagoniae* dorsal (male) **D** *Cymatoderella patagoniae* ventral (male) **E** *Monophylla terminata* dorsal (male) **F** *Monophylla terminata* ventral (male) **G** *Monophylla terminata* dorsal (female) **H** *Monophylla terminata* ventral (female) **I** *Onychotillus vittatus* ventral (male).

**Figure 18.**
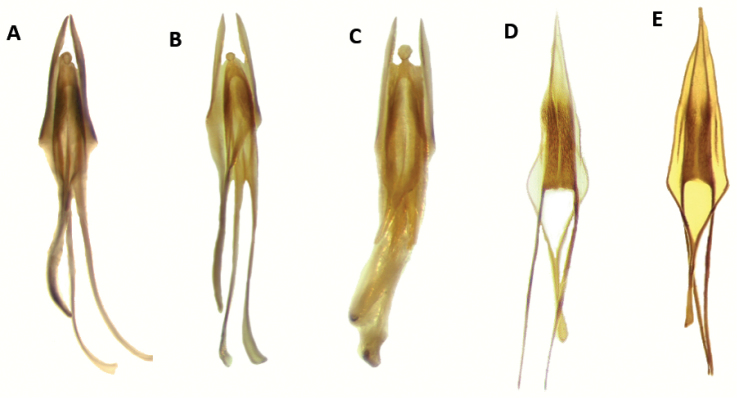
Male genitalia of: **A** *Araeodontia isabellae* **B** *Araeodontia marginalis* **C** *Araeodontia peninsularis* **D** *Bogcia disjuncta* **E** *Bogcia oaxacae* syn. n.

**Figure 19.**
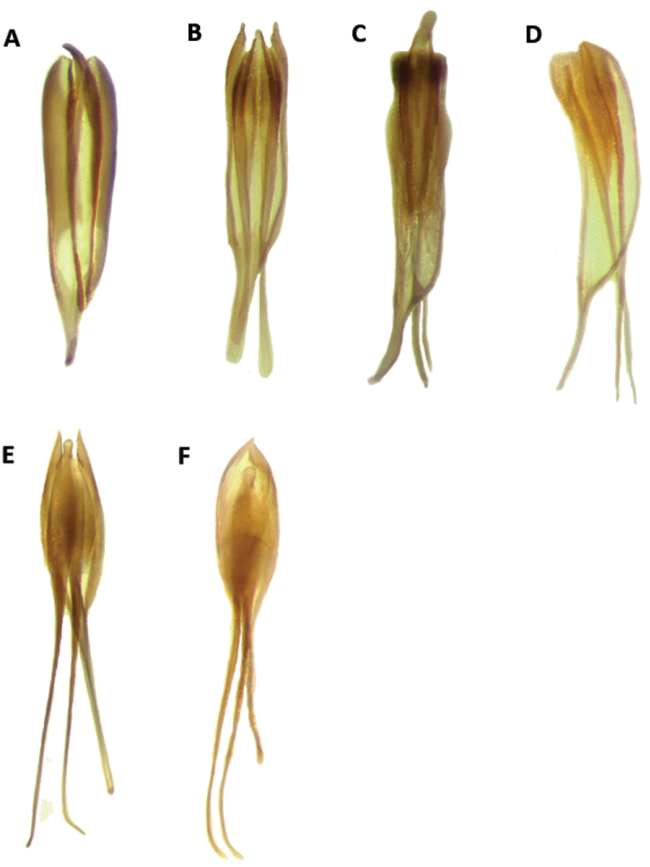
Male genitalia of: **A** *Neocallotillus elegans* (*vafer*) **B** *Cymatoderella collaris* **C** *Cymatoderella morula* **D** *Cymatoderella patagoniae* **E** *Lecontella brunnea* **F** *Lecontella gnara*.

**Figure 20.**
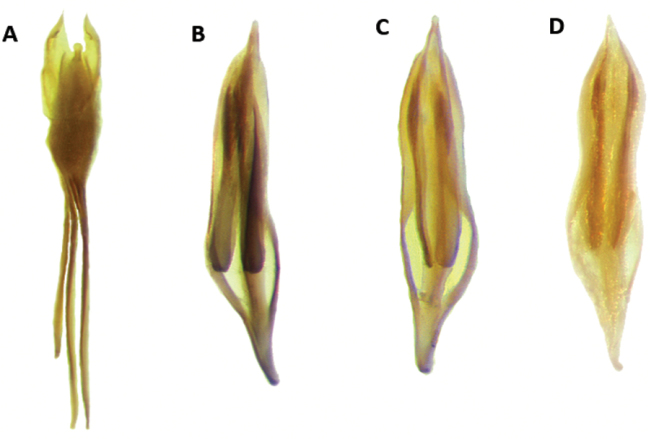
Male genitalia of: **A** *Lecontella striatopunctata* **B** *Monophylla californica* **C** *Monophylla pallipes* **D** *Monophylla terminata*.

#### Material examined.

1 male, 2 females: Dominican Republic, Provincia La Vega, La Cienega de Manabao Park Headquarter, 3-5-VII-1999, 3000’, R. E. Woodruff, backlight; 1 female: Constanza, Santo Domingo, 5000’, IX-1922, [no collector data]; 2 females: Jamaica, Bull Run, St. Andrew Park, 19-IV-1959, Farr and Sanderson.

**Figure 21.**
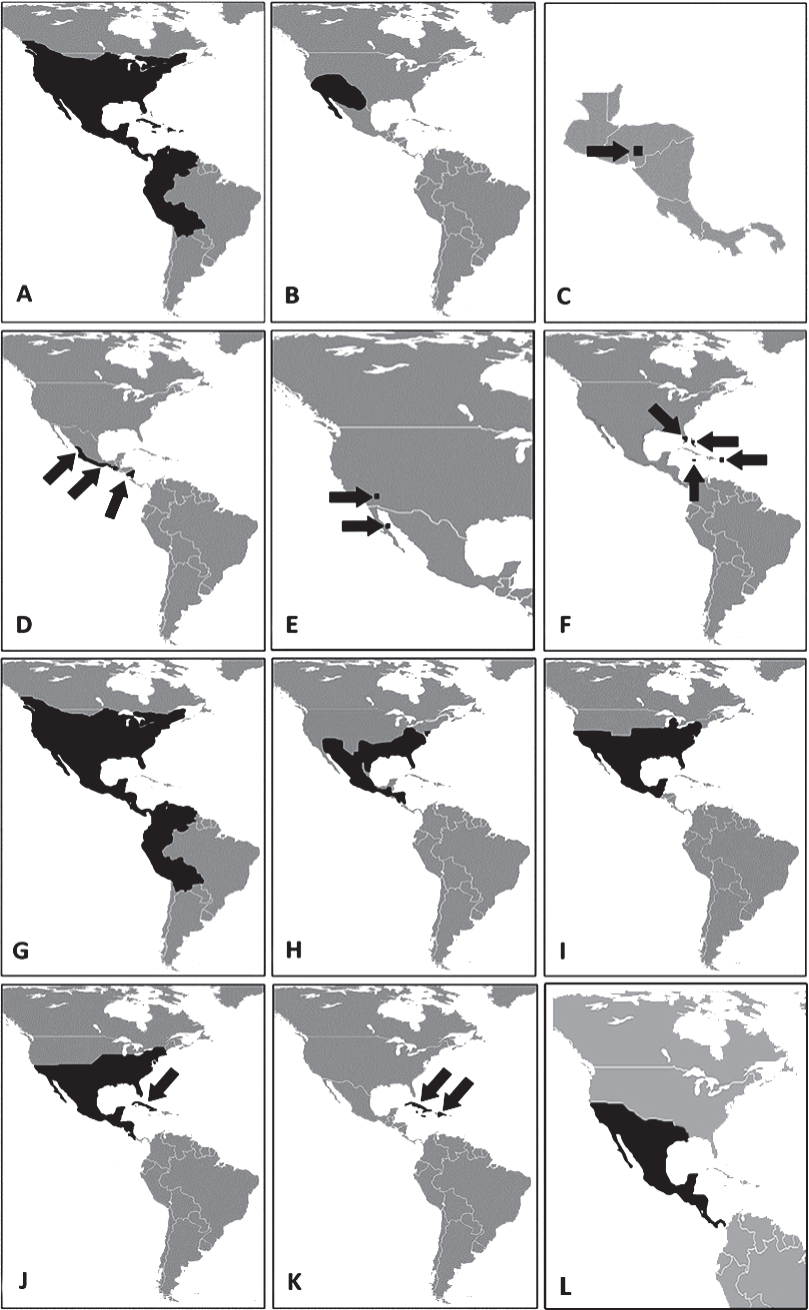
Distribution in the New World of: **A** Tillinae Leach **B** *Araeodontia* Barr **C** *Barrotillus* Rifkind **D** *Bogcia* Barr **E** *Bostrichoclerus* Van Dyke **F** *Callotillus* Wolcott **G** *Cymatodera* Gray (not treated here) **H** *Cymatoderella* Barr **I** *Lecontella* Wolcott & Chapin **J** *Monophylla* Spinola **K** *Onychotillus* Chapin **L** *Neocallotillus* Burke.

## Supplementary Material

XML Treatment for Tillinae

XML Treatment for Araeodontia

XML Treatment for Araeodontia isabellae

XML Treatment for Araeodontia marginalis

XML Treatment for Araeodontia peninsularis

XML Treatment for Araeodontia picipennis

XML Treatment for Araeodontia picta

XML Treatment for Barrotillus

XML Treatment for Barrotillus kropotkini

XML Treatment for Bogcia

XML Treatment for Bogcia disjuncta

XML Treatment for Bostrichoclerus

XML Treatment for Cylidrus

XML Treatment for Cylidrus abdominalis

XML Treatment for Cymatoderella

XML Treatment for Cymatoderella collaris

XML Treatment for Cymatoderella morula

XML Treatment for Cymatoderella patagoniae

XML Treatment for Lecontella

XML Treatment for Lecontella brunnea

XML Treatment for Lecontella gnara

XML Treatment for Lecontella striatopunctata

XML Treatment for Monophylla

XML Treatment for Monophylla californica

XML Treatment for Monophylla cinctipennis

XML Treatment for Monophylla pallipes

XML Treatment for Monophylla terminata

XML Treatment for Onychotillus

XML Treatment for Onychotillus cubana

XML Treatment for Onychotillus vittatus
